# Single-cell multi-omics analysis reveals cancer regulatory elements of transcriptional programs and clinical implications

**DOI:** 10.1038/s41419-025-08060-7

**Published:** 2025-10-21

**Authors:** Xiaowei Tang, Qiaoling Zhang, Zichu Shen, Jian Xiao, Minghao Li, Xiangyan Meng, Chenyi Wang, Guangze Zhang, Anhang Liu, Yuxin Yin

**Affiliations:** 1 Department of Pathology, Institute of Systems Biomedicine, School of Basic Medical Sciences, Peking-Tsinghua Center for Life Sciences, Peking University Health Science Center, Beijing, 100191 China; 2 Department of Gastroenterological Surgery, Peking University People’s Hospital, Beijing, 100044 China; 3 Institute of Precision Medicine, Peking University Shenzhen Hospital, Shenzhen, 518036 China

**Keywords:** Cancer epigenetics, Cancer microenvironment

## Abstract

The regulatory mechanisms governing transcriptional programs in the cancer genome remain elusive, particularly those concerning cell-type specificity. We carefully curated single-cell assay for transposase-accessible chromatin sequencing (scATAC-seq) and single-cell RNA sequencing (scRNA-seq) data from eight distinct carcinoma tissues, including breast, skin, colon, endometrium, lung, ovary, liver, and kidney. Using single-cell multi-omics analysis, we identified extensive open chromatin regions and constructed peak-gene link networks, which can reveal distinct cancer gene regulation and genetic risks. We further explored conserved epigenetic regulation across cell types within cancer and elucidated their functional implications. Moreover, we identified cell-type-associated transcription factors (TFs) that regulate key cellular functions, such as the TEAD family of TFs, which widely control cancer-related signaling pathways in tumor cells. In colon cancer, we further identified tumor-specific TFs that are more highly activated in tumor cells than in normal epithelial cells, including *CEBPG*, *LEF1*, *SOX4*, *TCF7*, and *TEAD4*, which are pivotal in driving malignant transcriptional programs and represent potential therapeutic targets, as corroborated by single-cell sequencing data from multiple sources and in vitro experiments. Our findings provide a comprehensive understanding of the regulatory dynamics underlying carcinomas and offer valuable insights into potential therapeutic interventions.

## Introduction

Carcinomas are a highly heterogeneous group of diseases, involving different cellular components within the complex milieu of the tumor ecosystem that each plays a pivotal role in tumor initiation and progression. While single-cell genomics technologies have improved our ability to decipher the cellular intricacies of tumors, much of the attention has focused on transcriptomics using single-cell RNA-seq (scRNA-seq) [1, 2]. The seminal work of The Cancer Genome Atlas (TCGA) and others has underscored the indispensable role of the epigenome in the cancer landscape [3, 4], with non-coding genomic regions containing regulatory elements exerting a profound influence on tumor biology. These regulatory sequences control the expression patterns of target genes by recruiting cell-type-specific transcription factors (TFs) [5]. Despite the progress we have made in our understanding, conventional assays still predominantly utilize heterogeneous tissues that provide population-level measurements, highlighting the need for a detailed exploration of the regulatory landscape of cancer within different cell types [3, 4]. Therefore, deciphering the regulatory programs operating in specific cell types within tumors promises novel insights into the molecular basis of tumor biology and heterogeneity.

Genome-wide association studies (GWAS) have identified thousands of genetic variants associated with different types of cancer. Many of these variants are located in non-protein-coding regions of the genome [6, 7], and are frequently enriched in predicted transcriptional regulatory regions, suggesting their potential impact on disease risk by modulating the regulation of target genes [8]. While bulk-tissue ATAC-seq has attempted to interpret the molecular functions of GWAS signals, its resolution is not sufficient to allow characterization of the specific cell types or gene expression levels influenced by these single nucleotide polymorphisms (SNPs) [9].

Single-cell assay for transposase-accessible chromatin sequencing (scATAC-seq) can identify accessible chromatin regions by Tn5 transposase-mediated tagmentation and captures active DNA regulatory elements at single-cell resolution [10]. Several studies employing scATAC-seq techniques have provided insights into diverse tumor tissues, uncovering specific regulatory landscapes that control tumor cells [11, 12]. The integration of scATAC-seq with single-cell RNA sequencing (scRNA-seq) augments the ability to explore gene regulation across various cell types, offering a more panoramic view of genome-wide regulatory elements and insights into TF binding and activity.

In this study, we carefully curated scATAC-seq and scRNA-seq data of different cancer types from several studies [11, 13–22], to identify cell-type-specific regulatory molecules that mediate tumor biology. By analyzing 380 465 cells from primary tumor tissues, we identified numerous candidate *cis*-regulatory elements (cCREs) based on chromatin accessibility. We also established a gene regulatory network by integrated analysis of scATAC-seq and scRNA-seq datasets, revealing distinct cancer gene regulation and genetic risks. Finally, we identified cell-type-associated TFs and discovered that tumor-specific TFs regulate malignant transcriptional programs, suggesting them as potential therapeutic targets.

## Materials and methods

### Patient samples

Colon cancer samples were obtained from the primary tumor and adjacent normal colon tissues after specimen resection at Peking University People’s Hospital. All sampling and experimental steps in this study were approved by the Ethics Committee of Peking University People’s Hospital (License 2021PHB088-001), and all patients signed written informed consent for this study.

### Tissue dissociation and library preparation

We used human colon cancer and adjacent normal colon tissues for single-cell multiome sequencing, with all steps performed at 4 °C. A frozen tissue fragment (approximately 50 mg) was placed into a pre-chilled 2-mL Dounce homogenizer containing 2 mL 1× homogenization buffer (320 mM sucrose, 0.1 mM EDTA, 0.1% NP40, 5 mM CaCl_2_, 3 mM Mg(Ac)_2_, 10 mM Tris-HCl pH 7.8, 167 μM β-mercaptoethanol, 1× protease inhibitor cocktail (Roche), and 1 U/μL RNase inhibitor (Thermo)). The tissue was homogenized approximately 15 strokes with the loose ‘A’ pestle, then filtered through a 70-μm nylon mesh to remove larger debris, followed by 20 strokes with tight ‘B’ pestle. Connective tissue and residual debris were excluded by filtration through a 40-μm nylon mesh filter followed by centrifugation at 350 r.c.f for 5 min.

The supernatant was aspirated and resuspended in 400 μL of 1× homogenization buffer. An equal volume of 50% iodixanol in homogenization buffer was added to a final concentration of 25% iodixanol. 600 μL of a 29% iodixanol solution (in 1× homogenization buffer containing 480 mM sucrose) was layered underneath the 25% iodixanol, and then a further 600 μL of a 35% iodixanol solution (in 1× homogenization containing 480 mM sucrose) was layered underneath the 29% iodixanol layer. After centrifugation in a swinging-bucket centrifuge at 3000 r.c.f for 35 min, the nuclei at the interface of the 29 and 35% iodixanol solutions were collected in a volume of 200 μL and counted using trypan blue.

500,000 nuclei were washed in buffer (10 mM Tris-HCl pH 7.4, 10 mM NaCl, 3 mM MgCl_2_, 1% BSA, 0.1% Tween-20, 1 mM DTT, and 1 U/μL RNase Inhibitor), followed by centrifugation at 500 r.c.f for 5 min. Nuclei were resuspended in 50 μL Diluted Nuclei Buffer (1× Nuclei Buffer*, 1 mM DTT, 1 U/μL RNase Inhibitor), and the concentration of nuclei was determined. 15,000 nuclei were aspirated for library construction and sequencing. Each nuclei suspension was submitted for library preparation using the Chromium Next GEM Chip J Single Cell Kit (PN-1000234, 10× Genomics) and the Chromium Next GEM Single Cell Multiome ATAC + Gene Expression Reagent Kits (PN-1000283, 10× Genomics), according to the manufacturer’s instructions. Finally, the libraries were sequenced using an Illumina Novaseq6000 sequencer with a sequencing depth of at least 50,000 reads per cell with the paired-end 150 bp strategy.

### Data collection

We collected single-cell sequencing data based on the following prioritized criteria: (1) Data generated using the 10× Genomics single-cell platform were selected. (2) Epithelial-origin tumors were included. (3) Entire tumor samples were sequenced rather than selectively sorting specific cell types, such as immune cells. (4) scATAC-seq and scRNA-seq data were generated from the same tumor samples. (5) Data from multiple studies were included whenever possible, while ensuring that batch effects between studies were minimal. (6) Sequencing data containing both tumor and normal tissues were selected from the same study. (7) For certain cancer types where only scATAC-seq data were available, corresponding scRNA-seq data were collected separately to supplement the analysis. The selected scATAC-seq and scRNA-seq datasets for BC, BCC, CC, EC, LC, OC, PLC, and RCC are summarized in Supplementary Table S1. Bulk ATAC-seq and methylation sequencing datasets were downloaded from the UCSC Xena website (https://xena.ucsc.edu/).

### Single-cell ATAC-seq data processing

The MACS2 method [23] was used to identify accessible chromatin regions in each fragment file, creating a unified set of peaks for quantification across all datasets. scATAC-seq data was analyzed using the Signac [24] R package (version 1.6.0). Low-quality cells were excluded based on the following criteria: nCount_peaks >2000, nCount_peaks <30,000, nucleosome signal <4, and TSS enrichment >2. To reduce computational resource usage, we randomly subsampled 30,000 cells for scATAC-seq data of BC. The Signac processing pipeline was applied, and cluster annotation was performed by comparing differential accessible regions associated with marker genes for tumor cells (*LGR5*, *EPCAM*, *CA9*), T cells (*CD247*), plasma cells (*JCHAIN*), myofibroblasts (*ACTA2*), myeloid cells (*ITGAX*, *CD163*), mast cells (*KIT*), fibroblasts (*PDGFRA*), endothelial cells (*EMCN*, *PECAM1*), and B cells (*MS4A1*). Gene activity matrix for scATAC-seq data was calculated using the GeneActivity function in Signac. To remove batch effects, individual datasets were harmonized using the Harmony algorithm [25]. Genomic regions with accessible chromatin peaks were annotated using the ChIPSeeker [26] R package (version 1.28.3) and the UCSC database on hg38.

### Single-cell RNA-seq data processing

The Seurat [27] R package (version 4.1.0) was used for the analysis of scRNA-seq data. Low-quality cells were excluded based on the following criteria: nCount_RNA < 50,000, nCount_RNA > 500, nFeature_RNA > 500, nFeature_RNA < 6 000, and % mitochondria < 25. Additionally, the DoubletFinder [28] R package (version 2.0.3) was independently applied to each library to identify potential doublets, and cells predicted to be doublets were excluded. The doublet rate increases by 0.8% for every 1000-cell increment. For scRNA-seq data of BC, CC, and LC, we used the processed data provided by the authors. To reduce computational resource usage, we randomly subsampled 30,000 cells for scRNA-seq data of BC. The default Seurat workflow was used for dimensionality reduction and clustering analysis. The same marker genes used in the scATAC-seq data analysis were employed for cell cluster annotation. To address batch effects across different datasets, the Harmony algorithm was used.

### Single-cell multiome-seq data processing

The raw sequencing data were initially processed using the cellranger-arc software (version 2.0.2, 10× Genomics) for tasks such as demultiplexing, alignment to the GRCh38 human reference genome, and generating gene-barcode matrices. MACS2 was then used to identify accessible chromatin regions in each fragment file and create a unified set of peaks for quantification across all datasets. For filtering, normalization, dimensionality reduction, clustering, and differential expression analysis, the Seurat and Signac R packages were applied. To ensure data quality, low-quality cells were excluded based on the following parameters: nCount_ATAC < 20,000, nCount_ATAC > 1 000, nCount_RNA < 25,000, nCount_RNA > 500, nFeature_RNA > 500, nFeature_RNA < 6000, TSS enrichment >2, and % mitochondria <25.

### Copy number alteration detection

Copy number alterations of epithelial cells from tumor tissues were inferred using InferCNV [29] R package (version 1.8.1) based on single-cell transcriptomic profiles. Non-tumor cells from scRNA-seq data served as the reference for CNV calling. The cutoff was set to 0.1 and the other parameters were kept at their default values.

### Genomic coordinate overlap analysis

To explore the DNA dictionary established by scATAC-seq, bulk ATAC-seq datasets and bulk DNA methylation datasets were obtained from TCGA through UCSC. The findOverlaps function in Signac was used to identify overlaps between peaks from scATAC-seq data and those from bulk ATAC-seq data, with a minimum of 1 bp overlap defining the genomic coordinate overlap. For the DNA methylation data, a direct comparison was made to determine whether methylation sites were located within the identified peak regions.

### Identify cell-type-specific cancer-associated differential accessible regions

Cell-type-specific accessible chromatin regions in each cancer type were identified using the FindAllMarkers function in Signac. A differential accessible region (DAR) was deemed cell-type-specific and cancer-associated if it appeared exclusively in one cancer type. Genomic coordinate overlap between DARs was defined as a minimum of 1 bp overlap. To better assess the heterogeneity of cell types, the mean proportion of cell-type-specific cancer-associated DARs relative to cell-type-specific DARs was calculated.

### Estimating GWAS enrichment using cell-type-specific accessible chromatin regions

We retrieved cancer-associated SNPs identified in various published genome-wide association studies from the NHGRI-EBI GWAS catalog [30]. To assess the heritability of diverse complex traits, we employed LDSC (version 1.0.1) [31]. GWAS summary statistics served as input for LDSC, enabling the computation of heritability enrichment for an annotated set of SNPs conditioned on a baseline model. This accounts for genomic features influencing heritability and jointly models multiple annotations. Cell-type-specific peaks were formatted for LDSC using the make_annotation.py script, and LD scores were computed for each set using the ldsc.py script. Publicly available GWAS summary statistics were gathered for a range of traits, including type 2 diabetes (T2D) [32], systemic lupus erythematosus (SLE) [33], rheumatoid arthritis (RA) [34], ulcerative colitis (UC) [35], Crohn’s disease (CD) [35], coronary atherosclerosis (CAD) [32], body height (BH) [36], body mass index (BMI) [37], balding [38], CC [39] and OC [32]. Subsequently, summary statistics were converted to hg38 coordinates using the UCSC liftover tool and formatted for LDSC using the munge_sumstats Python script. The recommended guidelines for cell-type-specific partitioned heritability analysis were followed, utilizing HapMap3 SNPs and the provided hg38 baseline model (v2.2). The ldsc.py script was then employed to compute cell-type-specific enrichments of GWAS heritability.

### Cis-co-accessible network construction

The correlation structure of chromatin accessibility data was scrutinized using the cicero [40] R package (version 1.3.9). Cicero estimates co-accessibility between peak pairs using a graphical LASSO algorithm, with a maximum interaction constraint of 500 kb. Importantly, prior to correlation and regularization, a bootstrap approach was implemented to generate metacells. This involved aggregating 50 cells at a time using k-nearest neighbors, effectively addressing the sparsity inherent in single-cell chromatin data.

### Peak-gene links analysis

A peak-gene correlation analysis was performed using a method similar to that described previously [41]. In brief, grouped scATAC-seq and grouped scRNA-seq data were used to identify peak-gene links, incorporating log-normalized accessibility and expression. For each gene in GenomicRanges, the distance to the start site was adjusted, and the start site was resized within a ± 500 kb window. For each chromosome, 1000 peaks not on the same chromosome were identified and correlated with all genes on that chromosome. Each potential peak-gene correlation was transformed into a z-score using the mean and standard deviation of the null trans correlations. These scores were then converted into *p*-values and adjusted for multiple-hypothesis testing using the Benjamini-Hochberg correction (‘p.adjust’ function in R).

To construct the peak-gene link networks, peak-promoter co-accessibility was computed using cicero, and peak-gene correlation was determined using the method described above. Reliable peak-gene links were defined by a high co-accessibility score (co-accessibility score >0.2) between peaks and the promoter of genes, as well as a significant correlation (FDR < 0.1) between peaks and genes. For OC, the FDR cutoff was set at 0.2 to obtain robust results.

### Identification of cell-type-conserved regulatory programs

Cell-type-conserved regulatory programs were identified using the iterative overlap method, which is implemented in ArchR [42]. Briefly, cell-type-associated regulatory regions were obtained by intersecting regulatory regions from regulatory networks with cell-type-associated DARs in each cancer type. The iterative overlap method was then employed to generate cell-type-associated peak sets for cell-type-associated regulatory regions of the same cell type. Finally, cell-type-associated peak sets were intersected with the cell-type-associated regulatory regions from each cancer type, removing peaks that appeared in only one cancer type.

### Motif analysis and transcription factor footprinting

Single-cell TF motif enrichment analysis was performed for 633 human TFs from the JASPAR 2020 database [43] using the Signac wrapper for chromVAR [44]. Initially, a motif accessibility matrix was computed to estimate the number of peaks containing each TF motif across all cells. Subsequently, chromVAR utilized this motif accessibility matrix to calculate deviation Z-scores for each motif. This calculation involved a comparison between the actual number of peaks containing the motif and the expected number of fragments in a background set. It also accounted for potential confounding factors, such as GC content bias, PCR amplification, and variability in Tn5 tagmentation. For a more detailed analysis of specific TFs of interest, the Footprint function in Signac was employed to conduct TF footprinting analysis in pseudo-bulk aggregates of single cells within the same cell type. Normalized values of TF footprinting were obtained from the GetFootprintData function in Signac.

### Identification of intratumor NMF programs

The NMF method [42], implemented by the NMF R package (version 0.24.0), was utilized to identify the underlying transcriptional programs within tumor cells from three samples (A001-C-007, CRC-1-8810, CRC-3-11773; number of tumor cells >500). We applied this approach (k = 2:6, nrun = 30) to the relative expression matrix of tumor cells in each sample, with all negative values converted to zero. The optimal k value, signifying a robust clustering result, was determined based on the point at which the cophenetic coefficient exhibited the maximum drop. Subsequently, 10 distinct programs across the three samples were identified. For each program, the top 100 genes with the highest NMF scores were utilized to score tumor cells in each sample, employing the Seurat AddModuleScore function. The correlations between these program scores were calculated individually for each sample. Finally, four meta-programs were identified through hierarchical clustering of averaged correlations of pairs of programs across all samples, using Pearson correlation as the distance metric and Ward’s linkage. Clustering groups covering less than half of the samples were disregarded. The 100 genes with the highest average NMF score within each highly correlated program set were selected to represent the corresponding meta-program. Pathway enrichment analysis for each meta-program was conducted using the clusterProfiler [45] R package (version 4.0.5). Regulatory elements for genes within each meta-program were identified using the peak-gene link network. Candidate TFs for each gene were defined based on DNA sequence motifs present in the regulatory elements of the gene.

### Analysis of meta-program dependencies

For the dependency analysis of genes from each meta-program, we manually searched the Cancer Dependency Map Project database for colon cancer cell lines [46]. Dependency data from primary cancer cell lines were used to estimate cellular viability.

### Perturbagen’s signature of tumor-specific TFs

Perturbagen’s signatures of tumor-specific TFs were downloaded from the Connectivity Map [47]. Perturbagen’s signatures of *CEBPG*, *SOX4*, *TCF7*, and *TEAD4* were obtained from the HT29 cell line, whereas perturbagen’s signature of *LEF1* was obtained from all cell lines in the database.

### Identification of drug targeting transcription factor

Drug perturbed profiles generated from the LINCS consortium [47] were used to screen candidate drugs targeting *CEBPG*, *LEF1*, *SOX4*, *TCF7*, and *TEAD4*. These profiles were downloaded from the ExperimentHub [48] R package (version 2.0.0), which includes a Z-score matrix from differential expression analysis of 12,328 genes across 8140 compound treatments. Genes with a Z-score smaller than –2 were considered differentially downregulated genes. Candidate drugs were defined as those that can significantly downregulate the expression of the target TF. Finally, tacedinaline, quinoclamine, dorsomorphin, and vorinostat were selected as candidate drugs for targeting *CEBPG*, *LEF1*, *SOX4*, and *TEAD4*, respectively.

### Cell culture and reagents

All human cell lines used in this study were sourced from the American Type Culture Collection. Authentication of these cell lines was conducted through STR locus analysis, and they were regularly screened for mycoplasma contamination. HEK293T cells and DLD1 cells were cultured in DMEM (Corning), supplemented with 10% FBS (HyClone), at 37 °C 5% (v/v) CO_2_.

### Vectors and lentiviral transfection

All short hairpin RNAs (shRNA) were cloned into pLKO.1-TRC vector. Target sequences were as follows:

*CEBPG*: TTAGCCTTGTAATTCGAATAT;

*LEF1*: CCATCAGATGTCAACTCCAAA;

*SOX4*: GAAGAAGGTGAAGCGCGTCTA;

*TEAD4*: GAGACAGAGTATGCTCGCTAT;

*TCF7*: CAACTCTCTCTCTACGAACAT.

Lentiviral particles were generated by transfecting the shRNA plasmid, packaging vectors psPAX2 and pMD2.G into HEK293T cells at a ratio of 3:2:1 using jetPRIME Transfection Reagent (Polyplus-transfection Inc.). The media was replaced 12 h post-transfection. 48 h later, the media containing the virus were collected. After centrifugation at 1500× g for 45 min, the pellets containing virus were resuspended with DMEM medium. The efficiency of lentiviral shRNA clones was assessed by real-time PCR.

### Lentiviral infection

A total of 1 × 10^6^ DLD1 cells were plated in each well of 12-well plates. 200 μL of lentivirus and 10 μg/mL polybrene were added at the same time. After 12 h at 37 °C, the cells were transferred to new culture dishes. 24 h later, the lentivirus-infected cells were subjected to selection with puromycin (Invivogen) for 3 days.

### RNA isolation and qRT-PCR

Total cellular RNA was extracted using TRIzol Reagent (Vazyme) and RNA quantified with NanoDrop 2000 (Thermo Fisher Scientific, USA). Subsequently, total RNA was reverse transcribed using Promega cDNA Synthesis SuperMix (Promega) following the manufacturer’s guidelines. Quantitative reverse transcription PCR (qRT-PCR) was conducted according to the manufacturer’s protocol on the Bio-Rad CFX ConnectTM system, utilizing SYBR Green Master Mix (Vazyme) and gene-specific primers. The qPCR primer sequences were as follows:

*CEBPG*-fwd: 5′-ACTCCAGGGGTGAACGGAAT-3′;

*CEBPG*-rev: 5′-CATGGGCGAACTCTTTTTGCT-3′;

*LEF1*-fwd: 5′-AGAGCCCCAATATCCCTGC-3′;

*LEF1*-rev: 5′-GAGAAAAGTGCTCGTCACTGT-3′;

*SOX4*-fwd: 5′-AGCGACAAGATCCCTTTCATTC-3′;

*SOX4*-rev: 5′-CGTTGCCGGACTTCACCTT-3′;

*TEAD4*-fwd: 5′-GAACGGGGACCCTCCAATG-3′;

*TEAD4*-rev: 5′-GCGAGCATACTCTGTCTCAAC-3′;

*TCF7*-fwd: 5′-AACACCTCAACAAGGGCACTC-3′;

*TCF7*-rev: 5′-CCCCACTTGAAACGGTTCCT-3′.

### Western blot

Cells were lysed in RIPA lysis buffer with protease inhibitor cocktail (Abcam). BCA protein quantification kit (Beijing Solarbio Science & Technology) was used to quantify the protein concentration. Equal amounts of protein were separated by SDS-PAGE and transferred to PVDF membrane. After blocking with 5% BSA for 1 h, the membranes were incubated overnight at 4 °C with the indicated primary antibodies, followed by secondary antibody. Primary antibodies were used in this study: anti-CEBPG (UpingBio), anti-LEF1 (Sangon Biotech), anti-SOX4 (UpingBio), anti-TCF7 (Sangon Biotech), anti-TEAD4 (IPODIX), anti-GAPDH (Sungene Biotech).

### Cell proliferation assay

A seeding density of 2000 cells was employed for each well in 96-well plates, and the cells were allowed to adhere. At designated time points, the culture medium was substituted with CCK-8 solution (Beyotime), followed by incubation at 37 °C for 2 h. Absorbance values were measured at 450 nm using a Thermo Fisher Scientific Varioskan Flash multimode reader, and cell proliferation curves were generated based on the absorbance at each time point. Each group was established with six replicates.

### Drug treatment

To assess the mRNA and protein level of the targeted gene induced by drug treatment, 1 × 10^6^ DLD1 cells were seeded into each 10 cm cell culture dish. After 24 h of incubation at 5% CO_2_ and 37 °C, the cells were treated with 10 μM Tacedinaline (Targetmol), 10 μM Dorsomorphin (Macklin), 10 μM Vorinostat (Yeasen), or 4 μM Quinoclamine (Targetmol) for 3 days. Subsequently, the cells were harvested for the detection of mRNA levels of the targeted genes. For cell proliferation assays, 5 000 DLD1 cells were seeded into each well of 96-well plates with 200 μL of medium per well. After 24 h of incubation at 5% CO_2_ and 37 °C, the cells were treated with 10 μM Tacedinaline (Targetmol), 10 μM Dorsomorphin (Macklin), 10 μM Vorinostat (Yeasen), or 4 μM Quinoclamine (Targetmol) for 3 days. Cell proliferation was assessed every 24 h using the CCK-8 solution (Beyotime).

### Cell migration assay

A Transwell assay (8 μm, Corning) was used to evaluate the migration ability of tumor cells. Briefly, 1×10^5^ DLD1 cells were seeded in the upper chamber, while the lower chamber contained culture medium with 10% FBS. After 24 h of incubation, the membrane of the upper chamber was carefully removed by a cotton swab, fixed in 4% paraformaldehyde for 30 min at room temperature and then stained with crystal violet for 15 min. Migrated cells were quantified and counted under an inverted light microscope. Each group was established with three replicates.

### Cell apoptosis assay

For the apoptosis assay, cells were suspended with 100 μL binding buffer containing 5 μL of Annexin V-FITC and 5 μL of 7-AAD staining solution (SinoBiological). After staining for 15 min, 400 μL binding buffer was added to terminate the reaction. Flow cytometry was used to distinguish dead and apoptotic cell populations. Each group was established with three replicates.

### Statistical analysis

Enrichment analysis for differentially accessible chromatin regions was performed by GREAT [49] (version 4.0.4). Most of the computational analyses and statistical tests were performed in R version 4.1.0. The Wilcoxon test and Kruskal–Wallis test were used for between-group comparisons as appropriate, and *P* values < 0.05 were considered statistically significant.

## Results

### Single-cell transcriptional and chromatin accessibility profiling in diverse carcinomas

**Fig. 1:**
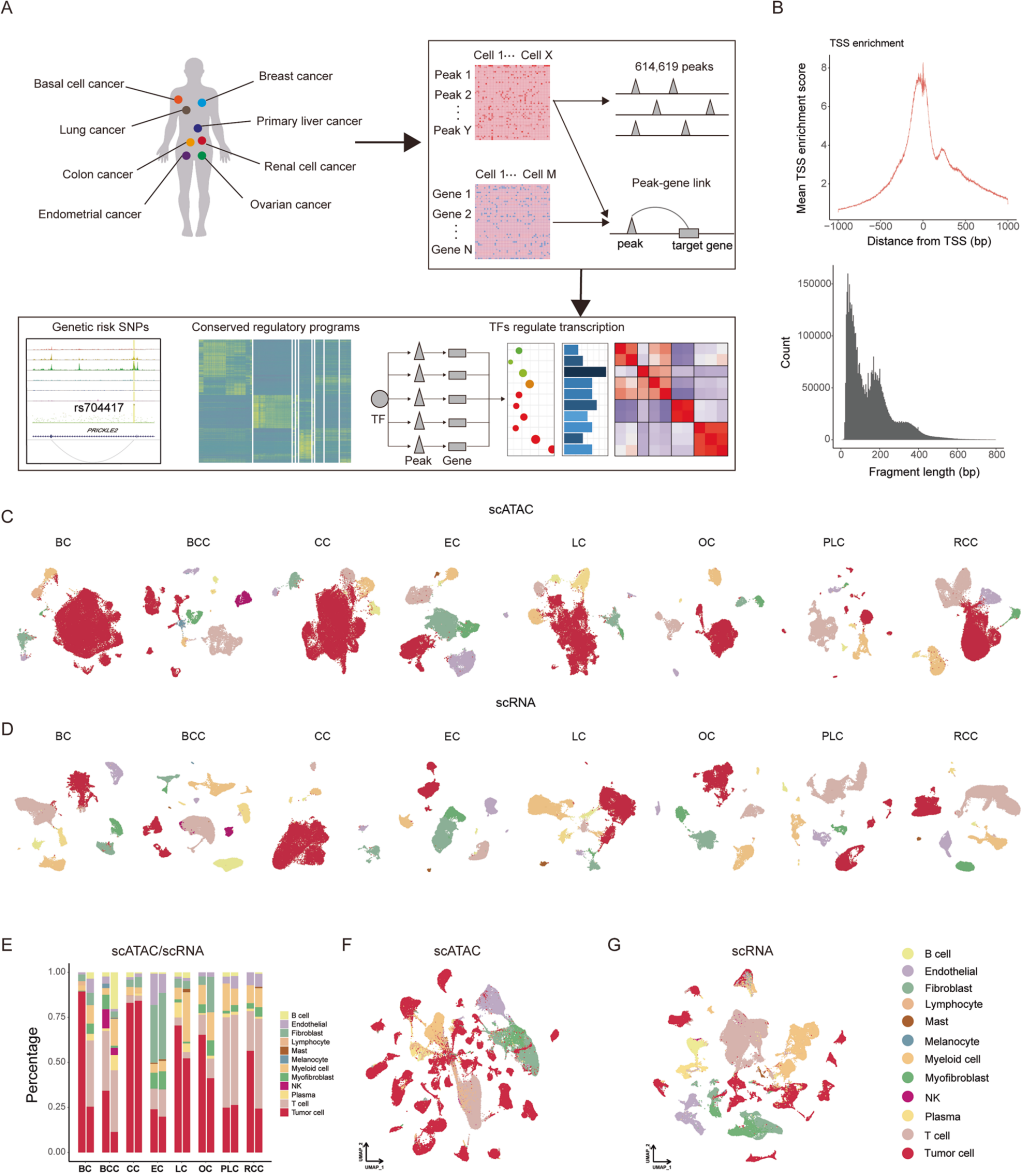
Overview of cellular composition across eight carcinomas through scATAC-seq and scRNA-seq analysis. **A** Schematic representation of the cancers used in this study, and downstream bioinformatic analyses. **B** Enrichment of scATAC-seq accessibility near transcription start sites (TSS) and fragment length distribution across all datasets. **C** UMAP plots of scATAC-seq cells color-coded by cell types found in eight carcinomas. **D** UMAP plots of scRNA-seq cells color-coded by cell types found in eight carcinomas. **E** Proportion of various cell types in scATAC-seq (left) and scRNA-seq (right) for eight types of carcinomas. UMAP plots show the embedding of integrated scATAC-seq data **F** and scRNA-seq data **G**.

To elucidate the chromatin and transcriptional features of human primary carcinomas, we curated publicly available scATAC-seq and scRNA-seq data, including breast cancer (BC), basal cell cancer (BCC), colon cancer (CC), endometrial cancer (EC), lung cancer (LC), ovarian cancer (OC), primary liver cancer (PLC), and renal cell cancer (RCC) (Fig. 1A, Supplementary Table S1). After rigorous quality control, a total of 176,928 cells from scATAC-seq and 203,537 cells from scRNA-seq were retained for subsequent analysis (Supplementary Table S2). A TSS plot of the data confirms a robust enrichment of unique open chromatin fragments at transcription start sites (TSS), while periodicity in fragment length reveals characteristic peaks indicative of high-quality ATAC-seq experiments (Fig. 1B). Cells were clustered for each cancer type separately and cell type identities were assigned to each cell. To integrate data from different samples, we used the Harmony algorithm [25] for both scATAC-seq and scRNA-seq data to correct batch effects. Cluster annotations were accomplished by comparing differentially accessible regions and differential gene expression with markers associated with various cell types, including tumor cells (*LGR5*, *EPCAM*, *CA9*, *KRT18*), T cells (*CD247*), NK cells (*NCR1*), plasma cells (*JCHAIN*, *FCRL5*), myofibroblasts (*ACTA2*), myeloid cells (*ITGAX*, *CD163*), mast cells (*KIT*), fibroblasts (*PDGFRA*), endothelial cells (*EMCN*, *PECAM1*, *PLVAP*), melanocytes (*MLANA*), and B cells (*MS4A1*) (Fig. 1C, D, Supplementary Fig. S1A, B, Table S3). At the same time, we identified primary copy number variation (CNV) events in each cell based on its transcriptomic profile to confirm the annotations of tumor cells by marker genes (Supplementary Fig. S1C). We presented the cell composition proportions for both scATAC-seq and scRNA-seq datasets in all cancer types (Fig. 1E). Moreover, we projected all the cells onto a UMAP, and cells identified as the same type clustered as expected (Fig. 1F, G).

**Fig. 2:**
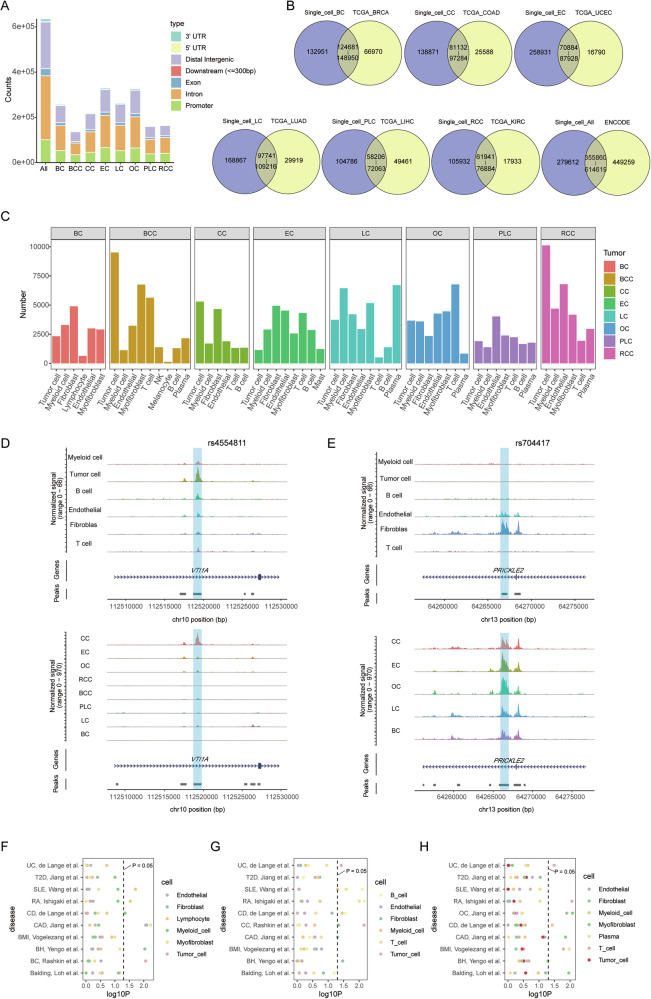
Single-cell ATAC-seq extends the compendium of DNA regulatory elements and reveals cancer genetic risks. **A** Quantification of peaks identified in individual cancer-type scATAC-seq datasets and the combined dataset. Colors correspond to the indicated peak types. **B** Venn diagram illustrating the intersection between cCREs identified in scATAC-seq data and the registry of cCREs created by other projects. This includes BC scATAC-seq data intersecting with TCGA-BRCA bulk ATAC-seq data, CC scATAC-seq data intersecting with TCGA-COAD bulk ATAC-seq data, EC scATAC-seq data intersecting with TCGA-UCEC bulk ATAC-seq data, LC scATAC-seq data intersecting with TCGA-LUAD bulk ATAC-seq data, PLC scATAC-seq data intersecting with TCGA-LIHC bulk ATAC-seq data, RCC scATAC-seq data intersecting with TCGA-KIRC bulk ATAC-seq data, and integrated scATAC-seq data intersecting with the ENCODE consortium. The numbers on the left, right, top and bottom represent the number of unique peaks in scATAC-seq data, the number of unique peaks in TCGA data, the number of overlapping peaks in scATAC-seq data, and the number of overlapping peaks in TCGA data, respectively. **C** The number of DARs for all cell types in the scATAC-seq data of eight carcinomas. **D** Normalized scATAC-seq tracks for all cell types in CC (upper) and the eight types of tumor cells (lower) at the SNP rs4554811 locus. **E** Normalized scATAC-seq tracks for all cell types in CC (upper) and the five types of fibroblasts (lower) at the SNP rs704417 locus. Dot plot showing the significance of enrichment for selected traits from BC **F**, CC **G**, and OC **H**, within DARs from different cell types. Each circle represents a cell type.

To identify variances in chromatin accessibility across diverse cell types, we identified 257,632 peaks in BC scATAC-seq data, 138,534 peaks in BCC scATAC-seq data, 220,003 peaks in CC scATAC-seq data, 329,815 peaks in EC scATAC-seq data, 263,338 peaks in LC scATAC-seq data, 326,761 peaks in OC scATAC-seq data, 162,992 peaks in PLC scATAC-seq data, and 167,873 peaks in RCC scATAC-seq data individually, utilizing MACS2 (Fig. 2A). Most of them were observed in more than 10 cells (Supplementary Fig. S2A). The predominant presence of these accessible peaks is noted in intronic, distal intergenic, as well as promoter regions of the genome. Intriguingly, our scATAC-seq approach detects more open chromatin regions than those identified by bulk-tissue ATAC-seq (Fig. 2B). We then merged the accessible chromatin regions to obtain a list of 614,619 non-overlapping peaks, which covered 61.5% of the regulatory elements in the registry of cCREs published by the ENCODE consortium [50] (Fig. 2B), suggesting that scATAC-seq extends the compendium of DNA regulatory elements. In accordance with published findings [3, 51], which establish an inverse correlation between DNA methylation and chromatin accessibility at regulatory elements, we examined the methylation data from TCGA to further delineate the accessible potential of the peaks identified. Notably, methylation sites within peak regions exhibit lower methylation β values, while those in non-peak regions display higher methylation β values (Supplementary Fig. S2B).

We then identified differentially accessible chromatin regions (DARs) across distinct cell types. Tumor cells, endothelial cells, and fibroblasts showed a higher prevalence of DARs, whereas myeloid cells, plasma cells, and B cells demonstrated comparatively fewer DARs (Fig. 2C, Supplementary Table S4). To examine the heterogeneity within the same cell type across different cancers, we identified cell-type-specific cancer-associated DARs. These analyses indicate that tumor cells exhibit the highest degree of heterogeneity across the eight types of cancer, followed by fibroblasts, while immune cells display minimal heterogeneity (Fig. S2C, Supplementary Table S4).

### Single-cell ATAC-seq can reveal distinct cancer genetic risks

To determine the impact of our identified accessible chromatin regions on the genetic variation associated with carcinomas, we scrutinized the NHGRI-EBI GWAS catalog for BC, CC, EC, LC, OC, PLC, and RCC, and noted a higher prevalence of SNPs within our identified accessible chromatin regions in BC, CC, and OC (Supplementary Fig. S2D). By overlaying the co-accessibility map with the chromatin accessibility signal and GWAS statistics along the genomic axis, we were able to identify known chromatin accessibility sites. SNP rs4554811 and rs704417 are associated with increased susceptibility to colon adenocarcinoma, consistent with the presence of focal chromatin accessibility in CC scATAC-seq data [52, 53]. SNP rs4554811 located intronically in *VTI1A*, exhibits heightened accessibility in colon tumor cells, with weak accessibility in other tumor cells, presenting its specific inherited risk in CC susceptibility (Fig. 2D). Additionally, SNP rs704417 shows strong accessibility in fibroblasts from CC, as well as from EC, OC, LC, and BC, suggesting a potential role for it in previously unappreciated cancer contexts (Fig. 2E). Further analysis revealed that numerous GWAS SNPs were open in multiple cancer types, suggesting that they play similar regulatory roles across different cancer types (Supplementary Table S5).

To further investigate the genetic risk signals for BC, CC, and OC, we performed cell-type-specific linkage-disequilibrium score regression (LDSC) analysis of our scATAC-seq clusters using GWAS summary statistics in BC, CC, OC, as well as other relevant traits (Fig. 2F–H, Supplementary Table S6). Intriguingly, immune-related diseases, such as systemic lupus erythematosus (SLE), rheumatoid arthritis (RA), ulcerative colitis (UC), and Crohn’s disease (CD), exhibit a notable correlation with immune cells. Additionally, fibroblasts and myofibroblasts exhibit associations with traits such as balding, coronary atherosclerosis (CAD), and body height (BH), consistent with prior investigations [54]. With respect to BC, GWAS SNPs were enriched in open regions from tumor cells, and in CC, both fibroblasts and tumor cells display a significant enrichment for GWAS SNPs, whereas in OC, enrichment was observed in fibroblasts and myofibroblasts. We further revealed that the expression of marker genes in CC fibroblasts, OC fibroblasts, and OC myofibroblasts presented significant correlations with overall survival, suggesting dysfunctions in these stromal cells contribute to a worse prognosis (Supplementary Fig. S3A–D).

### Differentially accessible regions are candidate DNA regulatory elements

**Fig. 3:**
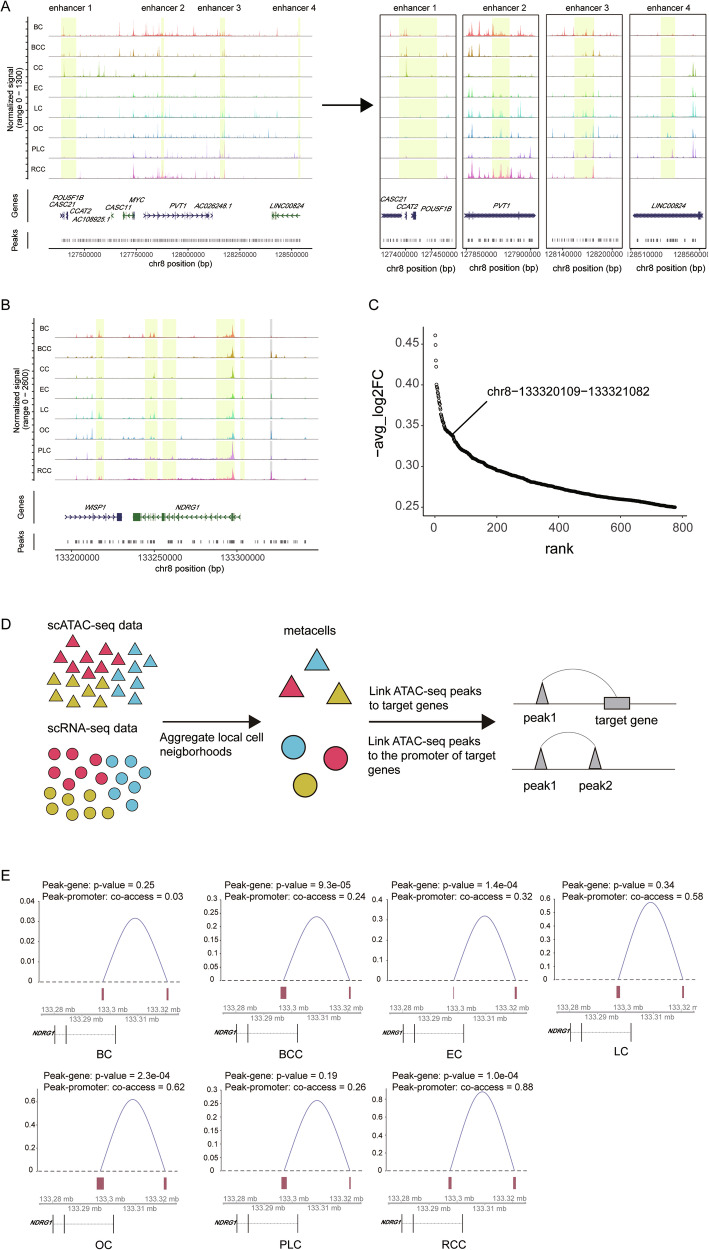
Identification of candidate regulatory elements. **A** Normalized scATAC-seq tracks for the eight types of tumor cells at the *MYC* locus. Enhancer regions of *MYC* are colored in yellow. **B** Normalized scATAC-seq tracks for the eight types of tumor cells at the *NDRG1* locus. Reported enhancer regions of *NDRG1* are shaded in yellow and the predicted enhancer region is marked in gray. **C** The rank of DARs for colon cancer cells with low accessibility. **D** Workflow of the construction for peak-gene link network. The triangle represents cells derived from scATAC-seq data, while the circle represents cells from scRNA-seq data. Different colors indicate distinct cell types. **E** Connection plots illustrating the interactions between the promoter region and predicted enhancer region for *NDRG1* in BC, BCC, EC, LC, OC, PLC, and RCC. The *p*-value of peak-gene represents the *p*-value from the peak-gene correlation analysis. The co-accessibility score of peak-promoter represents the co-accessibility score calculated by Cicero.

To investigate potential variations in regulatory dynamics across different cancer types, we visually examined our scATAC-seq data. The regulatory elements of *MYC*, known for their high degree of tissue specificity and distinct locations in different cancer types [55], served as our focal point. Our analysis revealed that *MYC* is widely expressed and its locus has extensive accessibility in various cell types from the tumor ecosystem (Supplementary Fig. S4A, B). Remarkably, tumor cells exhibit extensive chromatin accessibility at both the 5′ and 3′ DNA elements of *MYC*, whereas other cells primarily display accessibility at the 3′ DNA elements. Furthermore, we focused on tumor cells extracted from integrated scATAC-seq data (Supplementary Fig. S4C, D). Our finding revealed that tumor cells from BC, BCC, CC, EC, LC, and OC all have strong accessibility at both the 5′ and 3′ DNA elements of *MYC*, while PLC and RCC show accessibility at the 3′ regulatory elements, consistent with previous findings [3] (Fig. 3A). The comprehensive nature of our scATAC-seq data is underscored by its ability to reveal the enhancer regions of *MYC* in CC, RCC, LC, and OC, reinforcing its credibility (Fig. 3A). We next turned our attention to *NDGR1*, a gene known for its pivotal role in cancer development and progression, but for which a comprehensive understanding of its transcriptional regulation is lacking. Notably, the top 5 regulatory elements documented in GeneCards surface in our scATAC-seq data analysis (Fig. 3B). Differential accessibility of these regulatory elements across distinct tumor cells suggests varied regulatory influences. Additionally, we identified a region (chr8-133320109-133321082) exhibiting high accessibility in BCC, EC, OC, and RCC, which is conspicuously absent in CC cells (Fig. 3B, C).

To explore the interaction between DNA regulatory elements and target genes, we predicted peak-gene links through integrated scATAC-seq and scRNA-seq analysis (Fig. 3D). We calculated correlations between peak accessibility and gene expression from the integrated dataset. In parallel, we constructed a co-accessibility network for distal peaks and the promoters of target genes. Confident peak-gene links are contingent on significant peak-gene correlation and a high co-accessibility score (Fig. 3D). In BCC, EC, OC, and RCC, correlations with *NDRG1* expression have p-values of 9.3e-05, 1.4e-04, 2.3e-04, and 1.0e-04, respectively, with co-accessibility scores of 0.24, 0.32, 0.62 and 0.88 (Fig. 3E). However, in BC, LC, and PLC, the target region displays a low correlation with the expression of *NDRG1* (*p*-value = 0.25, 0.34, and 0.19). These findings suggest that the target region serves as a potential CRE of *NDRG1* in BCC, EC, OC, and RCC, while displaying no regulatory effect on *NDRG1* in BC, LC, and PLC. Intriguingly, subsequent examination of DARs across distinct tumor cells indicates a similarity with all exhibiting weak accessibility in the target region for BC, LC, and PLC (Supplementary Fig. S4E–G).

Furthermore, we systematically estimated the proportion of regulatory regions identified by gene regulatory networks relative to DARs of different cell types across diverse cancer types. Notably, five types of cells exhibited more than 500 DARs, of which approximately 10 to 60% were identified as candidate regulatory DNA elements (Supplementary Fig. S4H).

### Cell-type-associated epigenetic regulation in different carcinomas

**Fig. 4:**
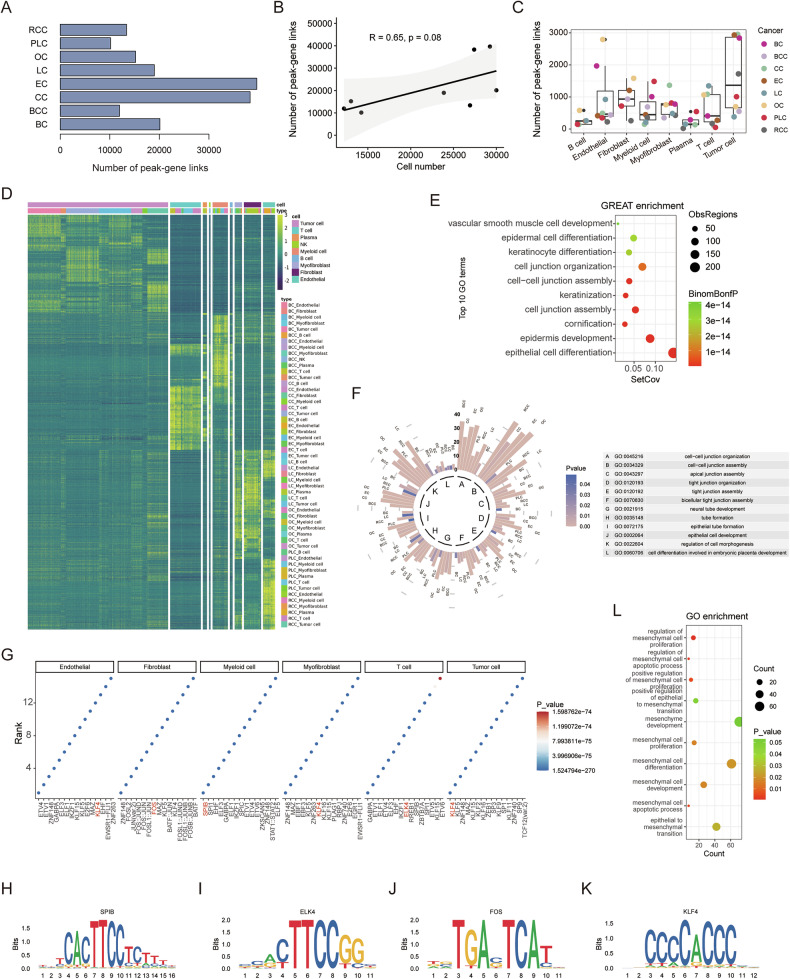
Cell-type-associated epigenetic regulation in the eight carcinomas. **A** Number of identified peak-gene links in the eight carcinomas. **B** The scatter plot and correlation between the cell number in scATAC-seq data and the number of peak-gene links (R represents Pearson’s correlation and its coefficient of determination, the p-value is 0.08). **C** Number of cell-type-associated peak-gene links in the eight carcinomas. **D** Heatmap representation of the cell-type-conserved regulatory elements predicted. Each row represents an individual scATAC-seq peak. Color represents the scATAC-seq log normalized accessibility for each peak. **E** GREAT ontology enrichment analysis of cell-type-conserved regulatory elements. **F** Circular bar plot of twelve overlapping pathways in the eight carcinomas. The length of the bar represents the number of genes in the indicated pathway, and the color intensity of the bar indicates the *p*-value. **G** Enriched motifs in different cell types. Motifs are ranked by *p*-values from smallest to largest. Motif plots of SPIB **H**, ELK4 **I**, FOS **J**, and KLF4 **K**. **L** Gene Ontology enrichment analysis of KLF4 regulatory genes about EMT-related pathways.

Using the approach described above, we identified 20,109, 11,955, 38,292, 39,640, 18,976, 15,202, 10,140, and 13,368 unique peak-gene link pairs in the single-cell data from BC, BCC, CC, EC, LC, OC, PLC, and RCC, respectively (Fig. 4A). The number of peak-gene link pairs correlates with the number of cells from single-cell ATAC-seq data (Fig. 4B). By examining the overlap between these linked peaks and the DARs identified in each cell type, we identified cell-type-associated regulatory regions (Fig. 4C), which can reveal the specific cell functions of cancer genetic risk loci. For instance, SNP rs4554811 and rs704417 exhibit predicted interactions with nearby transcript promoter regions, indicative of a potential regulatory role for *TCF7L1* and *PRICKLE2* (Supplementary Fig. S5A, B).

We further identified cell-type-conserved regulatory regions in different carcinomas, recognized as candidate regulatory elements not specific to a single carcinoma (Fig. 4D). Tumor cells, T cells, myeloid cells, myofibroblasts, fibroblasts, and endothelial cells present substantial conserved regulatory regions. To assess the potential functions of conserved epigenetic regulation in tumor cells, we employed GREAT ontology enrichment analysis on conserved chromatin regions and gene ontology (GO) enrichment analysis on their target genes. The results revealed that tumor-conserved regions predominantly regulate cell junction and epithelial cell development-related pathways (Fig. 4E, Supplementary Fig. S5C). Moreover, we individually inspected the functions of tumor cell-associated regulatory elements by performing GO enrichment analysis on their predicted regulatory genes. Twelve processes were enriched in all tumor cells, including pathways related to cell junction and epithelial cell development, consistent with previous findings (Fig. 4F, Supplementary Fig. S5D). We also explored the potential functions of conserved regulatory regions in other cell types. For instance, T cells mainly regulate T cell activation, myeloid cells influence the immune response, fibroblasts contribute to extracellular matrix organization, and both myofibroblasts and endothelial cells play roles in blood vessel development (Supplementary Fig. S5E, F).

We next applied motif enrichment analysis on cell-type-conserved regulatory regions to reveal key TFs of each cell type (Fig. 4G). Myeloid cells and T cells are enriched for the binding sites of expected cell-type-activated TFs, such as SPIB and ELK4. Fibroblasts, on the other hand, showed a strong enrichment for AP-1 TF binding sites (Fig. 4H, J). Notably, tumor cells exhibit an enrichment in binding sites for Krüppel-like factors, similar to endothelial cells and myofibroblasts. We identified tumor cell-conserved regulatory regions with KLF4 binding sites and performed GO enrichment analysis on the target genes of KLF4. This analysis revealed that genes regulated by KLF4 are significantly enriched in ontology terms related to epithelial-mesenchymal transition (EMT) (Fig. 4K, L), indicating that KLF4 plays a pivotal role in regulating genes associated with mesenchymal characteristics, consistent with previous studies [56].

### Transcription factor activities in different cell types

**Fig. 5:**
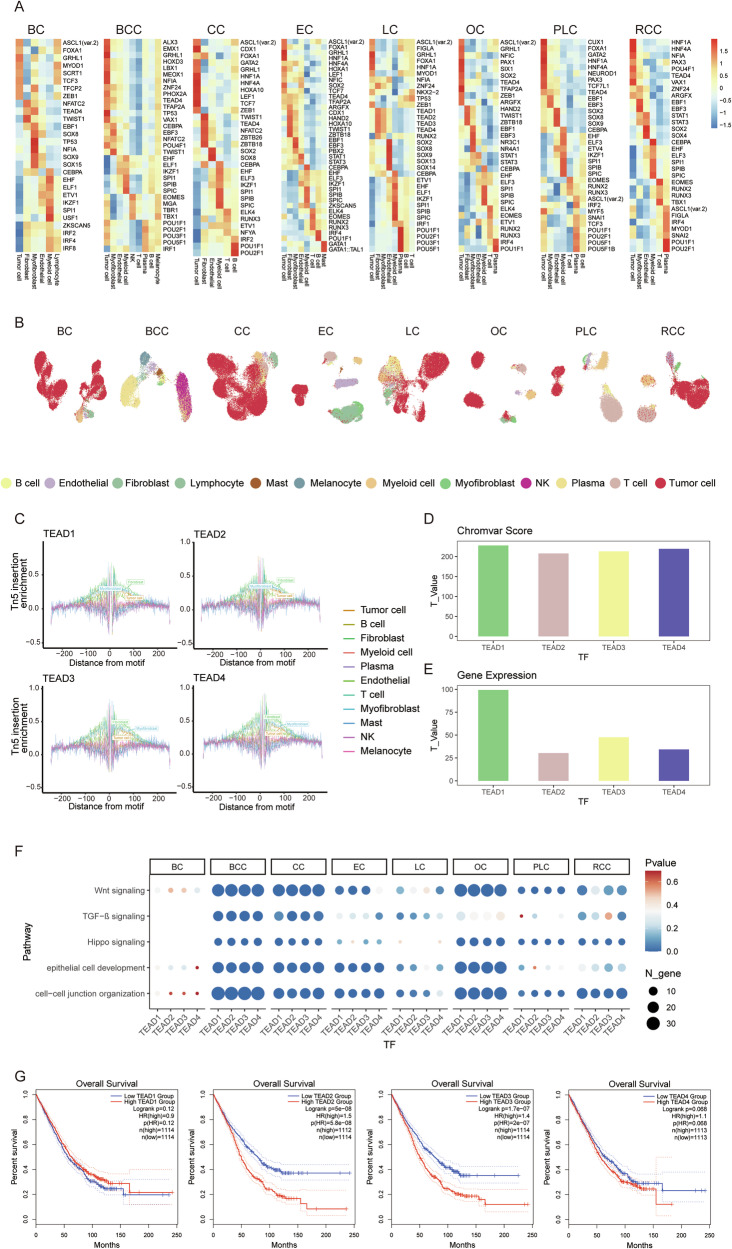
Transcription factor activities in different cell types in the eight different carcinomas. **A** Heatmap illustrating chromVAR bias-corrected deviation scores for the differential TF motifs of various cell types in eight different carcinomas. **B** UMAP visualization of single cells based on chromVAR bias-corrected deviations for identified motifs. **C** TF footprinting of TEAD TFs in diverse cell types in the integrated scATAC-seq data. **D** Bar plot of TEAD TFs analyzed by comparing the chromVAR score of tumor cells with other cell types. **E** Bar plot of TEAD TFs analyzed by comparing the gene expression of tumor cells with other cell types. **F** Dot plot of TEAD TFs regulating cancer-associated pathways in the eight carcinomas. The size of the dot represents the number of TF regulatory genes, and the color intensity of the dot indicates the *p*-value. **G** Kaplan-Meier survival analysis of TCGA cancer (COAD, LIHC, LUAD, KIRC, OV, and UCEC) patients with high (top 50%) and low (bottom 50%) expression for the *TEAD1/2/3/4* gene.

In prior studies, which elucidated the role of small sets of TFs in governing gene expression programs in tumor cells [57, 58], we sought to identify pivotal TFs participating in oncogenic transcriptional programs. To achieve this, we implemented a filtering strategy to identify highly specific TFs enriched across different cell types in each cancer type. Initially, we calculated the differentially enriched motifs through chromVAR bias-corrected scores for all cell types, selecting those highly enriched across diverse cell types. Subsequently, utilizing footprint analysis, we assessed the binding status of TFs across the genome for different cell types. In each cell type, TFs with a higher degree of activity and binding than those in other cell types were identified as cell-type-associated TFs (Fig. 5A, Supplementary Table S7). Employing UMAP with bias-corrected chromVAR scores for identified cell-type-enriched motifs, we effectively clustered single cells into distinct cell types, illustrating the efficacy of our strategy in screening for cell-type-associated TFs (Fig. 5B).

We made an intriguing discovery that AP-1 TFs exhibit extensive binding across different cell types, with elevated activity in tumor cells, fibroblasts, myofibroblasts, and endothelial cells (Supplementary Fig. S6A, B). To evaluate the impact of AP-1 TFs on gene expression regulation, we mapped their binding sites along with those of 588 other TFs in pairs-linked regulatory regions. Our findings revealed that AP-1 TFs exhibit a more extensive binding pattern compared with other TFs, indicating their broader regulation of gene expression (Supplementary Fig. S6C).

Our investigations also revealed that the identified cell-type-associated TFs in multiple cancer types are similar. For instance, EOMES and TWIST1 are highly activated in T cells and fibroblasts, respectively, indicating a high degree of reliability and consistency. We further performed GO enrichment analysis on the regulatory genes of EOMES in different carcinomas individually, based on the identified peak-gene link networks. The results show that EOMES is predominantly involved in the regulation of T cell activation and other immune-related pathways (Supplementary Fig. S7A). Similarly, TWIST1 is involved in regulating extracellular matrix organization and mesenchyme development, correlating with expected fibroblast functions (Supplementary Fig. S7B). However, distinct TF activity profiles emerged from analyses of different tumor cells. Notably, TEAD1/2/3/4 are activated in tumor cells across most cancer types, except for BC, displaying higher binding status and expression levels compared to other cells in the integrated scATAC-seq and scRNA-seq data (Fig. 5C–E). TEAD family TFs are critical regulators of various oncogenic pathways, including the Hippo signaling pathway, Wnt signaling pathway, and TGF-β signaling pathway. Our investigation revealed that TEADs significantly regulate these pathways in various tumor cells (Fig. 5F). They also participate in the regulation of conserved regulatory pathways in tumor cells, such as cell junction and epithelial cell development. Additionally, overexpression of *TEAD1/2/3/4* is significantly linked to poorer overall survival across six cancers (COAD, LUAD, LIHC, KIRC, OV, and UCEC) in TCGA data (Fig. 5G). Across all available TCGA datasets, TEAD family TFs consistently correlate with poorer outcomes, highlighting their consistent impact on different tumor cells and suggesting their potential as prognostic biomarkers (Supplementary Fig. S7C).

### Tumor-specific transcription factors regulate malignant transcriptional programs in colon cancer

**Fig. 6:**
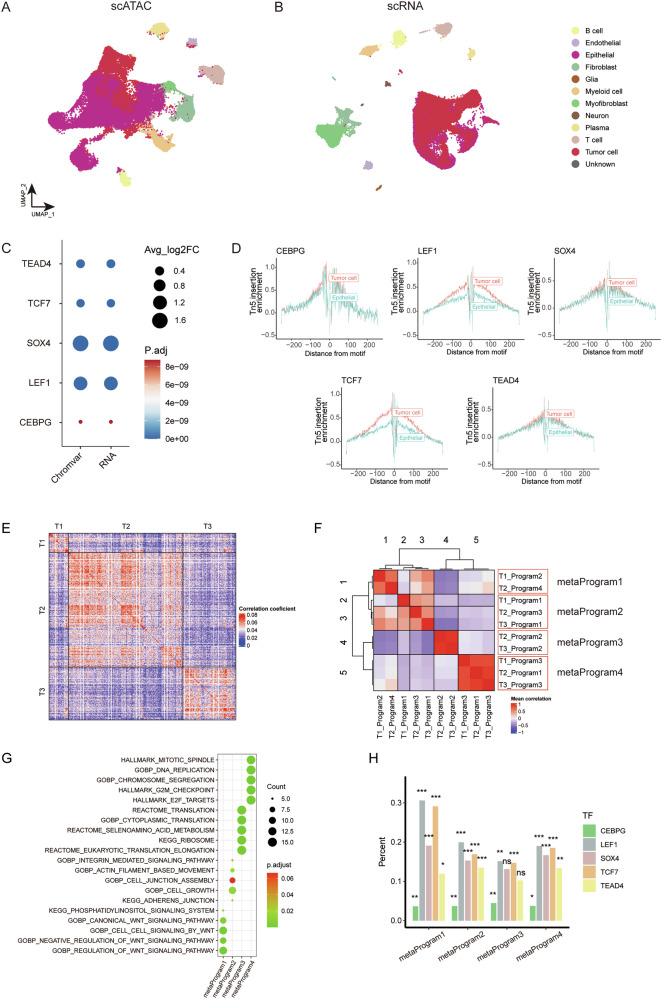
Tumor-specific TFs regulate malignant transcriptional programs in colon cancer. **A** Integrated UMAP projection based on scATAC-seq data, encompassing all cells from CC tissues and normal colon tissues. **B** Integrated UMAP projection based on scRNA-seq data, encompassing all cells from CC tissues and normal colon tissues. **C** Dot plot presenting changes of chromVAR bias-corrected deviation scores and gene expression between tumor cells and normal epithelial cells for the identified colon tumor-specific TF motifs. **D** TF footprinting of CEBPG, LEF1, SOX4, TCF7, and TEAD4 motifs in CC cells and normal colon epithelial cells. **E** Pairwise correlations between the expression profiles of three scRNA-seq CC samples. **F** Heatmap of average correlations across three samples with CC between pairs of programs. The red box indicates the four meta-programs. **G** Dot plot displaying significantly enriched pathways for signature genes of each meta-program. The dot size is proportional to the relative number of genes in the indicated pathway, and the color intensity indicates the *p*-value after FDR correction. **H** Proportion of the five identified tumor-specific TF motifs found in the regulatory elements for the signature genes for each of the four meta-programs (*p < 0.05, **p < 0.01, ***p < 0.001, ns: not significant).

We found that certain previously identified tumor cell-associated TFs are highly activated in normal epithelial. For instance, HNF4A exhibits high activity in colon and kidney epithelium [13, 59], while TFAP2C has been reported to play a crucial role in promoting epithelial gene expression [60]. To identify tumor-specific TFs, we conducted a comprehensive analysis of scATAC-seq and scRNA-seq data from normal colon tissues (Fig. 6A, B, Supplementary Fig. S8A, B). Five TFs (i.e. *CEBPG*, *LEF1*, *SOX4*, *TCF7*, and *TEAD4*) display higher chromVAR scores, increased binding, and elevated expression in tumor cells compared to normal epithelial cells (Fig. 6C, D, Supplementary Table S8).

To better understand the regulatory landscape driven by the five tumor-specific TFs (i.e. *CEBPG*, *LEF1*, *SOX4*, *TCF7*, and *TEAD4*) in CC cells, we utilized scRNA-seq data from CC tissues. We began with a pairwise correlation analysis, which revealed distinct transcriptional states consistently present within these tumors (Fig. 6E). Using nonnegative matrix factorization (NMF), we sought to elucidate the underlying transcriptional programs comprising co-expressed genes, and ultimately extracted 10 malignant transcriptional programs from the scRNA-seq data derived from three patients with CC (Supplementary Fig. S8C). Subsequent hierarchical clustering led to the identification of four meta-programs that encompassed highly similar programs across the three patient samples (Fig. 6F, Supplementary Fig. S8D-E, Table S9). Genes within meta-program 1 exhibit significant enrichment in the WNT signaling pathway, while meta-program 2 is predominantly enriched in pathways related to cell junction. Meta-program 3 and 4 showed enrichment in pathways associated with protein synthesis and DNA replication, respectively (Fig. 6G). To assess the impact of meta-programs on cellular viability in tumor cells, we evaluated the effect of loss-of-function of the top 100 genes in each meta-program on tumor cell viability, by examining CRISPR-Cas9 screening data from The Cancer Dependency Map Consortium (DepMap). Dependency scores (CERES) are normalized, with scores lower than 0 indicating essential genes for the given cell line. Notably, the mean CERES of most genes from 33 CC cell lines are lower than 0, suggesting they are essential in colon tumor cells (Supplementary Fig. S8F).

Next, we reviewed the top 100 genes within these four meta-programs and examined their linked peaks. Notably, motifs of the five tumor-specific TFs were significantly enriched in these peaks, indicating their key role in regulating gene expression in colon tumor cells (Fig. 6H). We further performed gene ontology enrichment analysis on the perturbagen’s signature of *CEBPG*, *LEF1*, *SOX4*, *TCF7*, and *TEAD4*, and the results reveal that the five tumor-specific TFs present extensive regulation on meta-program related pathways, consistent with the analysis before (Supplementary Fig. S8G).

We further investigated the regulatory interactions among *CEBPG*, *LEF1*, *SOX4*, *TCF7*, and *TEAD4* in colon cancer cells. Our constructed regulatory network in colon cancer revealed that *LEF1* is under the regulation of *SOX4* and *TCF7*, whereas *SOX4* is regulated by *LEF1*, *TCF7*, and *TEAD4*. Additionally, *CEBPG* is regulated by *LEF1*, *TEAD4*, and *SOX4*, while *TEAD4* is regulated by *LEF1* and *TCF7* (Supplementary Fig. S9A). These regulatory dynamics are further confirmed through TF perturbation experiments (Supplementary Fig. S9A, B). Analyzing the COAD RNA-seq data from TCGA, we observed strong correlations among the expressions of *LEF1*, *SOX4*, and *TCF7* (Supplementary Fig. S9C). These findings provide evidence of their regulatory relationships, indicating the presence of a transcriptional circuitry among tumor-specific TFs.

### The importance of tumor-specific TFs is widely supported

**Fig. 7:**
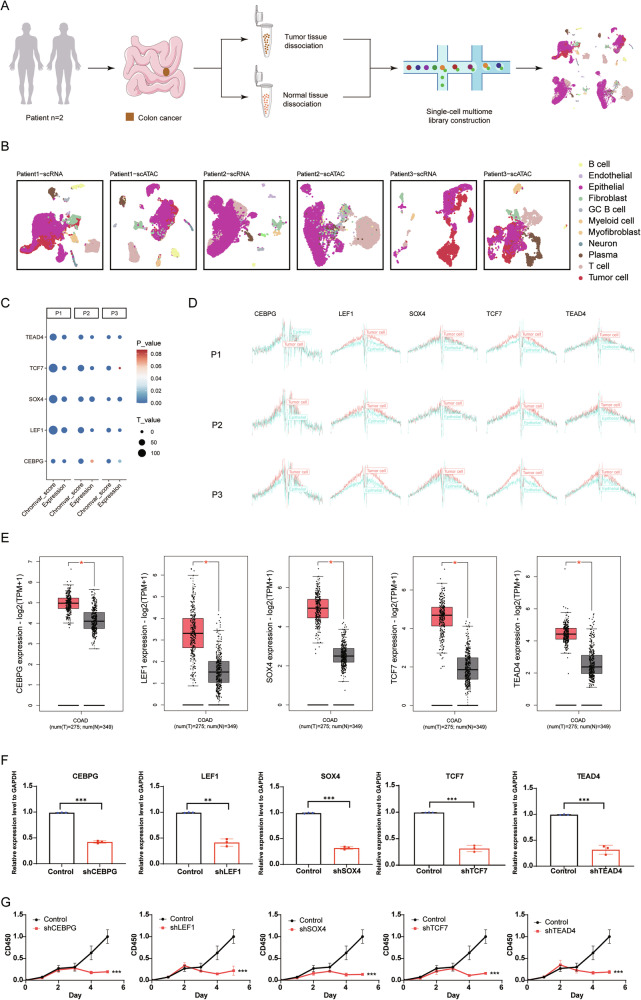
The importance of tumor-specific TFs is widely supported. **A** Schematic representation of the single-cell multiome sequencing experiments in this study. **B** Integrated UMAP projection based on scRNA-seq and scATAC-seq data, encompassing all cells from CC tissues and normal colon tissues of 3 patients. **C** Dot plot presenting changes of chromVAR bias-corrected deviation scores and expression levels for the identified colon tumor-specific TFs across tumor cells and normal epithelial cells. **D** TF footprinting of CEBPG, LEF1, SOX4, TCF7, and TEAD4 motifs in CC cells and normal colon epithelial cells for three patients. **E** Expression levels of the identified colon tumor-specific TFs in CC tissue samples (orange) and normal tissue samples (gray) obtained from the GEPIA2 database (*p < 0.05). **F** Downregulation of *CEBPG*, *LEF1*, *SOX4*, *TCF7*, and *TEAD4* mRNA expression in the DLD1 cell line by shRNA knockdown. Statistically significant differences are determined by two-way ANOVA (*p < 0.05, **p < 0.01, ***p < 0.001). **G** Effects of sh-CEBPG, sh-LEF1, sh-SOX4, sh-TCF7, sh-TEAD4, and vector (control) knockdown on DLD1 cell proliferation as determined by a cell proliferation assay. Statistically significant differences are determined by two-way ANOVA (*p < 0.05, **p < 0.01, ***p < 0.001).

To explore the significance of tumor-specific TFs, we performed single-cell multiome sequencing on paired CC tissues and adjacent normal tissues for two patients (“Patient 1” and “Patient 2”) (Fig. 7A, Supplementary Table S10). Additionally, we curated another dataset comprising scATAC-seq and scRNA-seq from the same patient, comparing tumor and normal samples (“Patient 3”). By integrating both transcriptomic and epigenetic data from the same patient samples, we minimized inter-patient variability (Fig. 7B, Supplementary Fig. S10A–D). Our analysis revealed that *CEBPG*, *LEF1*, *SOX4*, *TCF7*, and *TEAD4* exhibited higher expression levels and increased chromVAR scores in tumor cells compared to normal epithelial cells in the three patients (Fig. 7C). We also assessed their binding status across the entire open chromatin genome in tumor and epithelial cells, observing notably increased binding in tumor cells (Fig. 7D, Supplementary Table S8). Moreover, we integrated scATAC-seq data from eight colon tumor samples and seven normal colon samples, along with scRNA-seq data from twenty-three colon tumor samples and ten normal colon samples, which are collectively labeled as “Validation” (Supplementary Fig. S10E–H). The tumor-specific TFs with high activity in colon tumor cells, including *CEBPG*, *LEF1*, *SOX4*, *TCF7*, and *TEAD4*, exhibited elevated expression, higher chromVAR scores, and increased binding compared to normal epithelial cells (Supplementary Fig. S10I, J, Table S8). Further analysis using the TCGA and GTEx datasets confirmed that these TFs are expressed at higher levels in tumor tissue samples compared to normal tissue samples (Fig. 7E).

To further our understanding and validate the biological functions of these tumor-specific TFs, we carried out targeted knockdown experiments (Fig. 7F, Supplementary Fig. S11A). The results were striking, showing a substantial reduction in tumor cell proliferation by 5 days after TF knockdown (Fig. 7G). Moreover, migration was reduced, and apoptosis was induced in DLD1 cells with tumor-specific TF deletion, compared to control cells (Supplementary Fig. S11B, C). To identify potential drugs targeting these TFs, we took advantage of the LINCS consortium, which contains 19,811 small-molecule compound-perturbed profiles [47]. Through careful screening, we identified four pharmacological agents—tacedinaline, quinoclamine, dorsomorphin, and vorinostat—each targeting *CEBPG*, *LEF1*, *SOX4*, and *TEAD4*, respectively. Consistent with our expectations, these four compounds substantially attenuated both the expression levels of *CEBPG*, *LEF1*, *SOX4*, and *TEAD4*, and affected malignant phenotypes in the DLD1 cell lines, including proliferation, migration, and apoptosis (Supplementary Fig. S11D–H).

## Discussion

As an integrated approach, scATAC-seq and scRNA-seq data from diverse forms of carcinomas can provide a comprehensive analysis of chromatin accessibility and transcriptional characteristics within the tumor ecosystem. Using scATAC-seq data, we identified hundreds of thousands of cCREs in the cancer genome, effectively expanding the compendium of DNA regulatory elements compared to the bulk ATAC-seq data from TCGA. The robust overlap observed between methylation data and scATAC-seq data serves to underscore the reliability and consistency of our findings across platforms. These results provide a solid foundation for the subsequent exploration.

Using identified open chromatin regions limited to specific cell types, our investigations focus on discerning the potential regulatory roles of selected SNPs that are significantly associated with cancer. Currently, the scrutiny of SNPs within non-coding regions revolves predominantly around their gene regulatory functions within tumor cell lines [61–64]. Rather than just focusing on tumor cells, our single-cell ATAC-seq data provides a more comprehensive view, allowing us to identify which cell types exhibit heightened accessibility to these cancer-risk SNPs. This insight enables a more precise understanding of the specific cellular functions regulated by these SNPs, shedding light on their roles in cancer initiation and progression. Our analysis also revealed significant enrichment of GWAS SNPs associated with cancer in fibroblasts and myofibroblasts, as shown by our cell-type-specific LDSC analysis. These findings underscore the importance of studying the regulatory functions of SNPs within these two stromal cell types, opening up new avenues for research and emphasizing the importance of considering the diverse cellular contexts in which cancer-risk SNPs exert their regulatory effects.

Identifying the target genes of cCREs is important to reveal the cancer gene regulatory network [3]. To ensure the network’s reliability, we adopted the method established by Granja et al. [41] to assess the correlations between peaks and target genes. In parallel, Cicero analysis was performed to calculate the co-accessibility scores between peaks and the target gene promoters, which helped to identify more robust peak-gene links. Previous studies have employed similar strategies [65, 66]; however, their accessibility-expression correlation is calculated across cell types or samples, which may limit the scope of conclusions due to sample size constraints and cell type diversity. Overall, we constructed a peak-gene link network by integrated analysis of scATAC-seq data and scRNA-seq data, offering a more comprehensive understanding of the genome-wide regulatory landscape of gene expression. This integrated analysis not only provides deeper insights into differential cancer gene regulation but also highlights the regulatory roles of genetic risk SNPs. Previous studies have discovered that cancer epigenetic regulation is heterogeneous [67, 68], highlighting the importance of identifying conserved regulatory elements in specific cell types. Our analysis identifies conserved regulatory elements in tumor cells, T cells, myeloid cells, fibroblasts, myofibroblasts, and endothelial cells, revealing the potential functions they regulate and demonstrating inherent regulatory patterns across different carcinomas.

Previous reports have profiled TFs across various cancers; for instance, Jessica et al. predicted master TFs in cancers based on RNA-seq data [58], while Nadezhda et al. focused on altered TFs during cancer transitions using expression data from scRNA-seq [12]. However, our analysis focuses on identifying TFs activated in specific cell types within the tumor ecosystem using scATAC-seq data. This approach reveals regulons involved in cancer transcriptional programs and offers novel insights for identifying potential therapeutic targets. By analyzing tumor-cell highly activated TFs, we found that TEAD family TFs exhibit elevated activity in tumor cells across most carcinomas, which are reported as important regulators of tumor-associated pathways [69, 70] and widely regarded as targets for cancer treatment [71]. We also noted that their increased expression is associated with a poor prognosis, indicating their potential as biomarkers and demonstrating the effectiveness of our scATAC-seq approach. Our focused investigations identified tumor-specific TFs in CC, by strategically excluding those TFs highly activated in normal epithelial cells from which the tumor originates. These tumor-specific TFs appear to present potential functions to regulate each other and play an important role in governing malignant transcriptional programs, acting like master TFs [72]. Through bioinformatic analyses and experiment validation, these TFs may be potential targets for therapeutic interventions. For example, *TCF7* and *LEF1*, key mediators of WNT signaling, emerged as major regulators in our study, consistent with their well-established role as drivers of CC [73, 74]. Consistent with our findings, drugs targeting *TCF7* and *LEF1* have been used in several studies to treat CC cells [75–77], with promising therapeutic responses. We also identified *CEBPG*, *SOX4* and *TEAD4*, which widely control the gene expression of CC cells, exhibiting a strong impact on the malignant phenotype of colon tumor cells. While *CEBPG* has been extensively studied in myeloid and lymphoid leukemia [78, 79], our findings revealed its key role in CC, but the underlying mechanisms remain to be investigated in future studies. Our strategy for identifying tumor-specific TFs is broadly applicable and can help to provide therapeutic targets for more epithelial cancers.

It is crucial to acknowledge the limitations of our study to prevent unwarranted overinterpretation. Firstly, the sample size of our cancer datasets is constrained. While we identified a substantial number of cCREs compared to bulk ATAC-seq, augmenting the size of our dataset would enhance our ability to capture additional cCREs, affording a more comprehensive portrayal of the cellular heterogeneity within the tumor ecosystem and facilitating a more nuanced exploration of differential gene regulation. Secondly, our analyses of normal tissues are limited to colon samples, and thus the identification of tumor-specific TFs for BC, BCC, EC, LC, OC, PLC, and RCC necessitates additional studies. Lastly, our insights into gene expression regulation stem from the predictive power of integrated scATAC-seq and scRNA-seq data. While these findings provide valuable resources, they require rigorous experimentation in future studies.

Although the molecular causes of cancer continue to elude our complete understanding, our investigation provides novel insights into the complex landscape of cancer-associated gene expression regulation. This is particularly noteworthy concerning genomic loci associated with well-defined heritable disease risks. Our analytical framework stands as a blueprint, offering a strategic approach for investigating complex traits by exploiting the power of single-cell multi-omics data. Ultimately, these and other studies will lead to improved design of rational therapeutic strategies.

## Supplementary information

SupplementaryMaterials

UncroppedWesternBlots

aj-checklist

## Data Availability

All the multi-omics processed data are available here: https://www.synapse.org/#!Synapse:syn53709111. The FASTQ files generated during the current study are available at the Genome Sequence Archive for Human (GSA-Human) (accession number HRA010758) under the corresponding project at the National Genomics Data Center (NGDC) database (accession number PRJCA037365).

## Code availability

The custom code used for this manuscript is available on GitHub: https://github.com/txw-lab/MultiomicCancers.
