# Supplementary material for: Single-cell multi-omics analysis reveals cancer regulatory elements of transcriptional programs and clinical implications
Source: Cell Death Dis. 2025 Oct 21;16(1):746. doi: 10.1038/s41419-025-08060-7 (PMC12541060; doi:10.1038/s41419-025-08060-7)
Supplement: Supplementary file 1 — SupplementaryMaterials [file 41419_2025_8060_MOESM1_ESM.pdf]

Supplementary Figures

Figure S1. Cell type annotation for scATAC-seq and scRNA-seq profiles

(A) Normalized chromatin accessibility profiles for each cell type at canonical marker genes.

(B) Dot plots illustrating the gene expression patterns of cell-type marker genes in scRNA-seq data.

(C) Heatmaps of CNV signals normalized against the non-tumor cells for CNV changes by chromosome (columns) within individual cells (rows). All cells in the tumor population exhibit chromosome loss (blue dotted frame) or chromosome gain (red dotted frame), which are classic genomic features of BC, BCC, CC, EC, LC, OC, PLC, and RCC.

Figure S2. Open chromatin regions in scATAC-seq data

(A) Distributions of the cell numbers of each peak observed.

(B) Heatmap of methylation quantiles across cancer types in peak regions (left) and non-peak regions (right). Color depicts the methylation beta value of each quantile.

(C) Bar plot showing the number of cell type-specific cancer-associated peaks and a line chart indicating the proportion of cell type-specific cancer-associated peaks.

(D) Circle plots showing the number of SNPs in the accessible regions (small) and inaccessible regions (large) obtained from BC, CC, EC, LC, OC, PLC, and RCC scATAC-seq dataset.

Figure S3. The relationship between marker genes of stromal cells and survival rates

(A) Gene expression patterns projected onto UMAP plot of marker genes for CC fibroblasts.

(B) Kaplan-Meier survival analysis of TCGA COAD patients with high (top 50%) and low (bottom 50%) expression for the marker genes of CC fibroblasts.

(C) Gene expression patterns projected onto UMAP plot of marker genes for OC fibroblasts and myofibroblasts.

(D) Kaplan-Meier survival analysis of TCGA OV patients with high (top 50%) and low (bottom 50%) expression for the marker genes of OC fibroblasts and myofibroblasts.

Figure S4. Open chromatin regions of *MYC* and the regulatory elements of *NDRG1*

(A) Violin plot of normalized gene expression for *MYC* in all cell types of integrated scRNA-seq data.

(B) Browser track showing the accessibility profile at the *MYC* locus in all cells of integrated scATAC-seq data.

(C) UMAP projection of tumor cells from all cancer types based on scATAC-seq data.

(D) Normalized scATAC-seq tracks of different tumor cells at the locus of *ERBB2*, *KRT14*, *KRT8*, *SCGB2A1*, *FOXA1*, *KRT6A*, *APOA2*, and *CA9*.

(E-G) Volcano plots of DARs in BC(E), LC(F) and PLC(G), and chr8-133320109-133321082 is down-regulated, down-regulated, and stable.

(H) The proportions of candidate regulatory regions relative to Top100, Top300, Top500, Top1000, Top1500, and Top2000 DARs for different cell types across 8 cancer types.

Figure S5. Cell-type-conserved epigenetic regulation

(A-B) Predicted regulatory architecture at GWAS loci in CC. Genomic coverage plots for diverse cell types are shown separately. Rashkin et al. CC GWAS statistics for each SNPs are shown. Lead SNPs are shown as diamonds.

(C) Gene Ontology enrichment analysis of predicted genes regulated by tumor cell-conserved regulatory elements.

(D) Distinct sets of pathways were identified by gene ontology enrichment analysis of tumor cell-associated regulatory genes in each tumor type. UpSet plot shows the overlap of pathways across the groups.

(E) GREAT enrichment analysis on conserved regulatory regions in T cell, myeloid cell, fibroblast, myofibroblast, and endothelial cell.

(F) Gene Ontology enrichment analysis on predicted genes that conserved regulatory regions regulate in T cell, myeloid cell, fibroblast, myofibroblast, and endothelial cell.

Figure S6. AP-1 TFs highly activated in different cell types

(A) Heatmap illustrating chromVAR bias-corrected deviation scores for the AP-1 TF motifs of various cell types in eight different carcinomas.

(B) TF footprinting of FOS and JUNB motifs in diverse cell types of eight carcinomas.

(C) Distributions of the predicted regulatory region numbers of AP-1 motifs and other human motifs observed.

Figure S7. Cell-type-associated TFs regulate different pathways and serve as biomarkers

(A) Gene Ontology enrichment analysis of EOMES regulatory genes in BCC, CC, EC, LC, OC, PLC, and RCC.

(B) Gene Ontology enrichment analysis of TWIST1 regulatory genes in BC, CC, EC, LC, and OC.

(C) Kaplan-Meier survival analysis of TCGA pan-cancer patients with high (top 50%) and low (bottom 50%) expression for the *TEAD1/2/3/4* gene.

Figure S8. Tumor-specific TFs regulate gene expression of tumor cells

(A) Normalized chromatin accessibility profiles for canonical marker genes for various cell types.

(B) Dot plot showing the gene expression patterns of cell-type marker genes in scRNA-seq data derived from colon tumor tissues and normal colon tissues.

(C) Line plots showing cophenetic measure changes in each sample individually. The abscissa corresponding to the red dot on each plot indicates the optimal k value for NMF.

(D) Dot plots showing the Pearson correlation between pairs of programs in each sample individually.

(E) UMAP of tumor cells was captured from the scRNA-seq data, colored by the patient (left) and meta-program information (right).

(F) Bar plot of top 100 genes in each meta-program. Essential genes are colored in gray, and non-essential genes are colored in green.

(G) Dot plot of tumor-specific TFs regulating meta-program associated pathways. The size of the dot represents the number of perturbagen's signature of *CEBPG*, *TCF7*, *LEF1*, *SOX4*, and *TEAD4*, and the color intensity of the dot indicates the Bonferroni adjusted p-value.

Figure S9. Tumor-specific TFs regulate each other

(A) Regulatory relationships among *CEBPG*, *LEF1*, *SOX4*, *TEAD4*, and *TCF7*. Blue lines indicate relationships predicted by the constructed regulatory network, and red lines indicate relationships observed from the connectivity map.

(B) Perturbagen's signature of *TCF7*, *LEF1*, *SOX4*, and *TEAD4*. Red dots and green dots represent that their perturbagen's signature were identified by knockdown and over expression, respectively.

(C) The correlation plots of gene expressions between *LEF1*, *SOX4*, and *TCF7* based on COAD datasets from TCGA.

Figure S10. The importance of tumor-specific TFs

(A) Dot plot showing the gene expression patterns of cell-type marker genes in scRNA-seq data derived from colon tumor tissues and normal colon tissues of patient 1.

(B) Dot plot showing the gene expression patterns of cell-type marker genes in scRNA-seq data derived from colon tumor tissues and normal colon tissues of patient 2.

(C) Normalized chromatin accessibility profiles for canonical marker genes for various cell types from colon tumor tissues and normal colon tissues of patient 3.

(D) Dot plot showing the gene expression patterns of cell-type marker genes in scRNA-seq data derived from colon tumor tissues and normal colon tissues of patient 3.

(E) Integrated UMAP projection based on scATAC-seq data, encompassing all cells from validated CC tissues and normal colon tissues.

(F) Normalized chromatin accessibility profiles for canonical marker genes for various cell types from validated colon tumor tissues and normal colon tissues.

(G) Integrated UMAP projection based on scRNA-seq data, encompassing all cells from validated CC tissues and normal colon tissues.

(H) Dot plot showing the gene expression patterns of cell-type marker genes in scRNA-seq data derived from validated colon tumor tissues and normal colon tissues.

(I) Dot plot presenting changes of chromVAR bias-corrected deviation scores and expression levels for the identified colon tumor-specific TFs across tumor cells and normal epithelial cells in validated data.

(J) TF footprinting of CEBPG, LEF1, SOX4, TCF7, and TEAD4 motifs in CC cells and normal colon epithelial cells for validated data.

Figure S11. Tumor biology experiments in DLD1 cells with downregulation of tumor-specific TFs by shRNA and drug treatment

(A) Downregulation of CEBPG, LEF1, SOX4, TCF7, and TEAD4 protein expression in the DLD1 cell line by shRNA knockdown.

(B) Representative images and quantification of migrated cells after tumor-specific TFs knockdown in DLD1 cells. Scale Bar: 500 μm. Statistical significance is determined by the Student's t-test (*: $p < 0.05$, **: $p < 0.01$, ***: $p < 0.001$, ns: not significant).

(C) Representative flow cytometry profiles of apoptosis and quantitative results obtained using Annexin V/7-AAD staining after tumor-specific TFs knockdown in DLD1 cells. Q2 represents late apoptosis and Q3 represents early apoptosis. Statistical significance is determined by the Student's t-test (*: $p < 0.05$, **: $p < 0.01$, ***: $p < 0.001$, ns: not significant).

(D) The mRNA levels of *CEBPG*, *LEF1*, *SOX4*, and *TEAD4* after treatment with DMSO (control), tacedinaline, quinoclamine, dorsomorphin, or vorinostat in the DLD1 cell line were measured using qPCR. Statistical significance is determined by the Student's t-test (*: $p < 0.05$, **: $p < 0.01$, ***: $p < 0.001$, ns: not significant).

(E) The protein expression levels of CEBPG, LEF1, SOX4, and TEAD4 after treatment with DMSO (control), tacedinaline, quinoclamine, dorsomorphin, or vorinostat in the DLD1 cell line.

(F) Cell proliferation was assessed over a 2-day time period after exposure of the indicated knockdown DLD1 cell lines to DMSO (control), quinoclamine, dorsomorphin, or vorinostat at the concentrations indicated. Statistical significance is determined by multiple t-tests (*: $p < 0.05$, **: $p < 0.01$, ***: $p < 0.001$, ns: not significant).

(G) Representative images and quantification of migrated cells after tumor-specific TFs knockdown in DLD1 cells. Scale Bar: 500 μm. Statistical significance is determined by the Student's t-test (*: $p < 0.05$, **: $p < 0.01$, ***: $p < 0.001$, ns: not significant).

(H) Representative flow cytometry profiles of apoptosis and quantitative results obtained using Annexin V/7-AAD staining after drug treatment in DLD1 cells. Q2 represents late apoptosis and Q3 represents early apoptosis. Statistical significance is determined by the Student's t-test (*: $p < 0.05$, **: $p < 0.01$, ***: $p < 0.001$, ns: not significant).

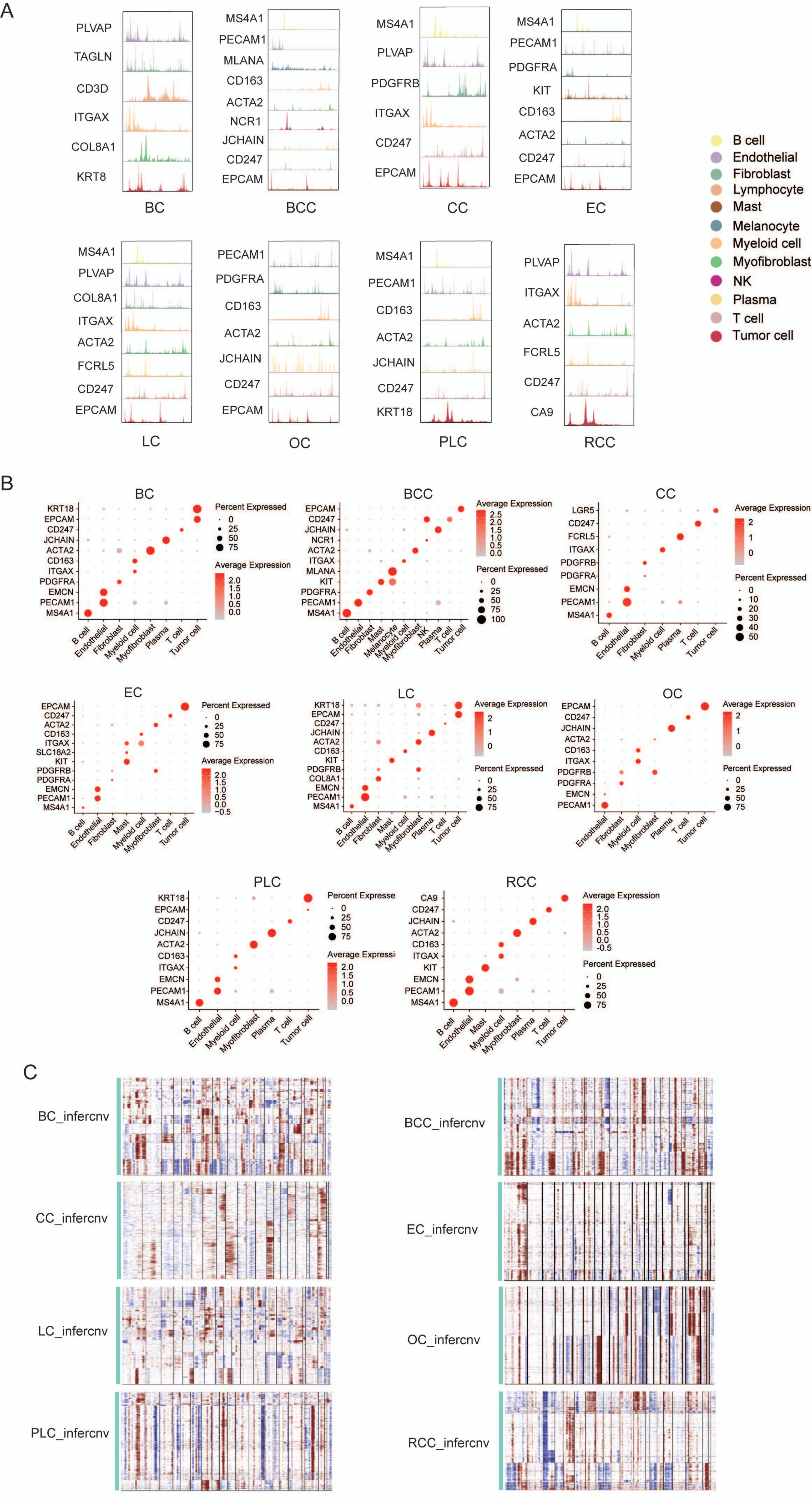
A
BC
BCC
CC
EC
LC
OC
PLC
RCC
B cell
Endothelial
Fibroblast
Lymphocyte
Mast
Melanocyte
Myeloid cell
Myofibroblast
NK
Plasma
T cell
Tumor cell
B
Percent Expressed
Average Expression
C
BC_infercnv
BCC_infercnv
CC_infercnv
EC_infercnv
LC_infercnv
OC_infercnv
PLC_infercnv
RCC_infercnv

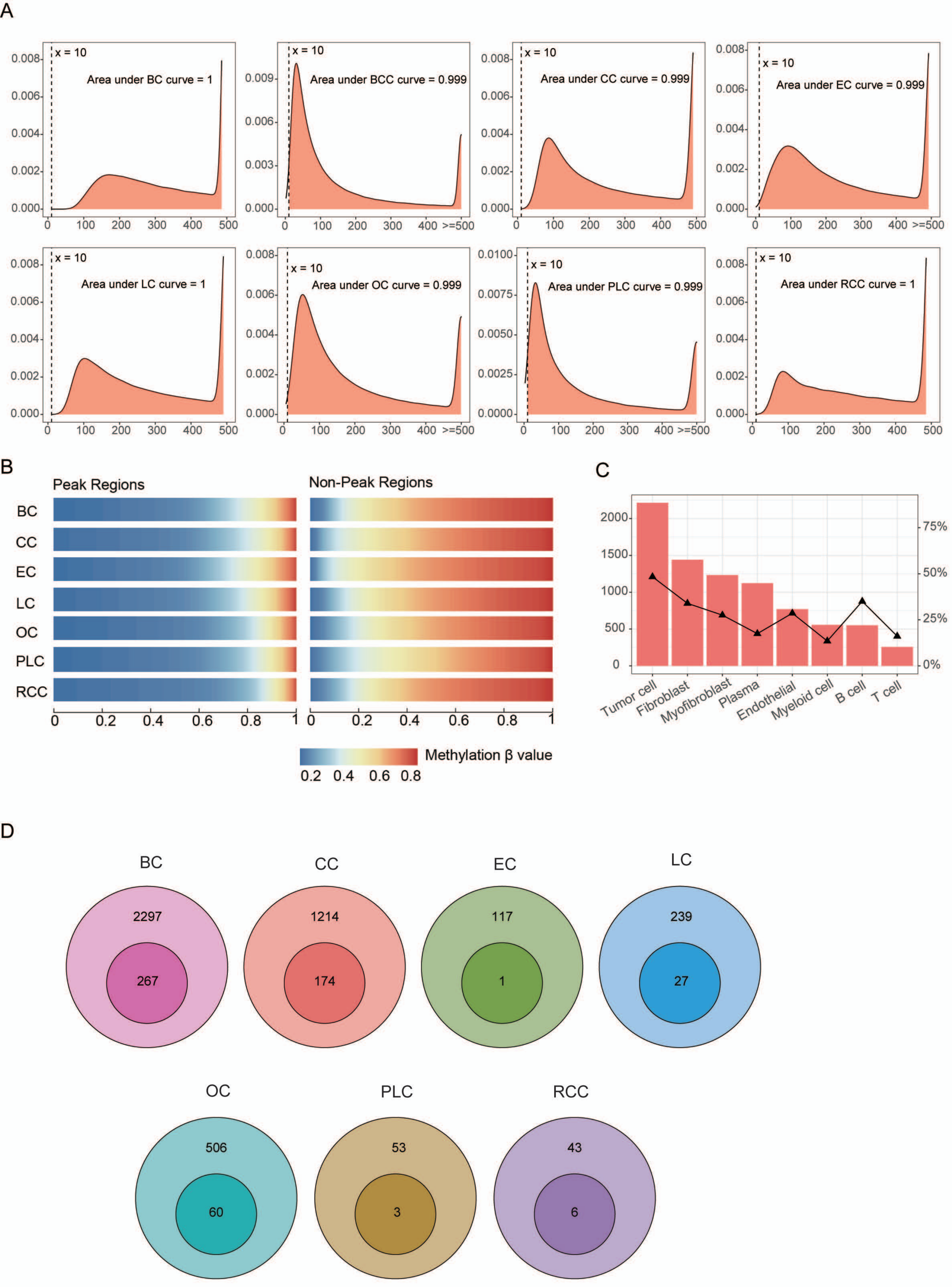

A
x = 10
Area under BC curve = 1
Area under BCC curve = 0.999
Area under CC curve = 0.999
Area under EC curve = 0.999
Area under LC curve = 1
Area under OC curve = 0.999
Area under PLC curve = 0.999
Area under RCC curve = 1
>=500
B
Peak Regions
Non-Peak Regions
BC
CC
EC
LC
OC
PLC
RCC
Methylation β value
C
Tumor cell
Fibroblast
Myofibroblast
Plasma
Endothelial
Myeloid cell
B cell
T cell
D
BC 2297 267
CC 1214 174
EC 117 1
LC 239 27
OC 506 60
PLC 53 3
RCC 43 6

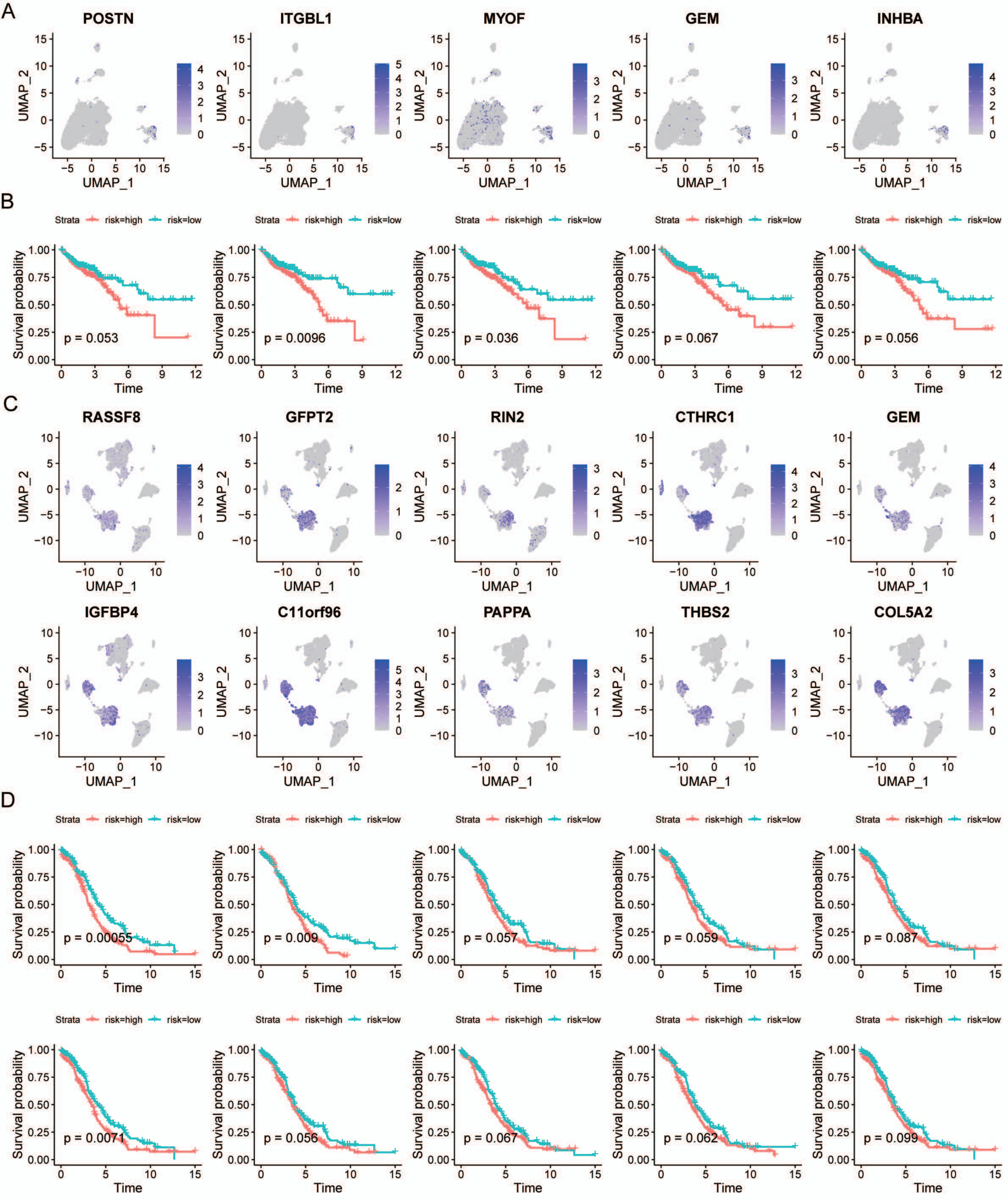
A
POSTN
ITGBL1
MYOF
GEM
INHBA
UMAP_2
UMAP_1
B
Strata risk=high risk=low
Survival probability
Time
p = 0.053
p = 0.0096
p = 0.036
p = 0.067
p = 0.056
C
RASSF8
GFPT2
RIN2
CTHRC1
GEM
IGFBP4
C11orf96
PAPPA
THBS2
COL5A2
UMAP_2
UMAP_1
D
Strata risk=high risk=low
Survival probability
Time
p = 0.00055
p = 0.009
p = 0.057
p = 0.059
p = 0.087
p = 0.0071
p = 0.056
p = 0.067
p = 0.062
p = 0.099

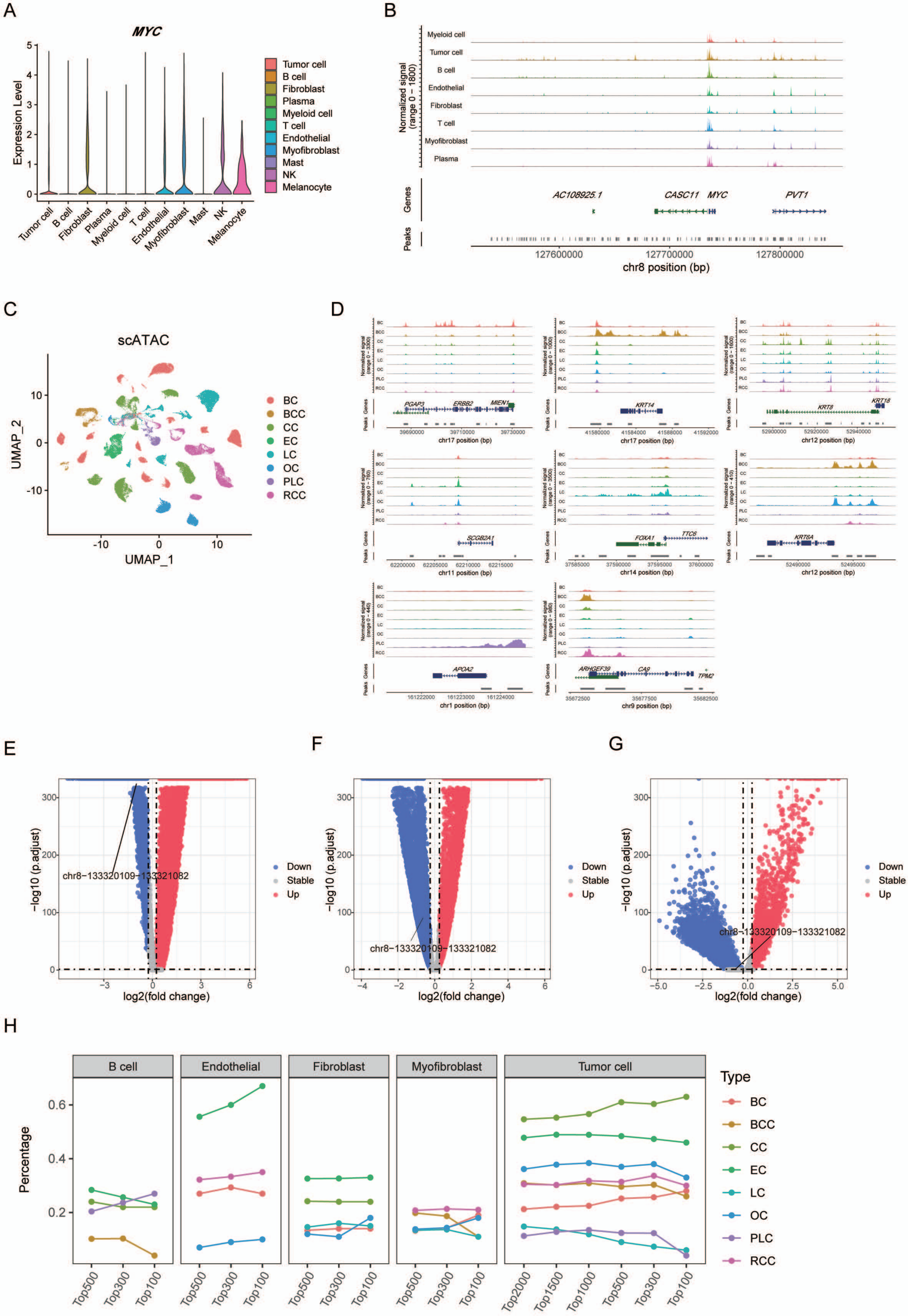

A
MYC
Expression Level
Tumor cell
B cell
Fibroblast
Plasma
Myeloid cell
T cell
Endothelial
Myofibroblast
Mast
NK
Melanocyte
B
Normalized signal (range 0 - 1800)
Myeloid cell
Tumor cell
B cell
Endothelial
Fibroblast
T cell
Myofibroblast
Plasma
Genes
AC108925.1
CASC11
MYC
PVT1
Peaks
127600000
127700000
127800000
chr8 position (bp)
C
scATAC
UMAP_2
UMAP_1
BC
BCC
CC
EC
LC
OC
PLC
RCC
D
PGAP3
ERBB2
MIEN1
chr17 position (bp)
KRT14
chr17 position (bp)
KRT8
KRT18
chr12 position (bp)
SCGB2A1
chr11 position (bp)
FOXA1
TTC6
chr14 position (bp)
KRT6A
chr12 position (bp)
APOA2
chr1 position (bp)
ARHGEF39
CA9
TPM2
chr9 position (bp)
E
-log10 (p.adjust)
log2(fold change)
chr8-133320109-133321082
Down
Stable
Up
F
-log10 (p.adjust)
log2(fold change)
chr8-133320109-133321082
Down
Stable
Up
G
-log10 (p.adjust)
log2(fold change)
chr8-133320109-133321082
Down
Stable
Up
H
Percentage
B cell
Endothelial
Fibroblast
Myofibroblast
Tumor cell
Top2000
Top1500
Top1000
Top500
Top300
Top100
Type
BC
BCC
CC
EC
LC
OC
PLC
RCC

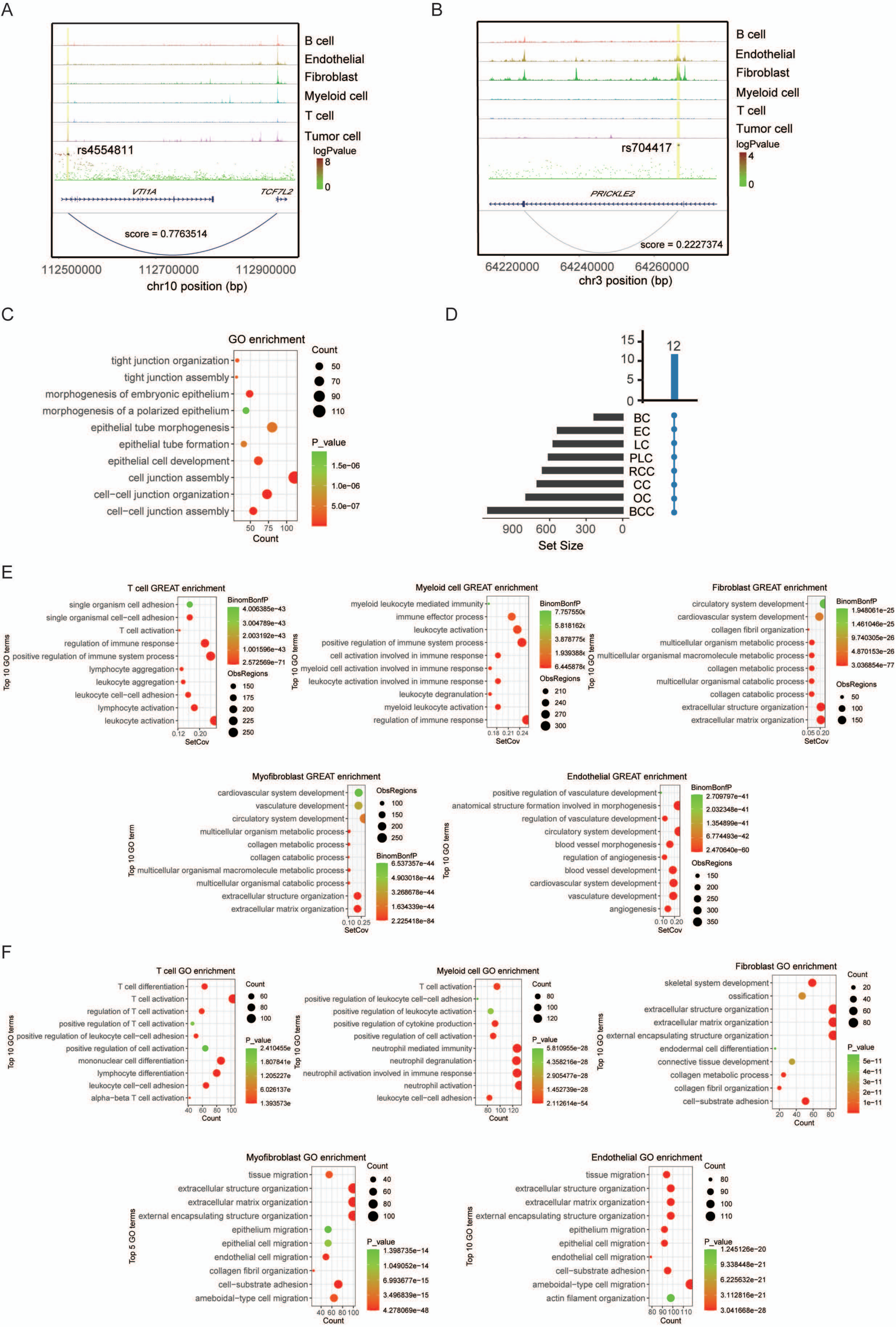

A
B cell
Endothelial
Fibroblast
Myeloid cell
T cell
Tumor cell
rs4554811
logPvalue
8
0
VTI1A
TCF7L2
score = 0.7763514
112500000
112700000
112900000
chr10 position (bp)
B
B cell
Endothelial
Fibroblast
Myeloid cell
T cell
Tumor cell
rs704417
logPvalue
4
0
PRICKLE2
score = 0.2227374
64220000
64240000
64260000
chr3 position (bp)
C
GO enrichment
tight junction organization
tight junction assembly
morphogenesis of embryonic epithelium
morphogenesis of a polarized epithelium
epithelial tube morphogenesis
epithelial tube formation
epithelial cell development
cell junction assembly
cell−cell junction organization
cell−cell junction assembly
Count
50 75 100
Count
50
70
90
110
P_value
1.5e−06
1.0e−06
5.0e−07
D
15
10
5
0
12
BC
EC
LC
PLC
RCC
CC
OC
BCC
900 600 300 0
Set Size
E
T cell GREAT enrichment
single organism cell adhesion
single organismal cell−cell adhesion
T cell activation
regulation of immune response
positive regulation of immune system process
lymphocyte aggregation
leukocyte aggregation
leukocyte cell−cell adhesion
lymphocyte activation
leukocyte activation
Top 10 GO terms
0.12 0.20
SetCov
BinomBonfP
4.006385e−43
3.004789e−43
2.003192e−43
1.001596e−43
2.572569e−71
ObsRegions
150
175
200
225
250
Myeloid cell GREAT enrichment
myeloid leukocyte mediated immunity
immune effector process
leukocyte activation
positive regulation of immune system process
cell activation involved in immune response
myeloid cell activation involved in immune response
leukocyte activation involved in immune response
leukocyte degranulation
myeloid leukocyte activation
regulation of immune response
Top 10 GO terms
0.18 0.21 0.24
SetCov
BinomBonfP
7.757550e
5.818162e
3.878775e
1.939388e
6.445878e
ObsRegions
210
240
270
300
Fibroblast GREAT enrichment
circulatory system development
cardiovascular system development
collagen fibril organization
multicellular organism metabolic process
multicellular organismal macromolecule metabolic process
collagen metabolic process
multicellular organismal catabolic process
collagen catabolic process
extracellular structure organization
extracellular matrix organization
Top 10 GO terms
0.05 0.20
SetCov
BinomBonfP
1.948061e−25
1.461046e−25
9.740305e−26
4.870153e−26
3.036854e−77
ObsRegions
50
100
150
Myofibroblast GREAT enrichment
cardiovascular system development
vasculature development
circulatory system development
multicellular organism metabolic process
collagen metabolic process
collagen catabolic process
multicellular organismal macromolecule metabolic process
multicellular organismal catabolic process
extracellular structure organization
extracellular matrix organization
Top 10 GO term
0.10 0.25
SetCov
ObsRegions
100
150
200
250
BinomBonfP
6.537357e−44
4.903018e−44
3.268678e−44
1.634339e−44
2.225418e−84
Endothelial GREAT enrichment
positive regulation of vasculature development
anatomical structure formation involved in morphogenesis
regulation of vasculature development
circulatory system development
blood vessel morphogenesis
regulation of angiogenesis
blood vessel development
cardiovascular system development
vasculature development
angiogenesis
Top 10 GO terms
0.10 0.20
SetCov
BinomBonfP
2.709797e−41
2.032348e−41
1.354899e−41
6.774493e−42
2.470640e−60
ObsRegions
150
200
250
300
350
F
T cell GO enrichment
T cell differentiation
T cell activation
regulation of T cell activation
positive regulation of T cell activation
positive regulation of leukocyte cell−cell adhesion
positive regulation of cell activation
mononuclear cell differentiation
lymphocyte differentiation
leukocyte cell−cell adhesion
alpha−beta T cell activation
Top 10 GO terms
40 60 80 100
Count
Count
60
80
100
P_value
2.410455e
1.807841e
1.205227e
6.026137e
1.393573e
Myeloid cell GO enrichment
T cell activation
positive regulation of leukocyte cell−cell adhesion
positive regulation of leukocyte activation
positive regulation of cytokine production
positive regulation of cell activation
neutrophil mediated immunity
neutrophil degranulation
neutrophil activation involved in immune response
neutrophil activation
leukocyte cell−cell adhesion
Top 10 GO terms
80 100 120
Count
Count
80
100
120
P_value
5.810955e−28
4.358216e−28
2.905477e−28
1.452739e−28
2.112614e−54
Fibroblast GO enrichment
skeletal system development
ossification
extracellular structure organization
extracellular matrix organization
external encapsulating structure organization
endodermal cell differentiation
connective tissue development
collagen metabolic process
collagen fibril organization
cell−substrate adhesion
Top 10 GO terms
20 40 60 80
Count
Count
20
40
60
80
P_value
5e−11
4e−11
3e−11
2e−11
1e−11
Myofibroblast GO enrichment
tissue migration
extracellular structure organization
extracellular matrix organization
external encapsulating structure organization
epithelium migration
epithelial cell migration
endothelial cell migration
collagen fibril organization
cell−substrate adhesion
ameboidal−type cell migration
Top 5 GO terms
40 60 80 100
Count
Count
40
60
80
100
P_value
1.398735e−14
1.049052e−14
6.993677e−15
3.496839e−15
4.278069e−48
Endothelial GO enrichment
tissue migration
extracellular structure organization
extracellular matrix organization
external encapsulating structure organization
epithelium migration
epithelial cell migration
endothelial cell migration
cell−substrate adhesion
ameboidal−type cell migration
actin filament organization
Top 10 GO terms
80 90 100 110
Count
Count
80
90
100
110
P_value
1.245126e−20
9.338448e−21
6.225632e−21
3.112816e−21
3.041668e−28

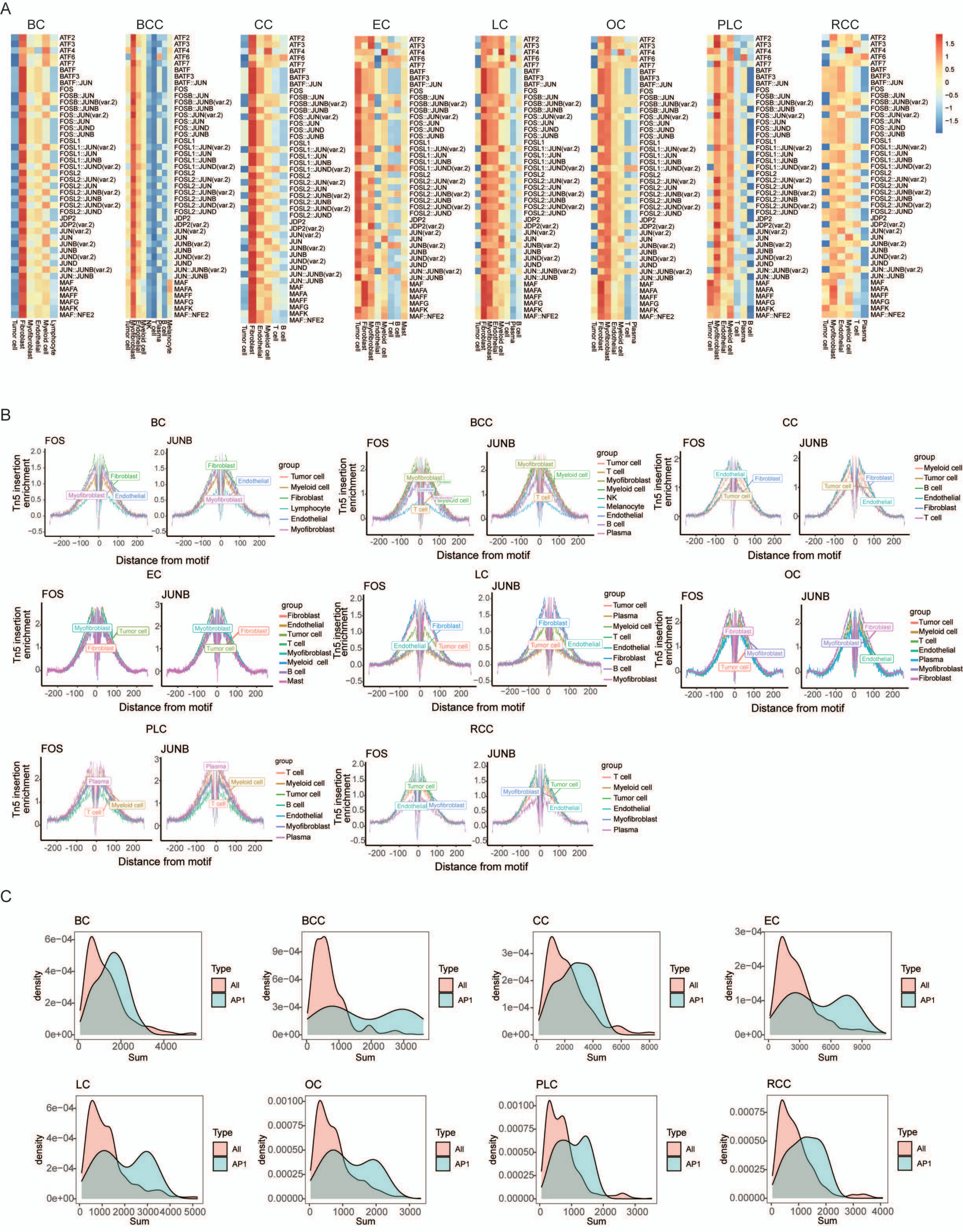
A
BC
BCC
CC
EC
LC
OC
PLC
RCC
ATF2
ATF3
ATF4
ATF6
ATF7
BATF
BATF3
BATF::JUN
FOS
FOSB::JUN
FOSB::JUNB(var.2)
FOSB::JUNB
FOS::JUN(var.2)
FOS::JUN
FOS::JUND
FOS::JUNB
FOSL1
FOSL1::JUN(var.2)
FOSL1::JUN
FOSL1::JUNB
FOSL1::JUND(var.2)
FOSL2
FOSL2::JUN(var.2)
FOSL2::JUN
FOSL2::JUNB(var.2)
FOSL2::JUNB
FOSL2::JUND(var.2)
FOSL2::JUND
JDP2
JDP2(var.2)
JUN(var.2)
JUN
JUNB(var.2)
JUNB
JUND(var.2)
JUND
JUN::JUNB(var.2)
JUN::JUNB
MAF
MAFA
MAFF
MAFG
MAFK
MAF::NFE2
Tumor cell
Fibroblast
Myofibroblast
Endothelial
Myeloid cell
Lymphocyte
NK
T cell
B cell
Plasma
Melanocyte
Mast
1.5
1
0.5
0
−0.5
−1
−1.5
B
FOS
JUNB
Tn5 insertion enrichment
Distance from motif
group
Tumor cell
Myeloid cell
Fibroblast
Lymphocyte
Endothelial
Myofibroblast
T cell
NK
Melanocyte
B cell
Plasma
Mast
C
density
Sum
Type
All
AP1

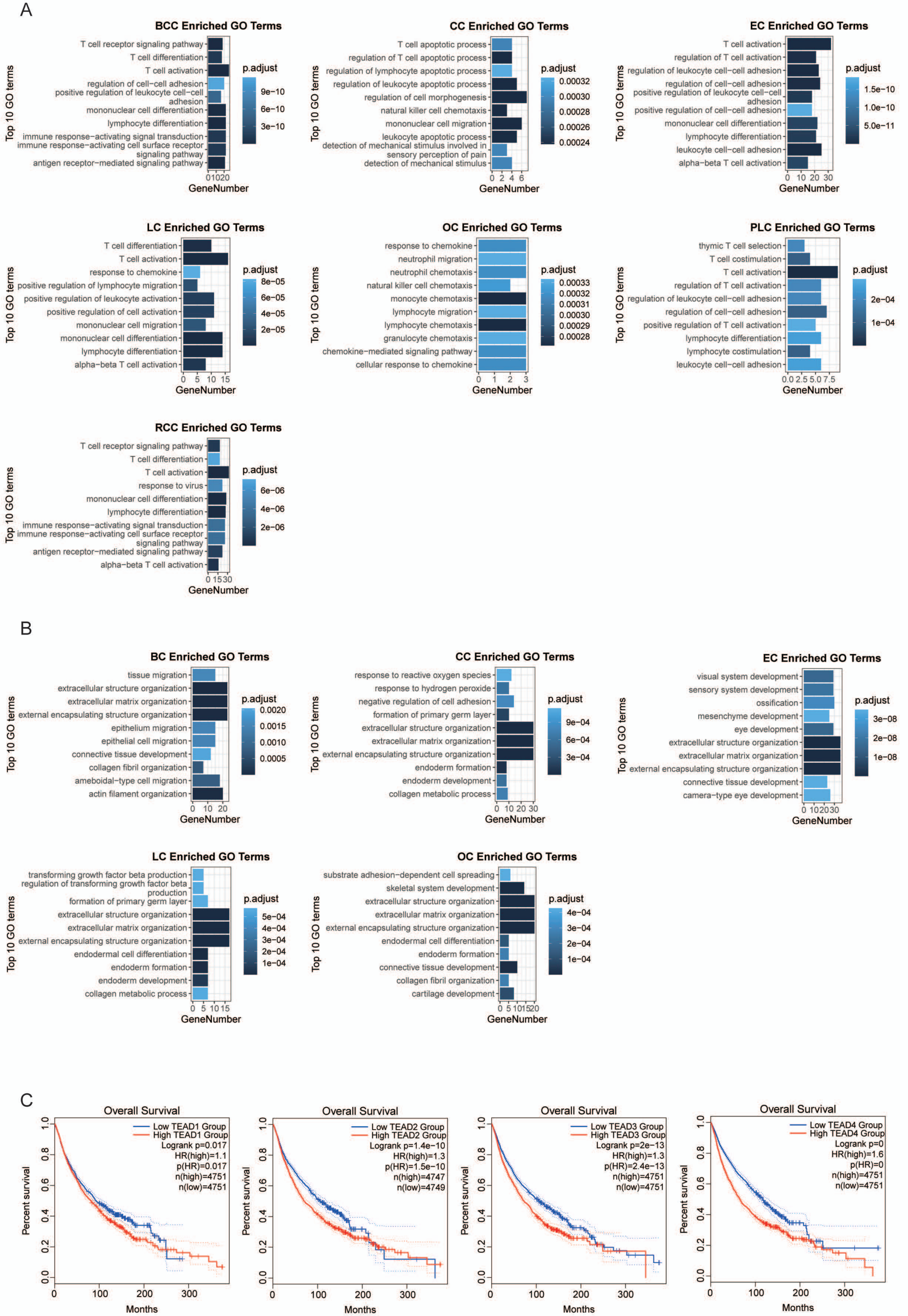
A
BCC Enriched GO Terms
T cell receptor signaling pathway
T cell differentiation
T cell activation
regulation of cell−cell adhesion
positive regulation of leukocyte cell−cell adhesion
mononuclear cell differentiation
lymphocyte differentiation
immune response−activating signal transduction
immune response−activating cell surface receptor signaling pathway
antigen receptor−mediated signaling pathway
Top 10 GO terms
GeneNumber
p.adjust
CC Enriched GO Terms
T cell apoptotic process
regulation of T cell apoptotic process
regulation of lymphocyte apoptotic process
regulation of leukocyte apoptotic process
regulation of cell morphogenesis
natural killer cell chemotaxis
mononuclear cell migration
leukocyte apoptotic process
detection of mechanical stimulus involved in sensory perception of pain
detection of mechanical stimulus
EC Enriched GO Terms
T cell activation
regulation of T cell activation
regulation of leukocyte cell−cell adhesion
regulation of cell−cell adhesion
positive regulation of leukocyte cell−cell adhesion
positive regulation of cell−cell adhesion
mononuclear cell differentiation
lymphocyte differentiation
leukocyte cell−cell adhesion
alpha−beta T cell activation
LC Enriched GO Terms
T cell differentiation
T cell activation
response to chemokine
positive regulation of lymphocyte migration
positive regulation of leukocyte activation
positive regulation of cell activation
mononuclear cell migration
mononuclear cell differentiation
lymphocyte differentiation
alpha−beta T cell activation
OC Enriched GO Terms
response to chemokine
neutrophil migration
neutrophil chemotaxis
natural killer cell chemotaxis
monocyte chemotaxis
lymphocyte migration
lymphocyte chemotaxis
granulocyte chemotaxis
chemokine−mediated signaling pathway
cellular response to chemokine
PLC Enriched GO Terms
thymic T cell selection
T cell costimulation
T cell activation
regulation of T cell activation
regulation of leukocyte cell−cell adhesion
regulation of cell−cell adhesion
positive regulation of T cell activation
lymphocyte differentiation
lymphocyte costimulation
leukocyte cell−cell adhesion
RCC Enriched GO Terms
T cell receptor signaling pathway
T cell differentiation
T cell activation
response to virus
mononuclear cell differentiation
lymphocyte differentiation
immune response−activating signal transduction
immune response−activating cell surface receptor signaling pathway
antigen receptor−mediated signaling pathway
alpha−beta T cell activation
B
BC Enriched GO Terms
tissue migration
extracellular structure organization
extracellular matrix organization
external encapsulating structure organization
epithelium migration
epithelial cell migration
connective tissue development
collagen fibril organization
ameboidal−type cell migration
actin filament organization
CC Enriched GO Terms
response to reactive oxygen species
response to hydrogen peroxide
negative regulation of cell adhesion
formation of primary germ layer
extracellular structure organization
extracellular matrix organization
external encapsulating structure organization
endoderm formation
endoderm development
collagen metabolic process
EC Enriched GO Terms
visual system development
sensory system development
ossification
mesenchyme development
eye development
extracellular structure organization
extracellular matrix organization
external encapsulating structure organization
connective tissue development
camera−type eye development
LC Enriched GO Terms
transforming growth factor beta production
regulation of transforming growth factor beta production
formation of primary germ layer
extracellular structure organization
extracellular matrix organization
external encapsulating structure organization
endodermal cell differentiation
endoderm formation
endoderm development
collagen metabolic process
OC Enriched GO Terms
substrate adhesion−dependent cell spreading
skeletal system development
extracellular structure organization
extracellular matrix organization
external encapsulating structure organization
endodermal cell differentiation
endoderm formation
connective tissue development
collagen fibril organization
cartilage development
C
Overall Survival
Low TEAD1 Group
High TEAD1 Group
Logrank p=0.017
HR(high)=1.1
p(HR)=0.017
n(high)=4751
n(low)=4751
Low TEAD2 Group
High TEAD2 Group
Logrank p=1.4e−10
HR(high)=1.3
p(HR)=1.5e−10
n(high)=4747
n(low)=4749
Low TEAD3 Group
High TEAD3 Group
Logrank p=2e−13
HR(high)=1.3
p(HR)=2.4e−13
n(high)=4751
n(low)=4751
Low TEAD4 Group
High TEAD4 Group
Logrank p=0
HR(high)=1.6
p(HR)=0
n(high)=4751
n(low)=4751
Percent survival
Months

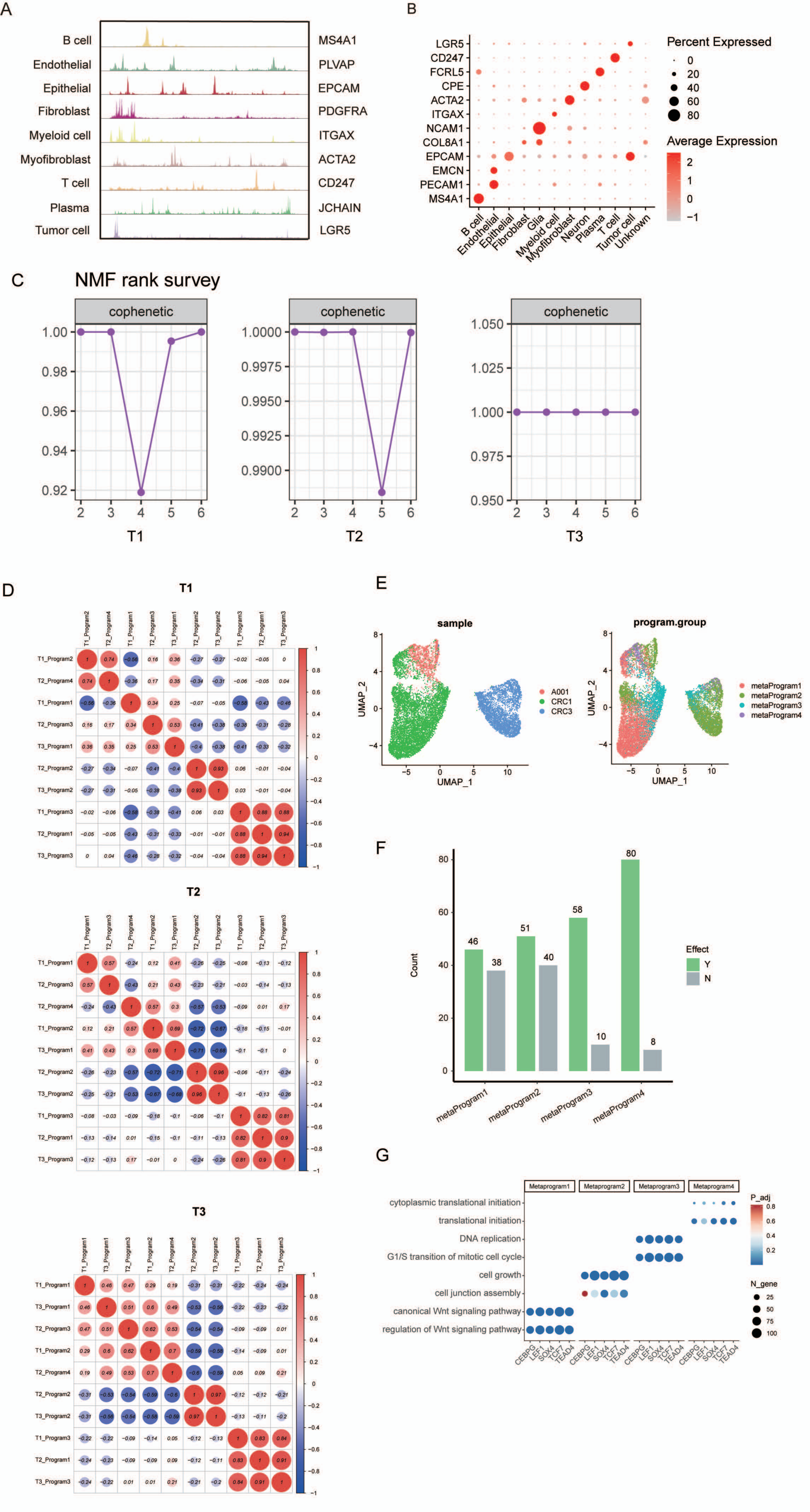
A
B cell
Endothelial
Epithelial
Fibroblast
Myeloid cell
Myofibroblast
T cell
Plasma
Tumor cell
MS4A1
PLVAP
EPCAM
PDGFRA
ITGAX
ACTA2
CD247
JCHAIN
LGR5
B
LGR5
CD247
FCRL5
CPE
ACTA2
ITGAX
NCAM1
COL8A1
EPCAM
EMCN
PECAM1
MS4A1
B cell
Endothelial
Epithelial
Fibroblast
Glia
Myeloid cell
Myofibroblast
Neuron
Plasma
T cell
Tumor cell
Unknown
Percent Expressed
0
20
40
60
80
Average Expression
2
1
0
−1
C
NMF rank survey
cophenetic
T1
T2
T3
D
T1
T2
T3
E
sample
program.group
UMAP_1
UMAP_2
A001
CRC1
CRC3
metaProgram1
metaProgram2
metaProgram3
metaProgram4
F
Count
Effect
Y
N
46
38
51
40
58
10
80
8
metaProgram1
metaProgram2
metaProgram3
metaProgram4
G
Metaprogram1
Metaprogram2
Metaprogram3
Metaprogram4
cytoplasmic translational initiation
translational initiation
DNA replication
G1/S transition of mitotic cell cycle
cell growth
cell junction assembly
canonical Wnt signaling pathway
regulation of Wnt signaling pathway
CEBPG
LEF1
SOX4
TCF7
TEAD4
P_adj
N_gene

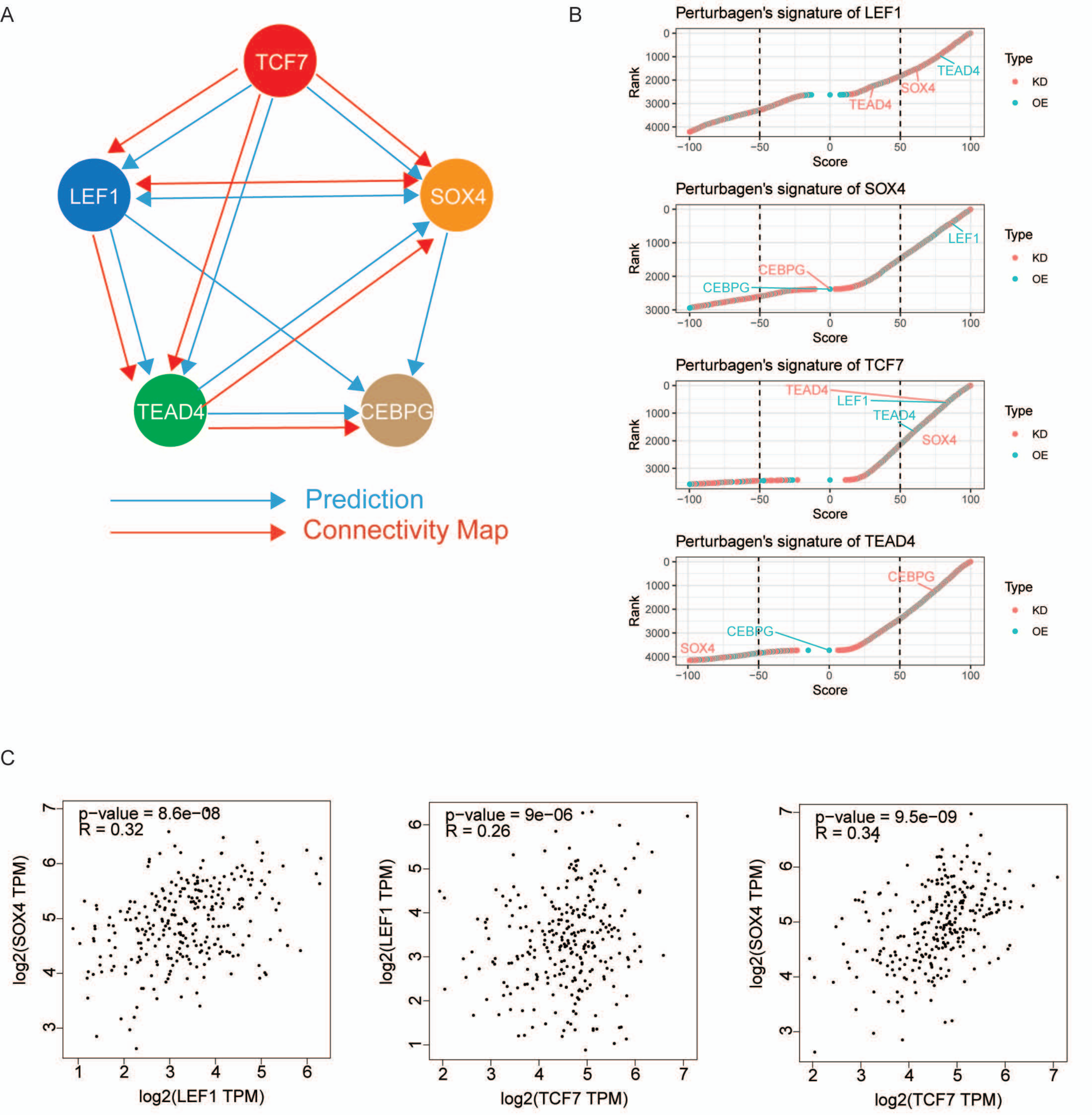

A
TCF7
LEF1
SOX4
TEAD4
CEBPG
Prediction
Connectivity Map
B
Perturbagen's signature of LEF1
Perturbagen's signature of SOX4
Perturbagen's signature of TCF7
Perturbagen's signature of TEAD4
Rank
Score
Type
KD
OE
C
p-value = 8.6e−08
R = 0.32
log2(SOX4 TPM)
log2(LEF1 TPM)
p-value = 9e−06
R = 0.26
log2(LEF1 TPM)
log2(TCF7 TPM)
p-value = 9.5e−09
R = 0.34
log2(SOX4 TPM)
log2(TCF7 TPM)

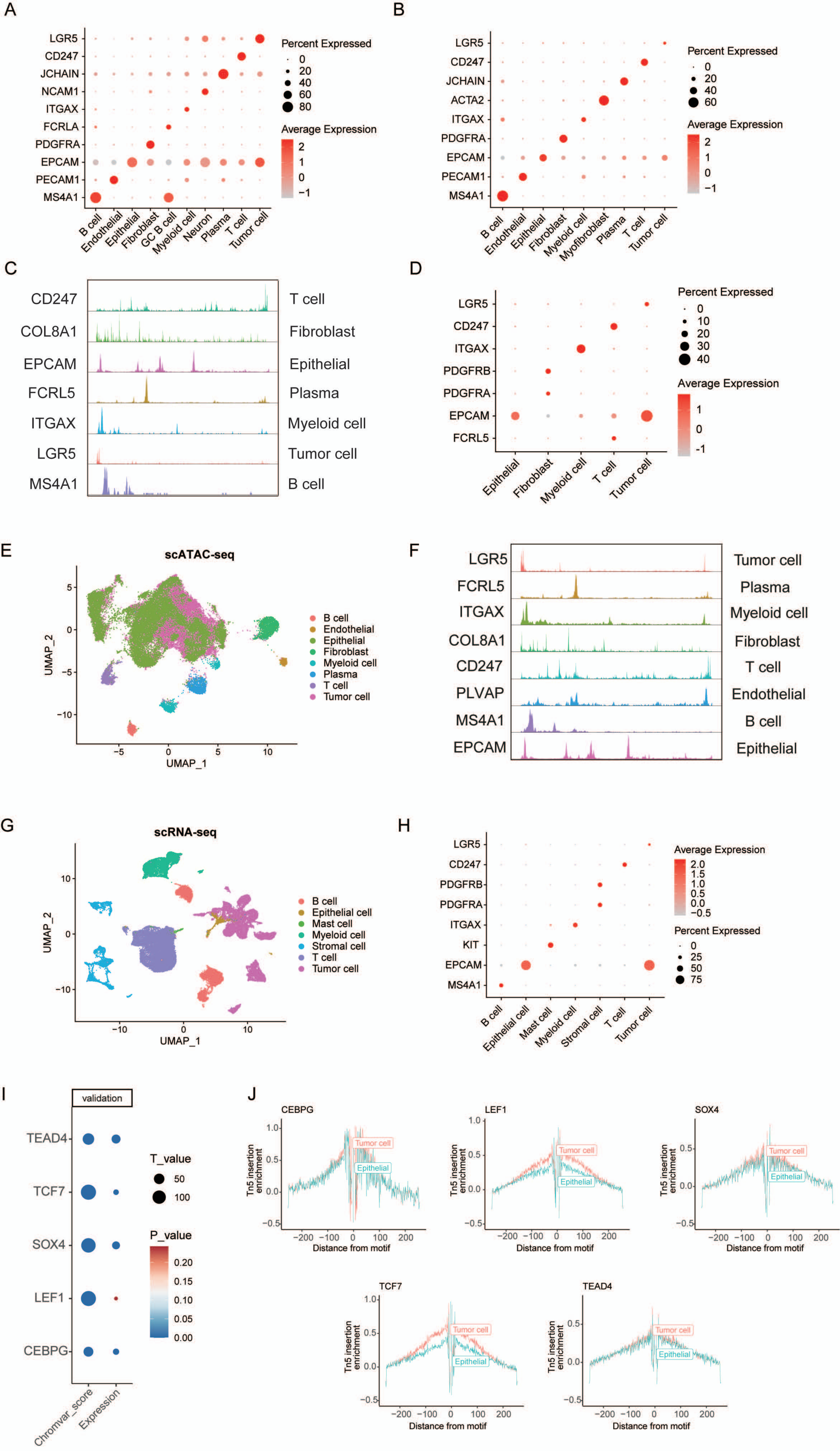
A
LGR5
CD247
JCHAIN
NCAM1
ITGAX
FCRLA
PDGFRA
EPCAM
PECAM1
MS4A1
B cell
Endothelial
Epithelial
Fibroblast
GC B cell
Myeloid cell
Neuron
Plasma
T cell
Tumor cell
Percent Expressed
0
20
40
60
80
Average Expression
2
1
0
−1
B
LGR5
CD247
JCHAIN
ACTA2
ITGAX
PDGFRA
EPCAM
PECAM1
MS4A1
B cell
Endothelial
Epithelial
Fibroblast
Myeloid cell
Myofibroblast
Plasma
T cell
Tumor cell
Percent Expressed
0
20
40
60
Average Expression
2
1
0
−1
C
CD247
COL8A1
EPCAM
FCRL5
ITGAX
LGR5
MS4A1
T cell
Fibroblast
Epithelial
Plasma
Myeloid cell
Tumor cell
B cell
D
LGR5
CD247
ITGAX
PDGFRB
PDGFRA
EPCAM
FCRL5
Epithelial
Fibroblast
Myeloid cell
T cell
Tumor cell
Percent Expressed
0
10
20
30
40
Average Expression
1
0
-1
E
scATAC-seq
UMAP_2
UMAP_1
B cell
Endothelial
Epithelial
Fibroblast
Myeloid cell
Plasma
T cell
Tumor cell
F
LGR5
FCRL5
ITGAX
COL8A1
CD247
PLVAP
MS4A1
EPCAM
Tumor cell
Plasma
Myeloid cell
Fibroblast
T cell
Endothelial
B cell
Epithelial
G
scRNA-seq
UMAP_2
UMAP_1
B cell
Epithelial cell
Mast cell
Myeloid cell
Stromal cell
T cell
Tumor cell
H
LGR5
CD247
PDGFRB
PDGFRA
ITGAX
KIT
EPCAM
MS4A1
B cell
Epithelial cell
Mast cell
Myeloid cell
Stromal cell
T cell
Tumor cell
Average Expression
2.0
1.5
1.0
0.5
0.0
−0.5
Percent Expressed
0
25
50
75
I
validation
TEAD4
TCF7
SOX4
LEF1
CEBPG
Chromvar_score
Expression
T_value
50
100
P_value
0.20
0.15
0.10
0.05
0.00
J
CEBPG
LEF1
SOX4
TCF7
TEAD4
Tn5 insertion enrichment
Distance from motif
Tumor cell
Epithelial

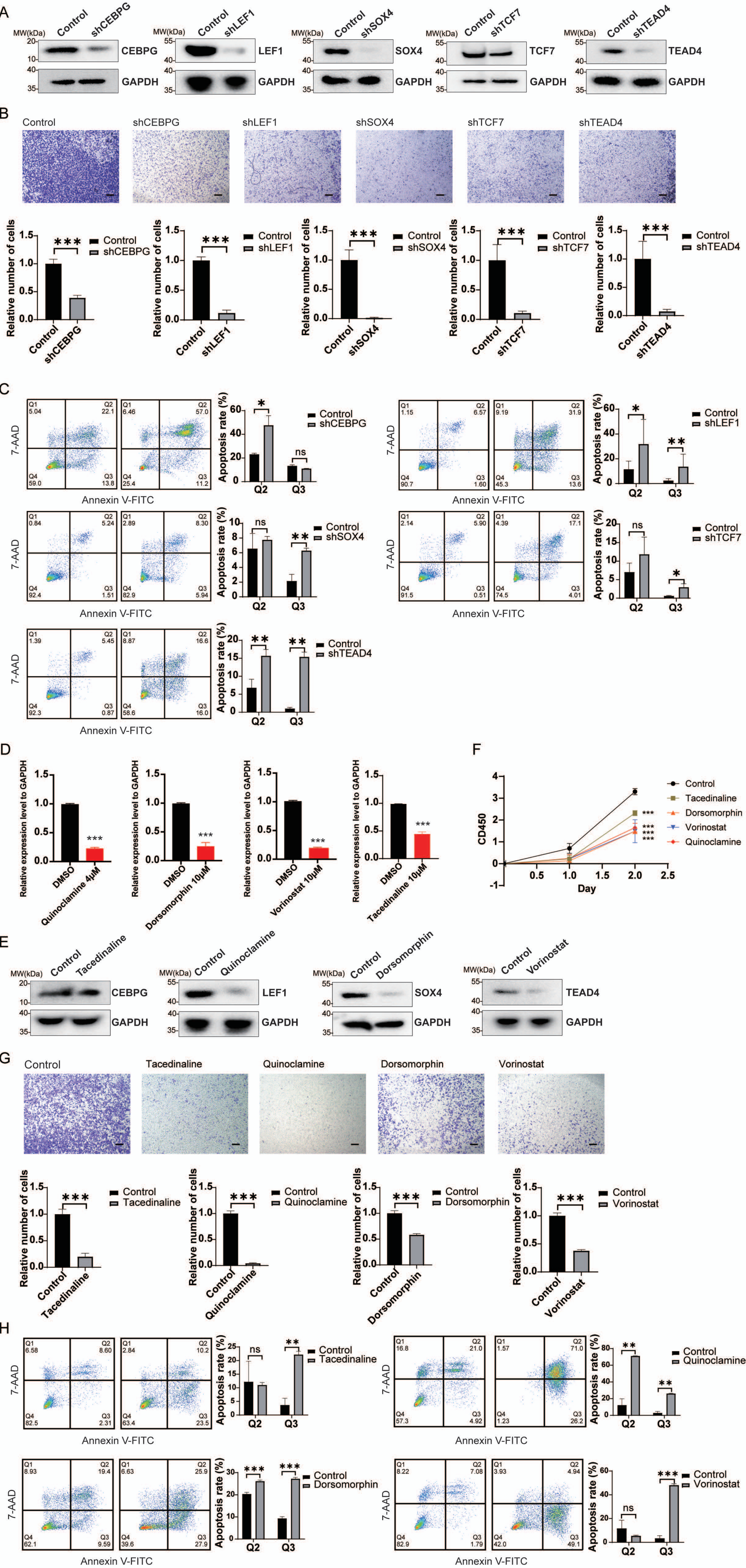
A
MW(kDa)
Control
shCEBPG
CEBPG
GAPDH
shLEF1
LEF1
shSOX4
SOX4
shTCF7
TCF7
shTEAD4
TEAD4
B
Control
shCEBPG
shLEF1
shSOX4
shTCF7
shTEAD4
Relative number of cells
***
C
7-AAD
Annexin V-FITC
Apoptosis rate (%)
Q1
Q2
Q3
Q4
*
**
ns
D
Relative expression level to GAPDH
DMSO
Quinoclamine 4μM
Dorsomorphin 10μM
Vorinostat 10μM
Tacedinaline 10μM
***
E
Tacedinaline
Quinoclamine
Dorsomorphin
Vorinostat
F
CD450
Day
Control
Tacedinaline
Dorsomorphin
Vorinostat
Quinoclamine
G
H

Supplementary Table S1. Selected scATAC-seq and scRNA-seq datasets

PMID	SampleID	DataType	State	Notes
35726067 (Colon cancer)	CRC-1-8810	snATAC-seq, snRNA-seq	Tumor	
	CRC-2-15564	snATAC-seq, snRNA-seq	Tumor	
	CRC-3-11773	snATAC-seq, snRNA-seq	Tumor	
	CRC-4-8456	snATAC-seq	Tumor	
	B001-A-401	snATAC-seq, snRNA-seq	Normal	
	B004-A-008	snATAC-seq, snRNA-seq	Normal	
	A001-C-223	snATAC-seq, snRNA-seq	Normal	
	A002-C-024	snATAC-seq, snRNA-seq	Normal	
	A014-C-052	snATAC-seq, snRNA-seq	Normal	
	A015-C-208	snATAC-seq, snRNA-seq	Normal	
	B001-A-406	snATAC-seq, snRNA-seq	Normal	
	B004-A-004(rep1)	snATAC-seq, snRNA-seq	Normal	
	B004-A-004(rep2)	snATAC-seq, snRNA-seq	Normal	
	B004-A-008	snATAC-seq, snRNA-seq	Normal	
	A001-C-007	snATAC-seq, snRNA-seq	Tumor	Patient 3[a]
	A001-C-023	snATAC-seq, snRNA-seq	Normal	Patient 3
	A015-C-001	snRNA-seq	Tumor	
	A001-C-123	snATAC-seq	Normal	Validation[b]
	A002-C-025	snATAC-seq	Normal	Validation
	A014-C-054	snATAC-seq	Normal	Validation
	A015-C-008	snATAC-seq	Normal	Validation
	B001-A-301	snATAC-seq	Normal	Validation
	B001-A-302	snATAC-seq	Normal	Validation
	B004-A-204	snATAC-seq	Normal	Validation
39236169 (Colon cancer)	X014_S04_B1_T1	snATAC-seq	Tumor	
	X005_S03_B1_T1	snATAC-seq	Tumor	
	X004_S03_B1_T1	snATAC-seq	Tumor	
	X013_S02_B1_T	snATAC-seq	Tumor	

	X013_S04_B1_T1	snATAC-seq	Tumor	Validation
	X010_S04_B1	snATAC-seq	Tumor	Validation
	X006_S03_B1	snATAC-seq	Tumor	Validation
	X011_S03_B1	snATAC-seq	Tumor	Validation
	X007_S03_B1	snATAC-seq	Tumor	Validation
	X010_S03_B1	snATAC-seq	Tumor	Validation
	X003_S04_B1	snATAC-seq	Tumor	Validation
	X009_S03_B1	snATAC-seq	Tumor	Validation
32451460 (Colon cancer)	All	scRNA-seq	Tumor, Normal	Validation
34739872 (Endometrial cancer)	Patient 1	snATAC-seq, snRNA-seq	Tumor	
	Patient 2	snATAC-seq, snRNA-seq	Tumor	
	Patient 3	snATAC-seq, snRNA-seq	Tumor	
	Patient 4	snATAC-seq, snRNA-seq	Tumor	
	Patient 5	snATAC-seq, snRNA-seq	Tumor	
34739872 (Ovarian cancer)	Patient 8	snATAC-seq, snRNA-seq	Tumor	
	Patient 9	snATAC-seq, snRNA-seq	Tumor	
36607615 (Renal cell cancer)	RCC81	snATAC-seq, snRNA-seq	Tumor	
	RCC84	snATAC-seq, snRNA-seq	Tumor	
	RCC86	snATAC-seq, snRNA-seq	Tumor	
	RCC87	snATAC-seq, snRNA-seq	Tumor	
	RCC94	snATAC-seq, snRNA-seq	Tumor	
	RCC96	snATAC-seq, snRNA-seq	Tumor	
	RCC99	snRNA-seq	Tumor	
39236169 (Renal cell cancer)	X007_S05_B1	snATAC-seq	Tumor	
	X005_S05_B1	snATAC-seq	Tumor	
	X006_S05_B1	snATAC-seq	Tumor	
	X012_S06_B1	snATAC-seq	Tumor	
31375813 (Basal cell cancer)	SU001 Tcell Post2	snATAC-seq	Tumor	
	SU001 Immune Post 2	snATAC-seq	Tumor	

	SU001 Total Post 2	snATAC-seq	Tumor	
	SU005 Total Post	snATAC-seq	Tumor	
	SU006 T cell Pre	snATAC-seq	Tumor	
	SU006 Stromal Pre	snATAC-seq	Tumor	
	SU007 Total Post	snATAC-seq	Tumor	
	SU008 Stromal Pre	snATAC-seq	Tumor	
	SU008 Immune Post	snATAC-seq	Tumor	
	SU008 Stromal Post	snATAC-seq	Tumor	
	SU009 Immune/Stromal Pre	snATAC-seq	Tumor	
	SU009 Immune/Stromal Post	snATAC-seq	Tumor	
	SU010 Total Pre	snATAC-seq	Tumor	
	SU010 Total Post	snATAC-seq	Tumor	
31359002 (Basal cell cancer)	All	scRNA-seq	Tumor	
37980571 (Primary liver cancer)	CCA_T_C39	snATAC-seq	Tumor	
	CCA_T_C56	snATAC-seq	Tumor	
	CCA_T_C60	snATAC-seq	Tumor	
	HCC_T_H23	snATAC-seq	Tumor	
	HCC_T_H30	snATAC-seq	Tumor	
	HCC_T_H38	snATAC-seq	Tumor	
	HCC_T_H58	snATAC-seq	Tumor	
	HCC_T_H62	snATAC-seq	Tumor	
	HCC_T_H63	snATAC-seq	Tumor	
	HCC_T_H65	snATAC-seq	Tumor	
	HCC_T_H69	snATAC-seq	Tumor	
	HCC_T_H70	snATAC-seq	Tumor	
	HCC_T_H77	snATAC-seq	Tumor	
	HCC_T_1HT1	snATAC-seq	Tumor	

	HCC_T_2HT1	snATAC-seq	Tumor	
	HCC_T_4HT1	snATAC-seq	Tumor	
34216724 (Primary liver cancer)	S004	scRNA-seq	Tumor	
	S007	scRNA-seq	Tumor	
	S011	scRNA-seq	Tumor	
	S012	scRNA-seq	Tumor	
	S015	scRNA-seq	Tumor	
	S016	scRNA-seq	Tumor	
	S019	scRNA-seq	Tumor	
	S022	scRNA-seq	Tumor	
	S023	scRNA-seq	Tumor	
	S028	scRNA-seq	Tumor	
	S029	scRNA-seq	Tumor	
	S031	scRNA-seq	Tumor	
	S035	scRNA-seq	Tumor	
	S036	scRNA-seq	Tumor	
	S038	scRNA-seq	Tumor	
	S045	scRNA-seq	Tumor	
39236169 (Breast cancer)	X004_S02_B1	snATAC-seq	Tumor	
	X010_S02_B1	snATAC-seq	Tumor	
	X011_S02_B1	snATAC-seq	Tumor	
	X005_S02_B1	snATAC-seq	Tumor	
	X001_S01_B1	snATAC-seq	Tumor	
	X003_S03_B1	snATAC-seq	Tumor	
	X007_S02_B1	snATAC-seq	Tumor	
	X003_S02_B1	snATAC-seq	Tumor	
	X013_S01_B1	snATAC-seq	Tumor	
	X009_S01_B1	snATAC-seq	Tumor	
	X006_S02_B1	snATAC-seq	Tumor	
	X009_S02_B1	snATAC-seq	Tumor	
	X008_S02_B1	snATAC-seq	Tumor	

	X013_S06_B1	snATAC-seq	Tumor	
	X012_S07_B1	snATAC-seq	Tumor	
	X014_S08_B1	snATAC-seq	Tumor	
34493872 (Breast cancer)	All	scRNA-seq	Tumor	
39236169 (Lung cancer)	X011_S06_B1	snATAC-seq	Tumor	
	X014_S01_B1	snATAC-seq	Tumor	
	X003_S06_B1	snATAC-seq	Tumor	
	X008_S05_B1	snATAC-seq	Tumor	
	X005_S06_B1	snATAC-seq	Tumor	
	X002_S01_B1	snATAC-seq	Tumor	
	X007_S06_B1	snATAC-seq	Tumor	
	X009_S06_B1	snATAC-seq	Tumor	
	X006_S06_B1	snATAC-seq	Tumor	
	X010_S07_B1	snATAC-seq	Tumor	
	X004_S06_B1	snATAC-seq	Tumor	
33953163 (Lung cancer)	P2	scRNA-seq	Tumor	
	P5	scRNA-seq	Tumor	
	P8	scRNA-seq	Tumor	
	P9	scRNA-seq	Tumor	
	P12	scRNA-seq	Tumor	
	P13	scRNA-seq	Tumor	
	P16	scRNA-seq	Tumor	
	P20	scRNA-seq	Tumor	
	P21	scRNA-seq	Tumor	
	P24	scRNA-seq	Tumor	
	P28	scRNA-seq	Tumor	
	P29	scRNA-seq	Tumor	
	P32	scRNA-seq	Tumor	
	P33	scRNA-seq	Tumor	
	P34	scRNA-seq	Tumor	

	P35	scRNA-seq	Tumor	
	P38	scRNA-seq	Tumor	
	P39	scRNA-seq	Tumor	

[a] Patient 3 represents data from Patient 3, which is used to validate the activation of tumor-specific TFs. [b] Validation represents validation data labeled as "Validation" in the article, which is used to validate the activation of tumor-specific TFs.

Table S2. Cell number of different single-cell sequencing data

CancerType	DataType	SampleNumber	CellNumber	PMID
BC	scATAC-seq	16	30000	39236169
	scRNA-seq	26	30000	34493872
BCC	scATAC-seq	14	12214	31375813
	scRNA-seq	33	29489	31359002
CC	scATAC-seq	8	27409	35726067、39236169
	scRNA-seq	5	14646	35726067
EC	scATAC-seq	5	29250	34739872
	scRNA-seq	5	31341	34739872
LC	scATAC-seq	11	23868	39236169
	scRNA-seq	18	22974	33953163
OC	scATAC-seq	2	13031	34739872
	scRNA-seq	2	12288	34739872
PLC	scATAC-seq	16	14231	37980571
	scRNA-seq	16	17971	34216724
RCC	scATAC-seq	10	26925	39236169、36607615
	scRNA-seq	7	44828	36607615

Table S3. Canonical markers for cell type annotation

**CellType**	**Gene**
B cell	*MS4A1*
Endothelial cell	*PLVAP*
Endothelial cell	*PECAM1*
Endothelial cell	*EMCN*
Fibroblast	*PDGFRA*
Fibroblast	*PDGFRB*
Fibroblast	*COL8A1*
GC B cell	*FCRLA*
Glia	*NCAM1*
Lymphocyte	*CD3D*
Mast	*KIT*
Mast	*SLC18A2*
Melanocyte	*MLANA*
Myeloid cell	*CD163*
Myeloid cell	*ITGAX*
Myofibroblast	*ACTA2*
Myofibroblast	*TAGLN*
Neuron	*CPE*
NK	*NCR1*
Plasma cell	*JCHAIN*
Plasma cell	*FCRL5*
T cell	*CD247*
Tumor cell	*EPCAM*
Colon cancer cell	*LGR5*
Colon cancer cell	*KRT8*
Endometrial cancer cell	*SCGB2A1*
Ovarian cancer cell	*KRT6A*
Renal cancer cell	*CA9*
Basal cell cancer cell	*KRT14*
Primary liver cancer cell	*KRT18*
Primary liver cancer cell	*APOA2*
Breast cancer cell	*KRT8*
Breast cancer cell	*KRT18*
Breast cancer cell	*ERBB2*
Lung cancer cell	*FOXA1*

Table S4. Differentially accessible regions of all cell types in eight distinct cancers

**Cancer Type**	**CellType**	**Number of DARs**	**Number of cell-type-specific cancer-associated DARs**	**Proportion of cell-type-specific cancer-associated DARs**
Breast cancer	Endothelial	2998	315	0.10507
	Fibroblast	4895	1227	0.250664
	Lymphocyte	637	637	1
	Myeloid cell	3289	209	0.063545
	Myofibroblast	2907	2191	0.753698
	Tumor cell	2325	881	0.378925
Basal cell cancer	B cell	1298	174	0.134052
	Endothelial	3222	502	0.155804
	Melanocyte	131	131	1
	Myeloid cell	1122	100	0.089127
	Myofibroblast	6760	2191	0.324112
	NK	1375	1375	1
	Plasma cell	2148	701	0.32635
	T cell	5628	1310	0.232765
	Tumor cell	9508	6176	0.649558
Colon cancer	B cell	1338	550	0.411061
	Endothelial	1890	125	0.066138
	Fibroblast	4660	1386	0.297425
	Myeloid cell	1699	44	0.025898
	T cell	1323	65	0.049131
	Tumor cell	5298	2860	0.539826
Endometrial cancer	B cell	2863	1143	0.399232
	Endothelial	4526	983	0.21719
	Fibroblast	4941	2962	0.599474
	Mast	1230	1230	1
	Myeloid cell	2898	333	0.114907
	Myofibroblast	2563	604	0.235661
	T cell	4317	356	0.082465
	Tumor cell	1146	147	0.128272
Lung cancer	B cell	1383	333	0.240781
	Endothelial	2944	316	0.107337
	Fibroblast	4208	758	0.180133
	Myeloid cell	6444	1673	0.259621
	Myofibroblast	5171	1876	0.362792
	Plasma cell	6710	3879	0.578092
	T cell	503	5	0.00994
	Tumor cell	3740	1701	0.454813
Ovarian cancer	Endothelial	4266	1151	0.269808
	Fibroblast	2349	869	0.369945

	Myeloid cell	3596	837	0.232759
	Myofibroblast	4463	1232	0.276048
	Plasma cell	833	145	0.17407
	T cell	6776	2920	0.430933
	Tumor cell	3666	1568	0.427714
Primary liver cancer	B cell	1659	319	0.192285
	Endothelial	4029	709	0.175974
	Myeloid cell	1384	60	0.043353
	Myofibroblast	2389	259	0.108414
	Plasma cell	1771	224	0.126482
	T cell	2239	78	0.034837
	Tumor cell	1902	614	0.322818
Renal Cell cancer	Endothelial	6792	2053	0.302267
	Myeloid cell	4693	778	0.165779
	Myofibroblast	4171	1313	0.314793
	Plasma cell	2948	662	0.224559
	T cell	1932	157	0.081263
	Tumor cell	10122	6871	0.678818

Table S5. Overlap between GWAS SNPs and scATAC-seq peaks

**scATAC-seq peaks**	**GWAS SNPs**						
	**BC**	**CC**	**EC**	**LC**	**OC**	**PLC**	**RCC**
**BCC**	117	85	0	15	24	4	3
**BC**	267	142	2	27	48	7	5
**CC**	181	174	7	21	34	3	2
**EC**	314	191	1	22	52	11	11
**LC**	230	132	6	27	41	5	6
**OC**	292	197	5	21	60	7	6
**PLC**	151	133	1	18	25	3	1
**RCC**	162	109	2	25	28	7	6

Table S6. LDSC GWAS enrichment of cell types in scATAC-seq from colon cancer, ovarian cancer and breast cancer

CancerType	CellType	Disease	P_Value
Colon cancer	B_cell	Balding, Loh et al.	0.063756
	B_cell	BMI, Vogelezang et al.	0.477125
	B_cell	BH, Yengo et al.	0.95353
	B_cell	CAD, Jiang et al.	0.389922
	B_cell	CD, de Lange et al.	0.028147
	B_cell	CC, Rashkin et al.	0.956665
	B_cell	RA, Ishigaki et al.	0.006923
	B_cell	SLE, Wang et al.	0.007942
	B_cell	T2D, Jiang et al.	0.632878
	B_cell	UC, de Lange et al.	0.423681
	Endothelial	Balding, Loh et al.	0.530669
	Endothelial	BMI, Vogelezang et al.	0.163729
	Endothelial	BH, Yengo et al.	0.697915
	Endothelial	CAD, Jiang et al.	0.264671
	Endothelial	CD, de Lange et al.	0.57332
	Endothelial	CC, Rashkin et al.	0.513244
	Endothelial	RA, Ishigaki et al.	0.464726
	Endothelial	SLE, Wang et al.	0.863502
	Endothelial	T2D, Jiang et al.	0.973277
	Endothelial	UC, de Lange et al.	0.915461
	Fibroblast	Balding, Loh et al.	0.14116
	Fibroblast	BMI, Vogelezang et al.	0.23975
	Fibroblast	BH, Yengo et al.	0.033855
	Fibroblast	CAD, Jiang et al.	0.049617
	Fibroblast	CD, de Lange et al.	0.07474
	Fibroblast	CC, Rashkin et al.	0.045238
	Fibroblast	RA, Ishigaki et al.	0.695243
	Fibroblast	SLE, Wang et al.	0.858267
	Fibroblast	T2D, Jiang et al.	0.185656
	Fibroblast	UC, de Lange et al.	0.776428
	Myeloid_cell	Balding, Loh et al.	0.520158
	Myeloid_cell	BMI, Vogelezang et al.	0.600244
	Myeloid_cell	BH, Yengo et al.	0.932928
	Myeloid_cell	CAD, Jiang et al.	0.644645
	Myeloid_cell	CD, de Lange et al.	0.035331
	Myeloid_cell	CC, Rashkin et al.	0.269884
	Myeloid_cell	RA, Ishigaki et al.	0.182062
	Myeloid_cell	SLE, Wang et al.	0.049112
	Myeloid_cell	T2D, Jiang et al.	0.208227
	Myeloid_cell	UC, de Lange et al.	0.527285

	T_cell	Balding, Loh et al.	0.288085
	T_cell	BMI, Vogelezang et al.	0.755046
	T_cell	BH, Yengo et al.	0.849914
	T_cell	CAD, Jiang et al.	0.168393
	T_cell	CD, de Lange et al.	0.249942
	T_cell	CC, Rashkin et al.	0.857535
	T_cell	RA, Ishigaki et al.	0.009927
	T_cell	SLE, Wang et al.	0.026168
	T_cell	T2D, Jiang et al.	0.255169
	T_cell	UC, de Lange et al.	0.109392
	Tumor_cell	Balding, Loh et al.	0.855044
	Tumor_cell	BMI, Vogelezang et al.	0.275147
	Tumor_cell	BH, Yengo et al.	0.997685
	Tumor_cell	CAD, Jiang et al.	0.278418
	Tumor_cell	CD, de Lange et al.	0.973688
	Tumor_cell	CC, Rashkin et al.	0.006919
	Tumor_cell	RA, Ishigaki et al.	0.745603
	Tumor_cell	SLE, Wang et al.	0.884617
	Tumor_cell	T2D, Jiang et al.	0.26253
	Tumor_cell	UC, de Lange et al.	0.037841
Ovarian cancer	Endothelial	Balding, Loh et al.	0.587397
	Endothelial	BMI, Vogelezang et al.	0.307387
	Endothelial	BH, Yengo et al.	0.214745
	Endothelial	CAD, Jiang et al.	0.064455
	Endothelial	CD, de Lange et al.	0.992354
	Endothelial	OC, Jiang et al.	0.514118
	Endothelial	RA, Ishigaki et al.	0.874597
	Endothelial	SLE, Wang et al.	0.419456
	Endothelial	T2D, Jiang et al.	0.163495
	Endothelial	UC, de Lange et al.	0.925761
	Fibroblast	Balding, Loh et al.	0.06139
	Fibroblast	BMI, Vogelezang et al.	0.55081
	Fibroblast	BH, Yengo et al.	0.359792
	Fibroblast	CAD, Jiang et al.	0.533385
	Fibroblast	CD, de Lange et al.	0.494702
	Fibroblast	OC, Jiang et al.	0.012693
	Fibroblast	RA, Ishigaki et al.	0.999339
	Fibroblast	SLE, Wang et al.	0.977278
	Fibroblast	T2D, Jiang et al.	0.090687
	Fibroblast	UC, de Lange et al.	0.905588
	Myeloid_cell	Balding, Loh et al.	0.105679
	Myeloid_cell	BMI, Vogelezang et al.	0.331246
	Myeloid_cell	BH, Yengo et al.	0.435338

	Myeloid_cell	CAD, Jiang et al.	0.281375
	Myeloid_cell	CD, de Lange et al.	0.037334
	Myeloid_cell	OC, Jiang et al.	0.344708
	Myeloid_cell	RA, Ishigaki et al.	0.40845
	Myeloid_cell	SLE, Wang et al.	0.65864
	Myeloid_cell	T2D, Jiang et al.	0.56546
	Myeloid_cell	UC, de Lange et al.	0.2285
	Myofibroblast	Balding, Loh et al.	0.010961
	Myofibroblast	BMI, Vogelezang et al.	0.328427
	Myofibroblast	BH, Yengo et al.	0.051372
	Myofibroblast	CAD, Jiang et al.	0.013992
	Myofibroblast	CD, de Lange et al.	0.56443
	Myofibroblast	OC, Jiang et al.	0.008304
	Myofibroblast	RA, Ishigaki et al.	0.975893
	Myofibroblast	SLE, Wang et al.	0.96557
	Myofibroblast	T2D, Jiang et al.	0.29682
	Myofibroblast	UC, de Lange et al.	0.717934
	Plasma	Balding, Loh et al.	0.836633
	Plasma	BMI, Vogelezang et al.	0.010691
	Plasma	BH, Yengo et al.	0.211199
	Plasma	CAD, Jiang et al.	0.967067
	Plasma	CD, de Lange et al.	0.326159
	Plasma	OC, Jiang et al.	0.356066
	Plasma	RA, Ishigaki et al.	0.092324
	Plasma	SLE, Wang et al.	0.092141
	Plasma	T2D, Jiang et al.	0.817477
	Plasma	UC, de Lange et al.	0.262658
	T_cell	Balding, Loh et al.	0.599264
	T_cell	BMI, Vogelezang et al.	0.016301
	T_cell	BH, Yengo et al.	0.241101
	T_cell	CAD, Jiang et al.	0.43853
	T_cell	CD, de Lange et al.	0.27691
	T_cell	OC, Jiang et al.	0.659232
	T_cell	RA, Ishigaki et al.	0.008691
	T_cell	SLE, Wang et al.	0.188619
	T_cell	T2D, Jiang et al.	0.508415
	T_cell	UC, de Lange et al.	0.032483
	Tumor_cell	Balding, Loh et al.	0.267232
	Tumor_cell	BMI, Vogelezang et al.	0.349753
	Tumor_cell	BH, Yengo et al.	0.310335
	Tumor_cell	CAD, Jiang et al.	0.075107
	Tumor_cell	CD, de Lange et al.	0.382515
	Tumor_cell	OC, Jiang et al.	0.631987

	Tumor_cell	RA, Ishigaki et al.	0.639073
	Tumor_cell	SLE, Wang et al.	0.995641
	Tumor_cell	T2D, Jiang et al.	0.254728
	Tumor_cell	UC, de Lange et al.	0.975407
Breast cancer	Endothelial	Balding, Loh et al.	0.586006
	Endothelial	BMI, Vogelezang et al.	0.327886
	Endothelial	BH, Yengo et al.	0.632835
	Endothelial	BC, Rashkin et al.	0.302553
	Endothelial	CAD, Jiang et al.	0.008541
	Endothelial	CD, de Lange et al.	0.896386
	Endothelial	RA, Ishigaki et al.	0.563819
	Endothelial	SLE, Wang et al.	0.850209
	Endothelial	T2D, Jiang et al.	0.608813
	Endothelial	UC, de Lange et al.	0.963408
	Fibroblast	Balding, Loh et al.	0.07586
	Fibroblast	BMI, Vogelezang et al.	0.572508
	Fibroblast	BH, Yengo et al.	0.009218
	Fibroblast	BC, Rashkin et al.	0.793996
	Fibroblast	CAD, Jiang et al.	0.007942
	Fibroblast	CD, de Lange et al.	0.486468
	Fibroblast	RA, Ishigaki et al.	0.668677
	Fibroblast	SLE, Wang et al.	0.915756
	Fibroblast	T2D, Jiang et al.	0.398805
	Fibroblast	UC, de Lange et al.	0.919291
	Lymphocyte	Balding, Loh et al.	0.068452
	Lymphocyte	BMI, Vogelezang et al.	0.128993
	Lymphocyte	BH, Yengo et al.	0.304856
	Lymphocyte	BC, Rashkin et al.	0.747393
	Lymphocyte	CAD, Jiang et al.	0.740125
	Lymphocyte	CD, de Lange et al.	0.188231
	Lymphocyte	RA, Ishigaki et al.	0.029364
	Lymphocyte	SLE, Wang et al.	0.019441
	Lymphocyte	T2D, Jiang et al.	0.657887
	Lymphocyte	UC, de Lange et al.	0.192375
	Myeloid_cell	Balding, Loh et al.	0.996943
	Myeloid_cell	BMI, Vogelezang et al.	0.475767
	Myeloid_cell	BH, Yengo et al.	0.583364
	Myeloid_cell	BC, Rashkin et al.	0.847103
	Myeloid_cell	CAD, Jiang et al.	0.364611
	Myeloid_cell	CD, de Lange et al.	0.044539
	Myeloid_cell	RA, Ishigaki et al.	0.078282
	Myeloid_cell	SLE, Wang et al.	0.078666
	Myeloid_cell	T2D, Jiang et al.	0.09959

	Myeloid_cell	UC, de Lange et al.	0.190877
	Myofibroblast	Balding, Loh et al.	0.325587
	Myofibroblast	BMI, Vogelezang et al.	0.100949
	Myofibroblast	BH, Yengo et al.	0.067158
	Myofibroblast	BC, Rashkin et al.	0.507517
	Myofibroblast	CAD, Jiang et al.	0.005833
	Myofibroblast	CD, de Lange et al.	0.377738
	Myofibroblast	RA, Ishigaki et al.	0.996626
	Myofibroblast	SLE, Wang et al.	0.368455
	Myofibroblast	T2D, Jiang et al.	0.688959
	Myofibroblast	UC, de Lange et al.	0.988064
	Tumor_cell	Balding, Loh et al.	0.27873
	Tumor_cell	BMI, Vogelezang et al.	0.274911
	Tumor_cell	BH, Yengo et al.	0.379005
	Tumor_cell	BC, Rashkin et al.	0.008644
	Tumor_cell	CAD, Jiang et al.	0.479922
	Tumor_cell	CD, de Lange et al.	0.997246
	Tumor_cell	RA, Ishigaki et al.	0.718189
	Tumor_cell	SLE, Wang et al.	0.774128
	Tumor_cell	T2D, Jiang et al.	0.925737
	Tumor_cell	UC, de Lange et al.	0.682814

Table S7. Highly activated TFs of all cell types in eight distinct cancers

CancerType	CellType	TF
Breast cancer	Tumor cell	ASCL1(var.2), FOXA1,FOXA2, FOXA3, FOXB1, FOXC1, FOXC2, FOXD1, FOXD2, FOXE1, FOXF2, FOXG1, FOXI1, FOXK1, FOXK2, FOXL1, FOXN3, FOXO3, FOXO4, FOXO6, FOXP1, FOXP2, FOXP3, GRHL1, GRHL2, MYOD1, NFIA, NFIB, NFIX, SCRT1, SCRT2, TCF3, TCF4, TCF12(var.2), TFCP2, ZEB1
	Myofibroblast	EBF1, NFIA, NFIC, NFIX, SOX8, SOX9, SOX10, SOX15, TEAD1, TEAD2, TEAD3, TEAD4, TP53, TP63, TP73
	Myeloid cell	CEBPA, CEBPD, EHF, ELF1, ELF3, ELF5, ETV1, ETV4, ETV6, IKZF1, IRF1, IRF2, IRF3, IRF4, IRF7, IRF8, IRF9, MITF, NRL, SPI1, SPIB, SPIC, STAT1::STAT2, USF1, USF2, ZKSCAN5
	Fibroblast	NFATC2, NFATC3, NFATC4, TEAD1, TEAD2, TEAD3, TEAD4, TWIST1
	Endothelial	SOX8, SOX9, SOX15
	Lymphocyte	IRF2, IRF4, IRF7, IRF8, IRF9
Basal cell cancer	Tumor cell	ALX3, ARGFX, ASCL1(var.2), EMX1, EMX2, GRHL1, GRHL2, HOXD3, LBX1, LHX5, LHX6, LMX1A, LMX1B, MEOX1, MEOX2, MIXL1, MNX1, NFIA, NFIB, NFIC, NFIX, NKX6-1, NKX6-2, NOTO, PAX4, PHOX2A, PHOX2B, POU4F1, POU4F2, POU4F3, POU6F1, POU6F2, PROP1, TEAD1, TEAD2, TEAD3, TEAD4, TFAP2A, TFAP2B(var.2), TFAP2B(var.3), TFAP2B, TFAP2C(var.2), TFAP2C(var.3), TFAP2C, TFCP2, TP53, TP63, TP73, VAX1, VSX1, VSX2, ZNF75D, ZEB1
	Myofibroblast	CEBPA, CEBPD, EBF1, EBF3, MEIS2(var.2), NFATC2, PBX2, POU4F1, POU4F2, POU4F3, TEAD1, TEAD2, TEAD3, TEAD4, TWIST1, ZBTB18
	Myeloid cell	CEBPA, CEBPD, CEBPG(var.2), EHF, ELF1, IKZF1, SPI1, SPIB, SPIC
	Plasma	POU1F1, POU2F1, POU2F2, POU2F3, POU3F1, POU3F2, POU3F3, POU3F4, POU5F1, POU5F1B
	B cell	IRF1, POU1F1, POU2F1, POU2F2, POU2F3, POU3F1, POU3F2, POU3F3, POU3F4, POU5F1, POU5F1B
	NK	EOMES, MGA, TBR1, TBX1, TBX2, TBX3, TBX4, TBX5, TBX20, TBX21
Colon cancer	Tumor cell	ASCL1(var.2), ASCL1, CDX1, CDX2, CDX4, FIGLA, FOXA1, FOXA2, FOXA3, FOXB1, FOXC1, FOXC2, FOXD1, FOXD2, FOXE1, FOXF2, FOXG1, FOXI1, FOXK1, FOXK2, FOXL1,

		FOXO3, FOXO4, FOXO6, FOXP1, FOXP2, FOXP3, GATA2, GATA3, GATA4, GATA5, GATA6, GRHL1, GRHL2, HNF1A, HNF1B, HNF4A, HNF4A(var.2), HNF4G, HOXA6, HOXA10, HOXA13, HOXB13, HOXB9, HOXC10, HOXC11, HOXC12, HOXC13, HOXD9, HOXD10, HOXD11, HOXD12, HOXD13, LEF1, MYOD1, NFIA, NFIB, NFIC(var.2), NFIX, NFIX(var.2), NR1H2::RXRA, NR1I3, NR2C2, NR2F1(var.2), PPARA::RXRA, PPARD, RXRB, RXRG, SCRT1, SCRT2, SNAI1, SNAI2, SNAI3, SOX10, SOX4, TCF12(var.2), TCF3, TCF4, TCF7, TCF7L1, TCF7L2, TEAD1, TEAD3, TEAD4, THRB, ZEB1
	Fibroblast	NFATC2, TAL1::TCF3, TEAD1, TEAD2, TEAD3, TEAD4, TWIST1
	Endothelial	SOX13, SOX15, SOX2, SOX8, SOX9
	Myeloid cell	CEBPA, CEBPD, HLF, IKZF1, NFIL3, SPI1, SPIB, SPIC
	T cell	EHF, ELF1, ELF2, ELF3, ELF4, ELF5, ELK3, ELK4, ERF, ERG, ETS1, ETS2, ETV1, ETV2, ETV3, ETV4, ETV5, ETV6, FEV, FLI1, GABPA, KLF14, NFYA, NFYB, NFYC, NRF1, RUNX2, RUNX3, SPEDF, ZBTZ7A, ZBTB14
	B cell	IRF2, IRF3, IRF4, IRF7, IRF8, IRF9, POU1F1, POU2F1, POU2F2, POU2F3, POU3F1, POU3F2, POU3F3, POU3F4, POU5F1, POU5F1B
Endometrial cancer	Tumor cell	ASCL1(var.2), ESR2, FIGLA, FOXA1, FOXA2, FOXA3, FOXB1, FOXC1, FOXC2, FOXD1, FOXD2, FOXE1, FOXF2, FOXG1, FOXI1, FOXK1, FOXK2, FOXL1, FOXN3, FOXO3, FOXO4,
		FOXO6, FOXP1, FOXP2, FOXP3, GRHL1, GRHL2, HNF1A, HNF1B, HNF4A, HNF4G, HOXA1, HOXA6, HOXB3, HOXB6, HOXB7, LEF1, MYOD1, NFIA, NFIB, NFIC, NFIC(var.2), NFIC::TLX1, NFIX, NFIX(var.2), NHLH2, ONECUT1, PAX1, PAX6, PAX9, POU4F1, POU4F2, POU4F3, REST, SIX1, SIX2, SNAI1, SNAI2, SNAI3, SOX10, SOX12, SOX13, SOX14, SOX15, SOX18, SOX2, SOX4, SOX8, SOX9, SRY, TCF3, TCF4, TCF7, TCF12(var.2), TCF7L2, TEAD1, TEAD2, TEAD3, TEAD4, TFAP2A, TFAP2B, TFAP2C, TFCP2, ZEB1, ZNF449
	Fibroblast	ARGFX, CDX1, CDX2, CDX4, HAND2, HOXA10, HOXA13, HOXB13, HOXD9, TAL1::TCF3, TWIST1, ZBTB18, ZNF75D
	Myofibroblast	ARGFX, CDX1, CDX2, CDX4, HAND2, HOXA10, HOXA13, HOXB13, HOXD9, TAL1::TCF3, TWIST1, ZBTB18, ZNF75D
	Endothelial	EBF1, EBF3, PBX2
	Myeloid cell	STAT1, STAT3
	T cell	CEBPA, CEBPB, CEBPD, CEBPE, CEBPG, EHF, ELF3, ETV4,

		GABPA, HLF, IKZF1, NFIL3, SPI1, SPIB, SPIC, ZKSCAN5
	B cell	ELK4, EOMES, ETV1, ETV2, GABPA, RUNX2, RUNX3, TBR1, TBX1, TBX2, TBX20, TBX21
	Mast	IRF4, IRF7, IRF8, IRF9, POU1F1, POU2F1, POU2F2, POU2F3, POU3F1, POU3F2, POU3F3, POU3F4, POU5F1, POU5F1B
Ovarian cancer	Tumor cell	ASCL1(var.2), GRHL1, GRHL2, NFIC, PAX1, PAX9, SIX1, SIX2, SOX10, SOX13, SOX14, SOX15, SOX2, SOX4, SOX8, SOX9, TCF12(var.2), TCF3, TCF4, TEAD1, TEAD2, TEAD3, TEAD4, TFAP2A, TFAP2A(var.2), TFAP2B, TFAP2C, TFAP2C(var.2), TFAP2E, TFAP2B(var.2), TFAP2B(var.3), TFCP2, ZEB1
	Fibroblast	ATOH1(var.2), ATOH7, BHLHA15(var.2), BHLHE22(var.2), HAND2, HOXB4, MXI1, MYF5, MYF6, MYOG, NFATC2, NFATC3, NFATC4, TAL1::TCF3, TFAP4, TFAP4(var.2), TWIST1, ZBTB18
	Myofibroblast	EBF1, EBF3, NR3C1, NR3C2, NR4A1
	Myeloid cell	SPI1, SPIB, SPIC
	T cell	EHF, ELF1, ELF3, ELF4, ELK1, ELK3, ELK4, EOMES, ERF, ERG, ETS1, ETS2, ETV1, ETV2, ETV3, ETV4, ETV5, ETV6, FEV, FLI1, GABPA, IKZF1, RUNX2, RUNX3, SPDEF, TBR1, TBX2, TBX20, TBX21, ZBTB7A
	Plasma	IRF2, IRF3, IRF4, IRF5, IRF7, IRF8, IRF9, POU1F1, POU2F1, POU2F2, POU2F3, POU3F1, POU3F2, POU3F3, POU3F4, POU5F1, POU5F1B, STAT1::STAT2
Lung cancer	Tumor cell	ASCL1(var.2), FIGLA, FOXA1, FOXA2, FOXA3, FOXB1, FOXC1, FOXC2, FOXD1, FOXD2, FOXE1, FOXF2, FOXG1, FOXI1, FOXK1, FOXK2, FOXL1, FOXO3, FOXO4, FOXO6, FOXP1, FOXP2, FOXP3, GRHL1, GRHL2, HNF1A, HNF1B, HNF4A(var.2), HNF4A, HNF4G, MSANTD3, MYOD1, NFIA, NFIB, NFIC(var.2), NFIC, NFIX(var.2), NFIX, NKX2-2, NKX2-3, NKX2-5, NKX2-8, NR1H2::RXRA, NR4A1, NR6A1, ONECUT1, PKNOX2, POU4F1, POU4F2, RXRG, SCRT1, SCRT2, SNAI1, SOX4, SOX10, TCF3, TCF4, TCF7, TCF7L1, TCF7L2, TCF12(var.2), TEAD1, TEAD2, TEAD3, TEAD4, TFCP2, ZEB1, ZNF24
	Myofibroblast	TEAD1, TEAD2, TEAD3, TEAD4
	Myeloid cell	CEBPA, CEBPD, EHF, ELF1, ELF3, ELF5, ETV1, ETV4, ETV6, GABPA, HLF, IKZF1, IRF1, IRF2, IRF3, IRF4, NFIL3, SPI1, SPIB, SPIC, STAT1::STAT2, USF1, USF2, ZKSCAN5
	Plasma	ASCL1(var.2), FIGLA, IRF1, IRF2, IRF3, IRF4, IRF7, IRF8,

		IRF9, MYOD1, POU1F1, POU2F1, POU2F2, POU2F3, POU3F1, POU3F2, POU3F3, POU3F4, POU5F1, POU5F1B, STAT1::STAT2, TCF3, TCF4, TCF12(var.2)
	B cell	POU2F1, POU2F2
	Endothelial	SOX2, SOX8, SOX9, SOX13, SOX14
	Fibroblast	RUNX2, TEAD1, TEAD2, TEAD3, TEAD4
Primary liver cancer	Tumor cell	ATOH1(var.2), BARX2, BHLHA15(var.2), CUX1, CUX2, ESRRA, FOXA1, FOXA2, FOXA3, FOXB1, FOXC1, FOXC2, FOXD1, FOXD2, FOXE1, FOXF2, FOXG1, FOXI1, FOXK1, FOXN3, FOXO3, FOXO4, FOXO6, FOXP1, FOXP2, FOXP3, GATA2, GATA3, GATA4, GATA5, GATA6, HNF1A, HNF1B, HNF4A(var.2), HNF4A, HNF4G, MSC, MYF5, MYF6, NEUROD1, NEUROG2(var.2), NR1H2::RXRA, NR2F1(var.2), ONECUT1, ONECUT2, ONECUT3, PAX3, PAX7, PHOX2B, POU4F1, POU4F2, POU4F3, POU6F1, PPARA::RXRA, PPARD, PROP1, RXRB, RXRG, SOX4, SOX10, TAL1::TCF3, TCF7L1, TEAD1, TEAD2, TEAD3, TEAD4, THRB, ZNF24
	Myofibroblast	EBF1, EBF3
	Endothelial	SOX2, SOX8, SOX9
	Myeloid cell	CEBPA, CEBPB, CEBPD, CEBPE, CEBPG(var.2), CEBPG, EHF, ELF1, ELF3, ETV4, HLF, IKZF1, NFIL3, SPI1, SPIB, SPIC
	T cell	EOMES, RUNX2, RUNX3
	Plasma	ASCL1(var.2), ATOH1(var.2), BHLHA15(var.2), IRF2, IRF3, IRF4, IRF5, IRF6, IRF7, IRF8, IRF9, MSC, MYF5, MYF6, MYOD1, POU1F1, POU2F1, POU2F2, POU2F3, POU3F1, POU3F2, POU3F3, POU3F4, POU5F1, POU5F1B, SNAI1, SNAI2, SNAI3, TAL1::TCF3, TCF3, TCF4, TCF12(var.2), ZEB1
	B cell	POU1F1, POU2F1, POU2F2, POU2F3, POU3F1, POU3F2, POU3F3, POU3F4, POU5F1, POU5F1B
Renal cell cancer	Tumor cell	ARGFX, BARX2, DRGX, EMX2, ESR2, ESRRA, ESX1, EVX1, EVX2, GSX1, HMBOX1, HNF1A, HNF1B, HNF4A, HNF4A(var.2), HNF4G, HOXA6, HOXB3, HOXB4, HOXB7, HOXC4, HOXD4, LHX5, LHX6, LMX1A, LMX1B, MEOX1, MEOX2, MNX1, NFIA, NFIB, NFIC, NFIC(var.2), NFIC::TLX1, NFIX, NFIX(var.2), NKX6-1, NKX6-2, NKX6-3, NOTO, NR1H2::RXRA, NR1H4, NR1H4::RXRA, NR1I3, NR2C2, NR2F1(var.2), NR2F2, NR4A1, NR4A2::RXRA, PAX1, PAX3, PAX6, PAX7, PAX9, PDX1, POU4F1, POU4F2, POU4F3, POU6F1, POU6F1(var.2), POU6F2, PPARA::RXRA, PPARD,

		PRRX2, RAX,
	Myofibroblast	EBF1, EBF3
	Endothelial	FOXN3, SOX2, SOX4, SOX8, SOX9, SOX10, SOX13, SOX14, SOX15, SRY, STAT1, STAT3
	Myeloid cell	CEBPA, CEBPB, CEBPD, CEBPE, CEBPG(var.2), CEBPG, EHF, ELF1, ELF3, ELF5, ETV1, ETV4, ETV6, GABPA, HLF, IKZF1, NFIL3, SPI1, SPIB, SPIC, ZKSCAN5
	T cell	EOMES, MGA, RUNX2, RUNX3, TBR1, TBX1, TBX2, TBX3, TBX4, TBX5, TBX6, TBX15, TBX18, TBX20, TBX21,
	Plasma	ASCL1, ASCL1(var.2), FIGL1, IRF2, IRF3, IRF4, IRF5, IRF6, IRF7, IRF8, IRF9, MSC, MYF5, MYF6, MYOD1, POU1F1, POU2F1, POU2F2, POU2F3, POU3F1, POU3F2, POU3F3, POU3F4, POU5F1, POU5F1B, SCRT1, SCRT2, SNAI1, SNAI2, SNAI3, TCF3, TCF4, TCF12(var.2), ZBTB12, ZEB1

Table S8. Comparison of the observed normalized values for tumor-specific TFs between tumor cell and normal epithelial cells

scATAC-seq datasets	TFs	P_value(±100bp)	P_value(±250bp)
Tumor sample n = 8; Normal sample n = 9	CEBPG	0.00236609	0.56133817
	LEF1	5.11E-40	3.05E-13
	SOX4	7.53E-09	0.046825627
	TCF7	4.93E-43	2.46E-12
	TEAD4	1.53E-09	0.098392537
patient1	CEBPG	0.186952239	0.335116522
	LEF1	5.71E-11	1.41E-11
	SOX4	0.001493323	0.004085506
	TCF7	1.93E-10	4.29E-11
	TEAD4	0.013425328	0.037052958
patient2	CEBPG	2.15E-06	0.000847539
	LEF1	2.79E-10	2.02E-10
	SOX4	0.048709279	0.07781683
	TCF7	1.27E-09	5.73E-09
	TEAD4	0.001055246	0.00086777
patient3	CEBPG	1.15E-13	5.37E-07
	LEF1	2.65E-24	3.65E-15
	SOX4	1.21E-08	1.53E-05
	TCF7	1.59E-24	3.38E-14
	TEAD4	1.31E-13	3.36E-07
Validation (Tumor sample n = 8; Normal sample n = 7)	CEBPG	0.094114525	0.86906192
	LEF1	2.73E-34	1.70E-09
	SOX4	7.75E-07	0.11642535
	TCF7	1.34E-37	7.09E-10
	TEAD4	7.30E-05	0.221384762

Table S9. Signature genes of each meta-program

**metaProgram1**	**metaProgram2**	**metaProgram3**	**metaProgram4**
NKD1	*ARHGAP26*	*RPL13*	*DIAPH3*
DACH1	*NR3C2*	*RPS19*	*BRIP1*
KIZ	*RBFOX1*	*RPL8*	*BRCA2*
AC007277.1	*CAMK1D*	*RPL35*	*CENPP*
CERS6	*NEAT1*	*RPL28*	*ATAD2*
NCKAP5	*ZSWIM6*	*RPL36*	*FANCA*
SNTB1	*TCF7L2*	*MT-CO2*	*AC016205.1*
IMMP2L	*MYO1D*	*RPS15*	*BRCA1*
PBX1	*AKAP13*	*MT-CO3*	*RBL1*
DNAJC15	*FRYL*	*GPX2*	*POLQ*
TRPM3	*MYO1E*	*PTMA*	*EZH2*
RNF43	*SAT1*	*RPL13A*	*MELK*
APBB2	*DOCK1*	*ACTB*	*ATAD5*
NAALADL2	*PTPRK*	*EEF2*	*NCAPG2*
LINC01811	*3-Mar*	*RPLP1*	*NSD2*
DNAH14	*LINC02086*	*AC105402.3*	*FANCI*
PATJ	*DIAPH2*	*RPL29*	*HELLS*
TULP4	*PTPRN2*	*RPS3*	*KNTC1*
LINC01748	*FAM13A*	*MT-CO1*	*ASPM*
GMDS-DT	*PTPRJ*	*MT-ND3*	*CIT*
SPATA6L	*PARD3*	*FTL*	*SMC4*
ARID1B	*TPM1*	*RPL37A*	*MMS22L*
MLLT3	*DAPK2*	*RPS9*	*RFC3*
NRXN3	*MAGI1*	*MT-ND4*	*DTL*
LINC01594	*ATP10B*	*RPS11*	*TPX2*
KANK1	*PRKG1*	*RPLP0*	*CEP128*
DENND4C	*FTX*	*TMSB10*	*AC091057.6*
MSI2	*GMDS*	*EEF1A1*	*CENPJ*
NOTUM	*EXT1*	*RPS18*	*CEP152*
ZNRF3	*CCSER1*	*RPS2*	*MKI67*
SYK	*MALAT1*	*MTRNR2L8*	*POLA1*
LAMA2	*PELI2*	*S100A6*	*VRK1*
EXOC4	*FOXP1*	*UBA52*	*CENPF*
KCNQ1OT1	*KIF13B*	*RPL3*	*SCLT1*
C9orf3	*ARL15*	*RASGEF1B*	*DNMT1*
RBM39	*DST*	*MTRNR2L12*	*BARD1*
FNDC3B	*USP53*	*RPLP2*	*PRIM2*
DENND1A	*ERBIN*	*SLC26A3*	*NASP*
NR6A1	*MID1*	*RPS6*	*PRKDC*
RUNX1	*NEDD4L*	*RPS8*	*CENPK*

ATXN1	*ZBTB20*	*RPL18*	*TOP2A*
CHD6	*AOAH*	*RPL37*	*Z94721.1*
PLCB1	*PPARG*	*INO80D*	*DLEU2*
NFAT5	*INSR*	*RPL4*	*GEN1*
APCDD1	*HEPH*	*RPSA*	*PBX3*
GCC2	*PRELID2*	*MT-ATP6*	*MIS18BP1*
STK39	*PLD1*	*RPL10*	*NDUFAF6*
WWOX	*ABCA5*	*MT-CYB*	*CDK5RAP2*
CUX1	*FOXO1*	*DHFR*	*DDX11*
NBEA	*SH3D19*	*RPL7A*	*MTBP*
CDK6	*MECOM*	*TPT1*	*MYO19*
CADPS2	*PDE4D*	*ATP5F1E*	*BAZ1B*
FTX	*CHRM3*	*LINGO1*	*CEP295*
KANSL1	*SYTL2*	*RPL11*	*RIF1*
EXOC6B	*GPBP1*	*FP671120.1*	*ANKRD36C*
HIBADH	*TAOK3*	*KRT8*	*SYNE2*
MGAM2	*CAPN8*	*TMSB4X*	*RANBP17*
CEMIP2	*LRMDA*	*NORAD*	*TFDP1*
VPS13B	*KDM6A*	*RPL27A*	*RAD18*
INPP4B	*PLS1*	*RPL6*	*RTTN*
MACROD2	*MAML2*	*JUND*	*CSE1L*
MGAT5	*CFTR*	*TXNRD1*	*ECT2*
CTTNBP2	*SHROOM3*	*HLA-A*	*SPIDR*
YAP1	*TCF12*	*PABPC1*	*MGME1*
ITPR2	*LPP*	*FP236383.1*	*FAF1*
LINC00511	*LRBA*	*RPL14*	*GPHN*
AC016831.7	*MUC12*	*MT-ND2*	*CASP8AP2*
OXR1	*FGD4*	*RPL32*	*RGS3*
PRKCA	*LIMA1*	*SLC27A6*	*AC073529.1*
RERE	*DNM2*	*RACK1*	*LMNB1*
PTK2	*SATB2*	*PPDPF*	*AP001347.1*
MIR4713HG	*VMP1*	*APOO*	*WWOX*
NCOA2	*KIAA1217*	*SOX9*	*POLE*
ZMYM2	*B4GALNT3*	*ACTG1*	*FANCC*
GTF2I	*PID1*	*B2M*	*WDR34*
CCSER1	*ARHGEF28*	*RPS27A*	*MPHOSPH9*
PVT1	*KAZN*	*HSPB1*	*ZRANB3*
PARD3B	*CEACAM1*	*MT-ND5*	*MSH2*
LEF1	*GPCPD1*	*RPS14*	*PVT1*
TBC1D5	*KDM4C*	*ZFP36L2*	*MTHFD1L*
PLEKHA5	*BAIAP2L1*	*RPL19*	*SMPD4*
FOXO3	*DIP2B*	*RPL15*	*CEP192*
STK38	*PIP5K1B*	*EEF1D*	*SRPK1*

MTSS1	*SSH2*	*ZNF703*	*NDC1*
CDC14B	*FLNB*	*RPS29*	*TOPBP1*
PAN3	*ATP9A*	*RPS5*	*TBCD*
DPP6	*POF1B*	*FTH1*	*SAE1*
BCAS3	*CTNNA1*	*RPS4X*	*GAS2L3*
ZNF638	*NEBL*	*GAPDH*	*CKAP5*
FOCAD	*SMURF1*	*RPL38*	*TRAPPC9*
MAML3	*LINC00511*	*CSMD1*	*NOP56*
MED13	*MACF1*	*BCAP31*	*ILF3*
MAPK14	*JMJD1C*	*SDC4*	*APOLD1*
PHACTR2	*CAMK2D*	*RPS16*	*OTUD7A*
TNS3	*HERC4*	*HSP90AA1*	*RNF157*
DGKD	*ZNF91*	*MT-ND1*	*CCDC138*
SAMD5	*MUC13*	*ARHGDIA*	*AHI1*
BAZ2B	*CTBP2*	*KRT18*	*GINS1*
OSBPL3	*RALGAPA2*	*RPS28*	*MAD1L1*
SEMA3C	*PDE8A*	*AC243967.1*	*SMC1A*

Table S10. Clinical characteristics of patients by single-cell multiome sequencing

Patient	Age	Gender	Histological type	Location	pTNM: T	pTNM: N	pTNM: M	Stage	MSI status
patient1	52	male	Mucinous Adenocarcinoma	Descending	3	1	0	III	MSI-H
patient2	65	female	Mucinous Adenocarcinoma	Descending	2	1	0	III	MSI-H
