# Supplementary figures and images for: Single-cell multi-omics analysis reveals cancer regulatory elements of transcriptional programs and clinical implications
Source: Cell Death Dis. 2025 Oct 21;16(1):746. doi: 10.1038/s41419-025-08060-7 (PMC12541060; doi:10.1038/s41419-025-08060-7)

Supplementary Figure 11A

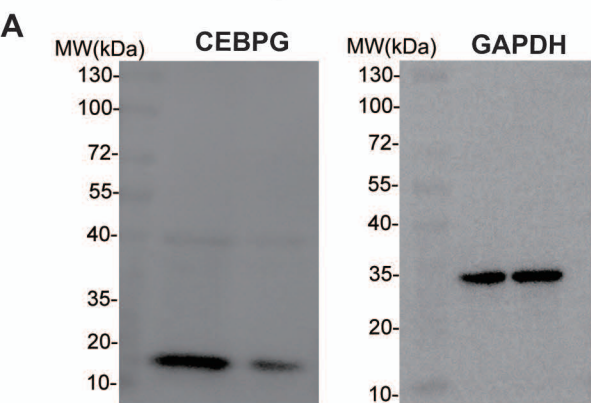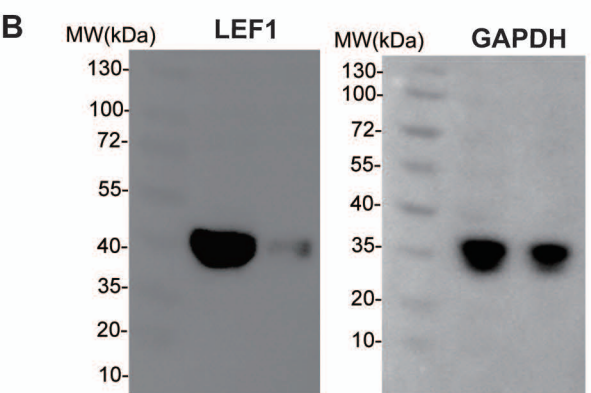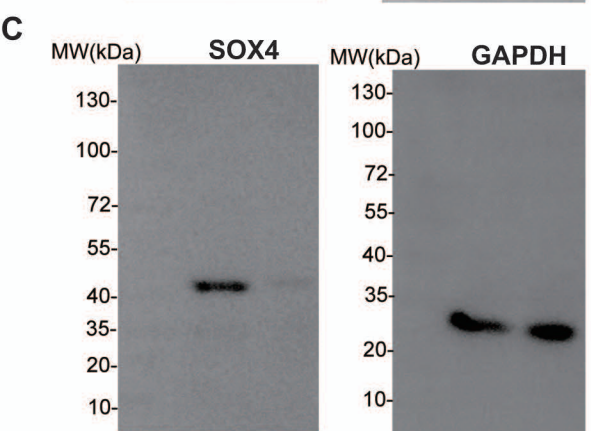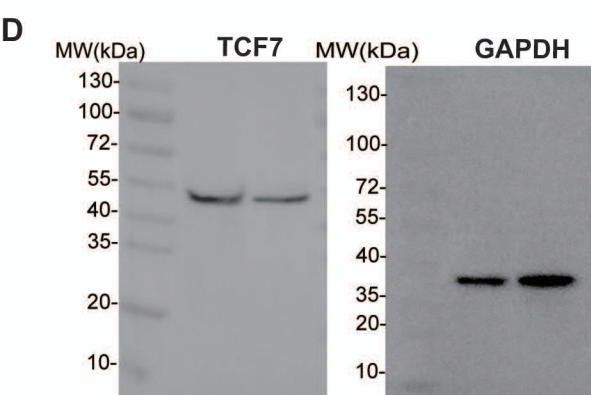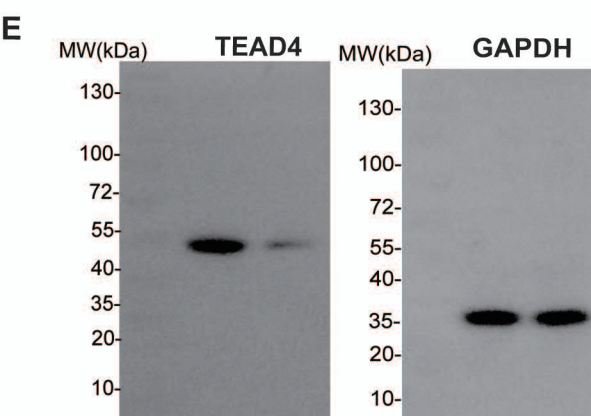

Supplementary Figure 11E

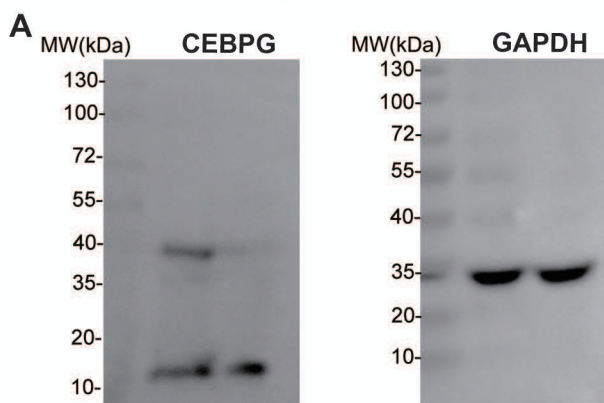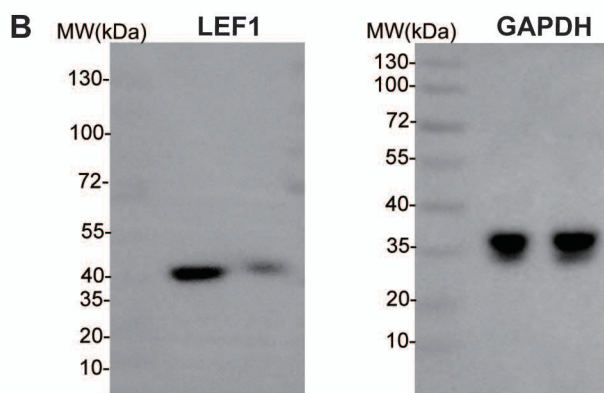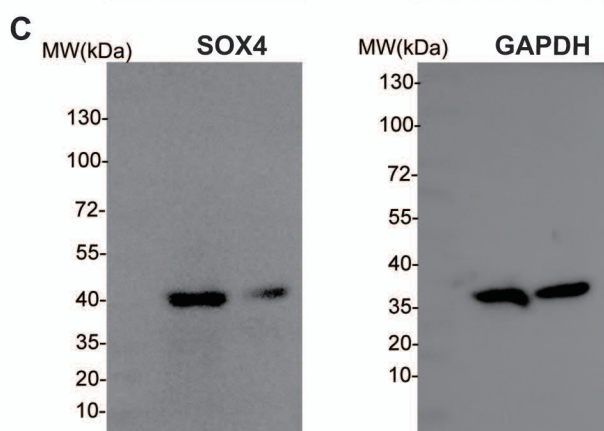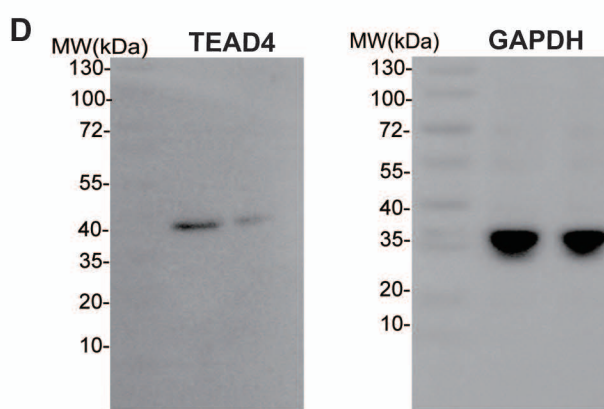

Supplement: Supplementary file 2 — UncroppedWesternBlots [file 41419_2025_8060_MOESM2_ESM.pdf]
